# DIAPH1 mediates progression of atherosclerosis and regulates hepatic lipid metabolism in mice

**DOI:** 10.1038/s42003-023-04643-2

**Published:** 2023-03-17

**Authors:** Laura Senatus, Lander Egaña-Gorroño, Raquel López-Díez, Sonia Bergaya, Juan Francisco Aranda, Jaume Amengual, Lakshmi Arivazhagan, Michaele B. Manigrasso, Gautham Yepuri, Ramesh Nimma, Kaamashri N. Mangar, Rollanda Bernadin, Boyan Zhou, Paul F. Gugger, Huilin Li, Richard A. Friedman, Neil D. Theise, Alexander Shekhtman, Edward A. Fisher, Ravichandran Ramasamy, Ann Marie Schmidt

**Affiliations:** 1 Diabetes Research Program, Department of Medicine, NYU Grossman School of Medicine, NYU Langone Health, New York, NY USA; 2 The Leon H. Charney Division of Cardiology, Department of Medicine, The Marc and Ruti Bell Program in Vascular Biology, NYU Grossman School of Medicine, NYU Langone Health, New York, NY USA; 3 Department of Population Health, Division of Biostatistics, NYU Grossman School of Medicine, NYU Langone Health, New York, NY USA; 4 Biomedical Informatics Shared Resource, Herbert Irving Comprehensive Cancer Center and Department of Biomedical Informatics, Columbia University Irving Medical Center, New York, NY USA; 5 Department of Pathology, NYU Grossman School of Medicine, NYU Langone Health, New York, USA; 6 Department of Chemistry, The State University of New York at Albany, Albany, NY USA

**Keywords:** Lipoproteins, Cardiovascular diseases

## Abstract

Atherosclerosis evolves through dysregulated lipid metabolism interwoven with exaggerated inflammation. Previous work implicating the receptor for advanced glycation end products (RAGE) in atherosclerosis prompted us to explore if Diaphanous 1 (DIAPH1), which binds to the RAGE cytoplasmic domain and is important for RAGE signaling, contributes to these processes. We intercrossed atherosclerosis-prone *Ldlr*^−*/*−^ mice with mice devoid of *Diaph1* and fed them Western diet for 16 weeks. Compared to male *Ldlr*^−*/*−^ mice, male *Ldlr*^−*/*−^ *Diaph1*^−*/*−^ mice displayed significantly less atherosclerosis, in parallel with lower plasma concentrations of cholesterol and triglycerides. Female *Ldlr*^−*/*−^ *Diaph1*^−*/*−^ mice displayed significantly less atherosclerosis compared to *Ldlr*^−*/*−^ mice and demonstrated lower plasma concentrations of cholesterol, but not plasma triglycerides. Deletion of *Diaph1* attenuated expression of genes regulating hepatic lipid metabolism, *Acaca*, *Acacb*, *Gpat2, Lpin1, Lpin2* and *Fasn*, without effect on mRNA expression of upstream transcription factors *Srebf1, Srebf2* or *Mxlipl* in male mice. We traced DIAPH1-dependent mechanisms to nuclear translocation of SREBP1 in a manner independent of carbohydrate- or insulin-regulated cues but, at least in part, through the actin cytoskeleton. This work unveils new regulators of atherosclerosis and lipid metabolism through DIAPH1.

## Abstract (web summary)

Male and female mice deficient in DIAPH1 are protected from the development of experimental atherosclerosis and display reduced cholesterol levels; these changes are linked to the regulation of SREBP1 subcellular localization.

## Introduction

Despite the manifold advances in therapeutic regimens, cardiovascular disease (CVD) remains the leading cause of death in the United States^1,2^. Beyond the panoply of lipid-lowering therapies, seminal benefits for lipid-independent anti-inflammatory treatments have been demonstrated. In the CANTOS trial, treatment with canakinumab, which targets the interleukin-1β pathway, resulted in a significantly lower rate of recurrent cardiovascular events than placebo^3^. A consequence of targeting this immune pathway was the increased risk for significant infection, thereby indicating the overall importance of developing effective and safe adjunctive therapies targeting atherosclerosis.

Previous work implicating the receptor for advanced glycation end products (RAGE) in the progression^4–6^ and regression of atherosclerosis^7^ spurred the current investigation. The cytoplasmic domain of RAGE binds to the formin Diaphanous 1 (DIAPH1), through DIAPH1’s formin homology 1 (FH1) domain^8^. This interaction is important for RAGE ligand-stimulated signal transduction^9,10^. Formins such as DIAPH1 possess diverse functions relevant to the biology of RAGE, such as F-actin polymerization; the organization and regulation of the actin cytoskeleton; cellular migration; signal transduction through the Rho GTPases;^11,12^ and the regulation of RAGE ligand-mediated upregulation of *Egr1* (Early Growth Response 1) via serum response factor (SRF) in hypoxia, factors which induce expression of proinflammatory and prothrombotic factors in oxygen deprivation^13,14^.

Recently, we showed that transplantation of aortic arches from diabetic Western diet (WD)-fed mice devoid of the low-density lipoprotein receptor (*Ldlr*) into diabetic wild-type C57BL/6 J chow-fed mice devoid of *Ager* (the gene encoding RAGE) or *Diaph1* accelerated regression of diabetic atherosclerosis; in parallel, we observed reduced donor atherosclerotic lesion content of neutral lipids, macrophages, oxidative stress and RAGE ligand AGEs, and increased lesional collagen content^7^. Importantly, the aforementioned studies solely probed the effects of transplantation of atherosclerosis-laden aortic arches into an environment of diabetes in normolipidemic mice devoid of *Diaph1*; hence, DIAPH1-dependent mechanisms in the progression of atherosclerosis have never been explored^7^. For this reason, the current investigation was designed to probe if DIAPH1 contributes to progression of atherosclerosis in *Ldlr*^−*/*−^ mice. Here we show that deletion of *Diaph1* protected from progression of atherosclerosis in male and female *Ldlr*^−*/*−^ mice and we demonstrate an unforeseen role for DIAPH1 in the regulation of cholesterol and triglyceride metabolism.

## Results

### DIAPH1 is expressed in human and mouse atherosclerotic lesions

To explore potential roles for DIAPH1 in the progression of atherosclerosis, we began by probing expression of DIAPH1 in atherosclerosis. DIAPH1 was expressed in human atherosclerotic plaques, at least in part in macrophages and smooth muscle cells (SMCs), as illustrated by co-localization of DIAPH1 with CD68 and Smooth Muscle Actin (SMA) epitopes (Supplementary Fig. 1a, b, respectively). Analogous to these findings in human atherosclerosis, DIAPH1 was expressed in macrophages and SMCs of atherosclerotic lesions of *Ldlr*^−*/*−^ mice fed a WD for 16 weeks (Supplementary Fig. 1c, d, respectively). These results set the stage for testing potential roles for DIAPH1 in atherosclerosis through utilization of a mouse model.

### Effect of deletion of *Diaph1* in *Ldlr*^−*/*−^ mice on atherosclerosis and lesion characteristics

**Fig. 1:**
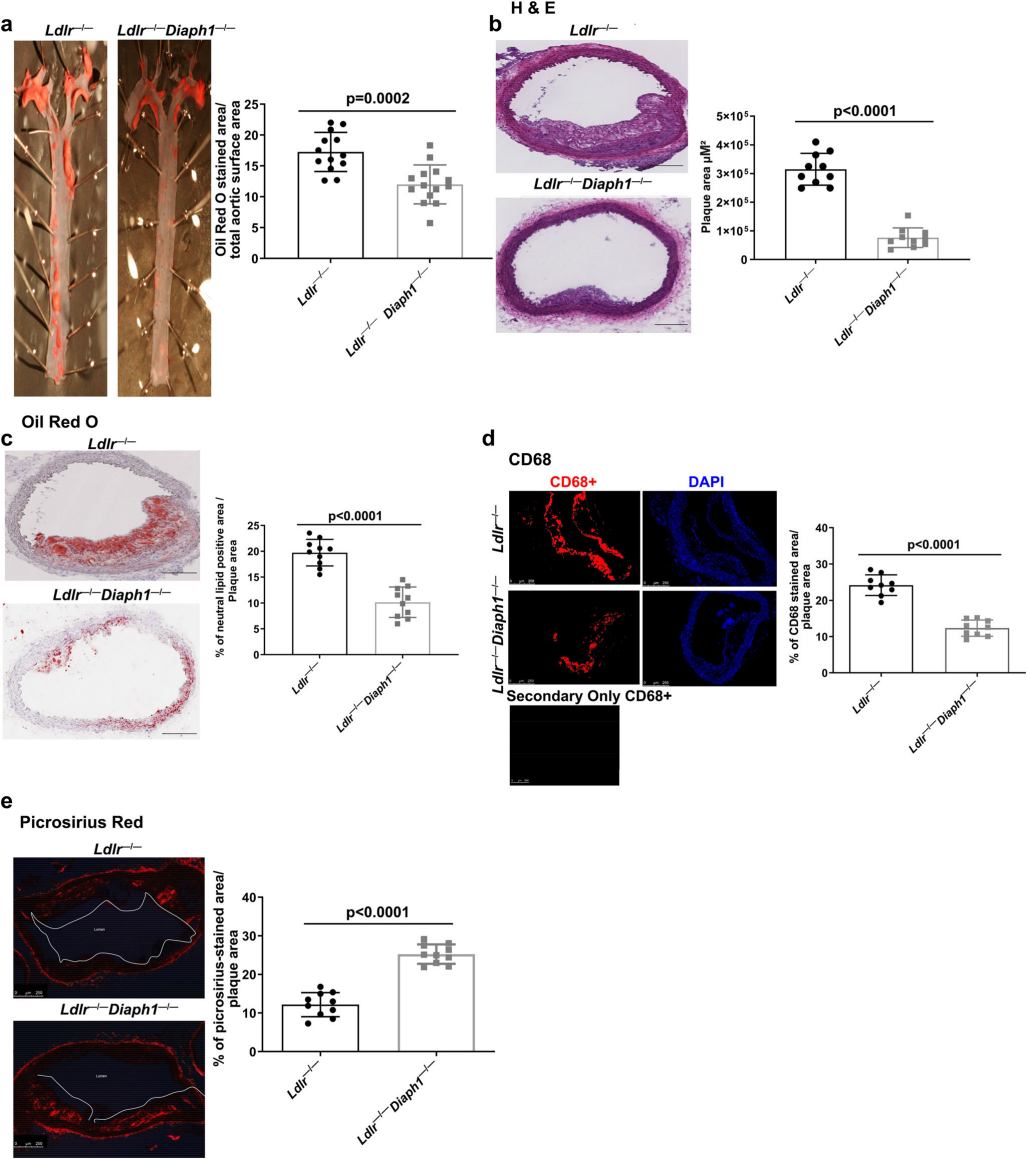
Deletion of *Diaph1* in male *Ldlr*^−*/*−^ mice attenuates the progression of atherosclerosis. *Ldlr* ^−*/*−^ and *Ldlr*^−*/*−^ *Diaph1*^−*/*−^ male mice were fed Western Diet (WD) for 16 weeks. **a** Representative images of *en face* Oil Red O staining of aortas. Quantification of plaque area as percentage of Oil Red O-stained area over total aortic surface area is shown. **b**–**e** Representative images of aortic arch sections are shown and quantified for the following: **b** H&E; **c** Oil Red O; **d** CD68; and **e**, Picrosirius Red. In **d**, the secondary antibody-alone control is shown. Scale bar: 250 µm. The mean ± SEM is reported. The number of independent mice/group is indicated in the figure as individual data points. Statistical analyses regarding testing for the normality of data followed by appropriate statistical analyses were described in Materials and Methods. *P* values were determined by unpaired T-test.

Male mice devoid of the *Ldlr* and *Ldlr*^−*/*−^ mice intercrossed with *Diaph1*^−*/*−^ mice (C57BL/6 J background) were fed WD from age 6 to 22 weeks. After 16 weeks feeding, atherosclerosis was assessed. By *en face* analysis, *Ldlr*^−*/*−^ *Diaph1*^−*/*−^ mice displayed lower neutral lipid content in the aorta, *p* = 0.0002 (Fig. 1a). Next, sections were prepared through the aortic arch. Significantly less atherosclerosis was observed in *Ldlr*^−*/*−^ *Diaph1*^−*/*−^ compared to *Ldlr*^−*/*−^ mice, *p* < 0.0001 (Fig. 1b). Neutral lipid content was significantly lower in the aortic arch atherosclerotic lesions of *Ldlr*^−*/*−^ *Diaph1*^−*/*−^ vs. *Ldlr*^−*/*−^ mice; *p* < 0.0001 (Fig. 1c). Lesional macrophage content was significantly lower in the atherosclerotic plaques of *Ldlr*^−*/*−^ *Diaph1*^−*/*−^ mice vs. *Ldlr*^−*/*−^ mice, *p* < 0.0001 (Fig. 1d). Deletion of *Diaph1* resulted in significantly higher collagen content in the atherosclerotic lesions vs. that observed in *Ldlr*^−*/*−^ mice, *p* < 0.0001 (Fig. 1e). As RAGE and DIAPH1 mediate oxidative and inflammatory stress^15^, which regulate expression of RAGE and RAGE ligands such as AGEs^16^, we determined if deletion of *Diaph1* affected the expression of the ligand-RAGE axis. These investigations revealed that both AGEs and RAGE expression were significantly lower in the lesions of *Ldlr*^−*/*−^ *Diaph1*^−*/*−^ vs. *Ldlr*^−*/*−^ mice, *p* < 0.0001 and *p* = 0.0289, respectively (Supplementary Fig. 1e, f).

We studied a distinct cohort of male mice to assess for the effects of *Diaph1* deletion on atherosclerosis in the *Ldlr*^−*/*−^ background at a different anatomical location, the aortic sinus. As illustrated in Supplementary Fig. 2a, male *Ldlr*^−*/*−^ *Diaph1*^−*/*−^ mice fed WD for 16 weeks displayed significantly less atherosclerosis compared to *Ldlr*^−*/*−^ mice; *p* = 0.0144. In addition to examination of male mice, we performed studies in female mice and found that female *Ldlr*^−*/*−^ *Diaph1*^−*/*−^ mice fed WD for 16 weeks displayed significantly less atherosclerosis at the aortic sinus compared to female *Ldlr*^−*/*−^ mice; *p* = 0.0454 (Supplementary Fig. 2b).

### Effect of deletion of *Diaph1* in *Ldlr*^−*/*−^ mice on inflammation

To begin to account for the mechanisms underlying these differences in atherosclerosis upon global deletion of *Diaph1*, we performed studies to determine the state of inflammation in these mice. First, we performed flow cytometry on the aortic arches of male *Ldlr*^−*/*−^ vs. *Ldlr*^−*/*−^ *Diaph1*^−*/*−^ mice fed WD for 16–18 weeks to characterize macrophage phenotypes. These studies revealed that there were no significant genotype-dependent differences in the percentage of “pro-inflammatory” markers in macrophages, Ly6C and CD14. Similarly, there were no significant genotype-dependent differences in the percentage of “anti-inflammatory” markers in macrophages, CD206 and CD163 (Supplementary Fig. 3a, b and Supplementary Table 1).

Second, we probed for mRNA expression of genes related to inflammation in the aortas of male mice. mRNA transcripts encoding pro-inflammatory *Nos2* and *Tnfa* were significantly lower in *Ldlr*^−*/*−^ *Diaph1*^−*/*−^ vs. *Ldlr*^−*/*−^ mice (*p* < 0.0001 and *p* = 0.0006, respectively) and transcripts encoding anti-inflammatory *Il10* and *Arg1* were significantly higher in *Ldlr*^−*/*−^ *Diaph1*^−*/*−^ vs. *Ldlr*^−*/*−^ mice (*p* = 0.0167 and *p* = 0.0006, respectively) (Supplementary Fig. 4a–d). However, no differences were observed in the mRNA expression of *Ccl2* (*p* = 0.2171) (Supplementary Fig. 4e).

Third, we explored if systemic inflammation was affected by DIAPH1 expression in these mice. There were no significant differences in plasma concentrations of TNF-alpha or IL6 in *Ldlr*^−*/*−^ vs. *Ldlr*^−*/*−^ *Diaph1*^−*/*−^ male mice, *p* = 0.2314 and *p* = 0.8643, respectively. (Supplementary Fig. 4f, g).

Taken together, these data highlight complex effects of *Diaph1* deletion in *Ldlr*^−*/*−^ mice on measures of inflammation; whereas there were no differences in the relative content of “pro-” vs. “anti-“ inflammatory macrophages within the aortic arch or in plasma concentrations of TNF-alpha or IL6, altered mRNA transcript expression of markers of inflammation was noted in the aortas compared to *Ldlr*^−*/*−^ *Diaph1*^−*/*−^ mice. However, mRNA expression of *Ccl2* did not differ by genotype in the aortas. Hence, we next sought to test if DIAPH1 affected concentrations of lipids in *Ldlr*^−*/*−^ mice to uncover mechanisms related to atherosclerosis.

### Effect of deletion of *Diaph1* in *Ldlr*^−*/*−^ mice on plasma concentration of cholesterol and triglyceride

**Fig. 2:**
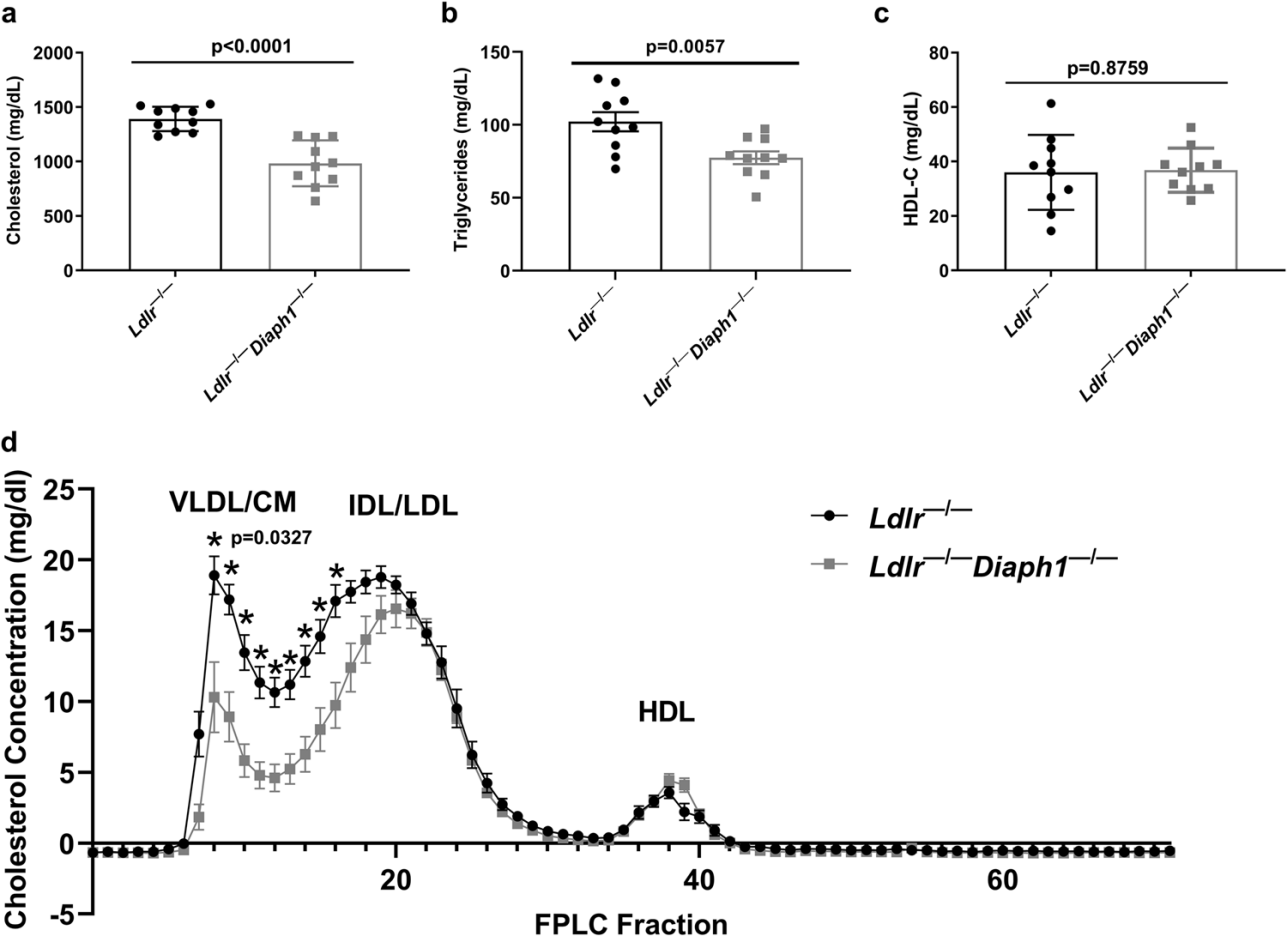
Effect of DIAPH1 on lipid parameters. *Ldlr* ^−*/*−^ and *Ldlr*^−*/*−^ *Diaph1*^−*/*−^ male mice were fed WD for 16 weeks. **a** Concentrations of total plasma cholesterol; **b** concentrations of total plasma triglycerides; **c** concentrations of plasma high-density lipoprotein cholesterol (HDL-C). In **a**–**c** the mean ± SEM is reported. The number of independent mice/group is indicated in the figure as individual data points. **d** Plasma lipoprotein fraction concentrations were measured by Fast Performance Liquid Chromatography (FPLC). CM/VLDL Chylomicron and very-low-density lipoprotein, IDL/LDL Intermediate-density and low-density lipoproteins, HDL High-density lipoprotein. The mean ± SEM is reported from *N* = 6 independent mice/group. Statistical analyses regarding testing for the normality of data followed by appropriate statistical analyses were described in Materials and Methods. *P* values were determined by unpaired T-test.

We examined the concentrations of cholesterol and triglyceride in *Ldlr*^−*/*−^ *Diaph1*^−*/*−^ vs. *Ldlr*^−*/*−^ mice to determine if modulation of lipid profile contributes to the observed differences in atherosclerosis. We retrieved plasma after 16 weeks WD feeding and five hours (h) fasting and observed that plasma cholesterol concentrations were significantly lower in male *Ldlr*^−*/*−^ *Diaph1*^−*/*−^ mice (983 ± 66.6 mg/dl) vs. *Ldlr*^−*/*−^ mice (1,390.8 ± 35.4 mg/dl, respectively), *p* < 0.0001 (Fig. 2a). Similarly, the concentrations of plasma triglyceride were significantly lower in male *Ldlr*^−*/*−^ *Diaph1*^−*/*−^ mice (77.4 ± 13.8 mg/dl) vs. *Ldlr*^−*/*−^ mice (102.2 ± 20.7 mg/dl), *p* = 0.0057 (Fig. 2b). In contrast, there were no differences in plasma concentrations of high-density lipoprotein cholesterol (HDL-C) between genotypes, *p* = 0.8759 (Fig. 2c). We performed fast performance liquid chromatography (FPLC) to establish the nature of the cholesterol particles that accounted for these differences and found that the majority of the reduction in *Ldlr*^−*/*−^ *Diaph1*^−*/*−^ vs. *Ldlr*^−*/*−^ mice was observed in the very low-density lipoprotein (VLDL) and low-density lipoprotein (LDL) fractions, whereas no differences were observed in the HDL fractions (Fig. 2d).

In light of the observation that female *Ldlr*^−*/*−^ *Diaph1*^−*/*−^ mice also displayed significantly less atherosclerosis than female *Ldlr*^−*/*−^ mice, we examined the plasma concentrations of cholesterol and triglyceride. We found that plasma concentrations of cholesterol were significantly lower in female *Ldlr*^−*/*−^ *Diaph1*^−*/*−^ vs. *Ldlr*^−*/*−^ mice (604. 3 ± 21.7 vs. 1094.0 ± 84.1 mg/dl;*p* < 0.0001) (Supplementary Table 2). However, in contrast to the findings in male mice, there were no significant differences in plasma concentrations of triglyceride in female *Ldlr*^−*/*−^ *Diaph1*^−*/*−^ vs. *Ldlr*^−*/*−^ mice (99.9 ± 10.6 vs. 116.9 ± 17.0 mg/dl; *p* = 0.3690) (Supplementary Table 2). Furthermore, there were no genotype-dependent differences in body weight or plasma concentrations of glucose in the female mice, *p* > 0.05 (Supplementary Table 2).

### Deletion of *Diaph1* in male *Ldlr*^−*/*−^ mice reduces hepatic lipid concentrations

**Fig. 3:**
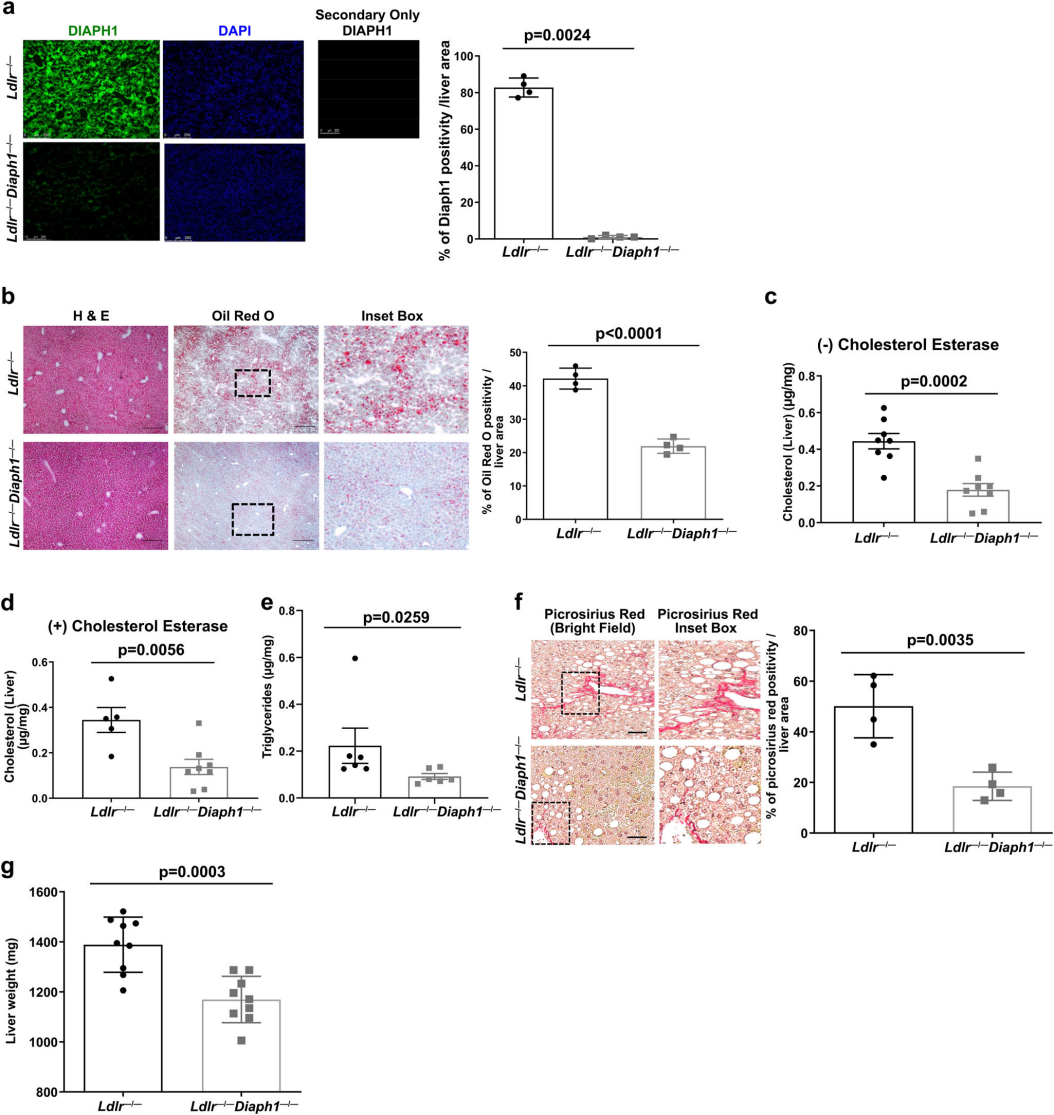
Deletion of *Diaph1* in *Ldlr*^−*/*−^ mice reduces hepatic lipid content and liver fibrosis. *Ldlr*^−*/*−^ and *Ldlr*^−*/*−^ *Diaph1*^*−/−*^ male mice were fed WD for 16 weeks. **a** Representative immunofluorescence staining and quantification of DIAPH1 in the liver of *Ldlr* ^*−/−*^ and *Ldlr*^*−/−*^ *Diaph1*^*−/−*^ male mice. **b** Representative images of H&E and Oil Red O staining in liver and quantification is shown. **c** Quantification of free cholesterol content in liver. **d** Quantification of total cholesterol content in liver. **e** Quantification of total liver triglycerides. **f** Representative images of Picrosirius Red staining in liver and quantification is shown. **g** Quantification of whole liver weight. The mean ± SEM is reported. The number of independent mice/group is indicated in the figure as individual data points. In **a** the secondary antibody–alone control is shown. Scale bars: 250 µm, and inset boxes: 50 µm. Statistical analyses regarding testing for the normality of data followed by appropriate statistical analyses were described in Materials and Methods. *P-*values were determined by unpaired T-test or Wilcoxon rank-sum test depending on if the data passed the Shapiro-Wilk normality test.

To probe DIAPH1-dependent mechanisms in lipid metabolism, we turned our focus to the liver, as it is a major organ responsible for the regulation of lipid metabolism. DIAPH1 is expressed in the liver of *Ldlr*^−*/*−^ mice but not *Ldlr*^−*/*−^ *Diaph1*^−*/*−^ mice fed WD; *p* = 0.0024 (Fig. 3a). In normal mouse liver, hepatocytes demonstrated expression of DIAPH1 (arrowheads, Supplementary Fig. 5a, left panel); cholangiocytes of the bile ducts (BD), sinusoidal mononuclear cells and endothelial cells of the hepatic artery (HA) also expressed DIAPH1 (arrows, Supplementary Fig. 5a, left panel). Control sections with omission of the primary anti-DIAPH1 antibody revealed the absence of staining (Supplementary Fig. 5a, right panel).

In normal human liver, hepatocytes also demonstrated cytoplasmic staining for DIAPH1 and linear dark staining of hepatocyte apical (canalicular) membranes. Dot-like cross-sections of canaliculi were noted (long arrows, Supplementary Fig. 5b, left panel). Some hepatocytes also showed typical features such as intracytoplasmic lipid and binucleation (* and arrowheads, respectively, Supplementary Fig. 5b, left panel). Some mononuclear cells in the sinusoids also showed strong cytoplasmic DIAPH1 staining, but sinusoidal endothelial staining was not noted (Supplementary Fig. 5b, left panel). Furthermore, human bile ducts (BD) showed cytoplasmic staining for DIAPH1, as did nearby hepatocytes. Endothelial cells of portal veins (PV) and hepatic artery (HA) and mononuclear cells within the portal tract also revealed staining for DIAPH1 (Supplementary Fig. 5b, middle panel). Control sections with omission of the primary anti-DIAPH1 antibody revealed the absence of staining (Supplementary Fig. 5b, right panel).

Having established that DIAPH1 was expressed in the liver, at least in part in hepatocytes, we probed for the effects of *Diaph1* deletion in this organ. In the livers of WD-fed male *Ldlr*^*−/−*^ *Diaph1*^*−/−*^ vs. *Ldlr*^*−/−*^ mice, histological analysis revealed a significantly lower neutral lipid content, *p* < 0.0001 (Fig. 3b). The concentrations of free and total cholesterol in the liver were also significantly lower in *Ldlr*^*−/−*^*Diaph1*^*−/−*^ vs. *Ldlr*^*−/−*^ mice, *p* = 0.0002 and *p* = 0.0056, respectively (Fig. 3c, d). The concentrations of hepatic triglyceride were also significantly lower in *Ldlr*^*−/−*^ *Diaph1*^*−/−*^ vs. *Ldlr*^*−/−*^ mice, *p* = 0.0259 (Fig. 3e). Hepatic collagen content was significantly lower in *Ldlr*^*−/−*^ *Diaph1*^*−/−*^ vs. *Ldlr*^*−/−*^ mice, *p* = 0.0035 (Fig. 3f) and liver weight was significantly lower in *Ldlr*^*−/−*^*Diaph1*^*−/−*^ vs. *Ldlr*^*−/−*^ mice, *p* = 0.0003 (Fig. 3g). There were no significant differences in plasma concentrations of alanine aminotransferase (ALT), aspartate aminotransferase (AST), alkaline phosphatase (ALP), total protein (TP), albumin (ALB), albumin/globulin ratio (A/G) or total bilirubin (TBIL) (Supplementary Fig. 5 c–g, i, j). Only plasma globulin (GLOB) concentrations were significantly lower in *Ldlr*^*−/−*^ *Diaph1*^*−/−*^ vs. *Ldlr*^*−/−*^ mice, *p* = 0.0247 (Supplementary Fig. 5h). Collectively, these data demonstrate that deletion of *Diaph1* in male *Ldlr*^*−/−*^ mice resulted in significantly lower concentrations of hepatic cholesterol and triglyceride and lower hepatic mass, fibrosis and lipid deposition compared to that observed in *Ldlr*^*−/−*^ mice expressing *Diaph1*.

### RNA-Sequencing revealed that DIAPH1 plays roles in hepatic lipid metabolism

**Fig. 4:**
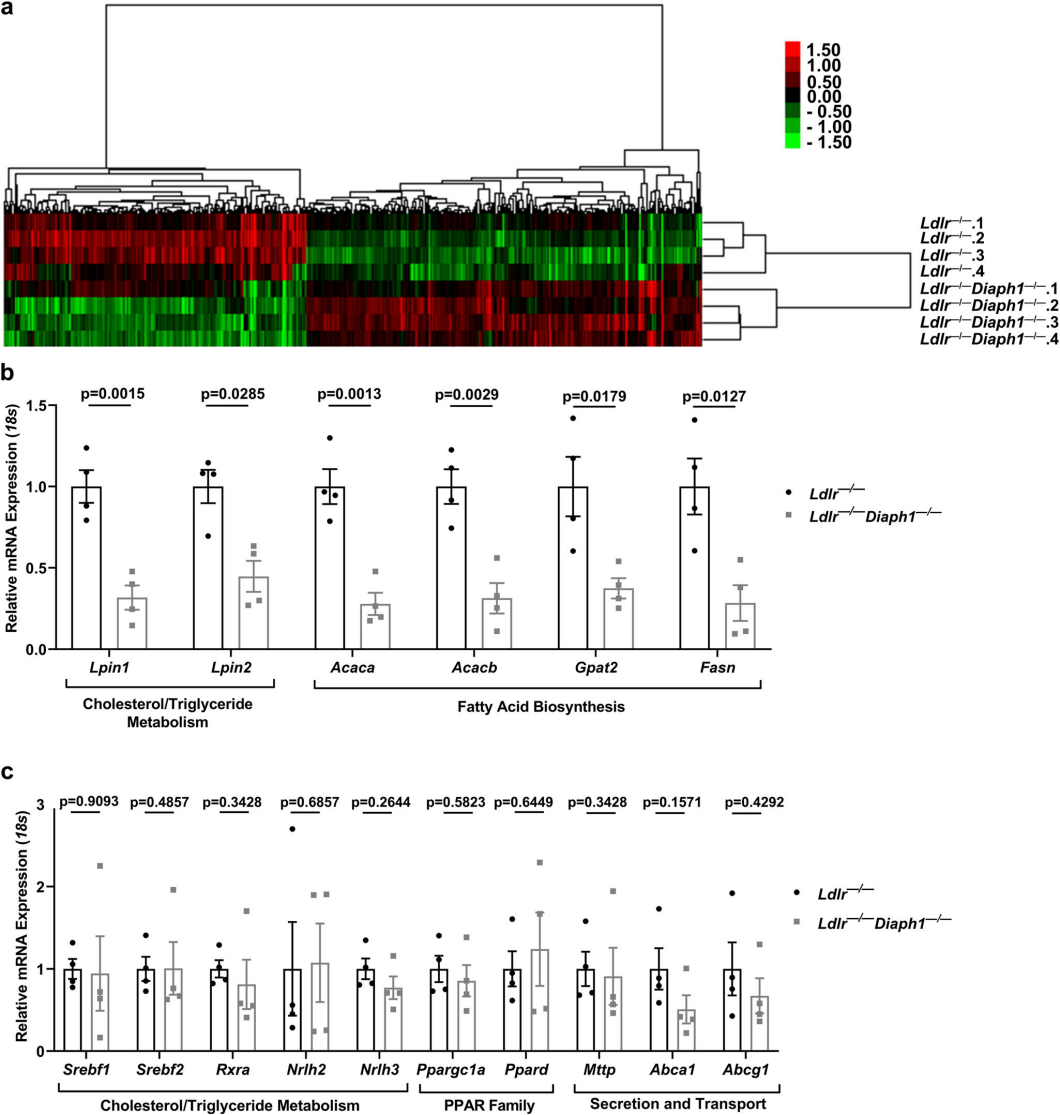
RNA-Sequencing reveals roles for DIAPH1 in regulation of hepatic lipid metabolism. *Ldlr* ^*−/−*^ and *Ldlr*^*−/−*^ *Diaph1*^*−/−*^ male mice were fed WD for 16 weeks. **a** Hierarchical clustering of differentially expressed genes in liver tissue from the indicated *N* = 4 independent mice/group. **b** The expression of the indicated genes identified as differentially expressed in the RNAseq data between *Ldlr* ^*−/−*^ mice vs. *Ldlr*^*−/−*^ *Diaph1*^*−/−*^ mice was determined by RT-qPCR. **c** The expression of the indicated genes identified as not differentially expressed in the RNAseq between *Ldlr* ^*−/−*^ mice vs. *Ldlr*^*−/−*^ *Diaph1*^*−/−*^ mice was confirmed by RT-qPCR. The number of independent mice/group is indicated in the figure as individual data points. Statistical analyses regarding testing for the normality of data followed by appropriate statistical analyses were described in Materials and Methods. *P*-values (in **b**, **c**) were determined by unpaired T test or Wilcoxon rank-sum test depending on if the data passed the Shapiro-Wilk normality test.

To identify the basis of the effect of *Diaph1* deletion on cholesterol and triglyceride metabolism, we performed bulk RNA-sequencing (RNAseq) on the liver tissue of male *Ldlr*^*−/−*^ and *Ldlr*^*−/−*^ *Diaph1*^*−/−*^ mice. Mice were fed WD for 16 weeks and livers were removed after a 5 h fast. A total of 468 genes was differentially expressed with *p*-value ≤ 0.05 and false discovery rate (FDR) ≤ 0.05. A dendrogram and heatmap of these genes is shown in Fig. 4a and the list of the differentially expressed genes is shown in Supplementary data 6. Kyoto Encyclopedia of Genes and Genomes (KEGG) indicated “Glycerophospholipid Metabolism” as among the top differentially regulated pathways (Supplementary Table 3). Reactome pathway analysis (FDR < 0.05) revealed that the top candidate pathway was “Metabolism;” this pathway included multiple differentially expressed genes comparing *Ldlr*^*−/−*^ *Diaph1*^*−/−*^ vs. *Ldlr*^*−/−*^ mice (Supplementary Table 4). Notably, a number of genes within the “Metabolism” pathway were linked to lipid metabolism. An additional 49 Reactome pathways met statistical significance in this data set (Supplementary Table 5). Of these additional pathways, at least 17 were related to lipid metabolism. All of the genes highlighted from the Reactome pathway met the criterion of *p* < 0.05. In agreement with the above results, the Gene Ontology (GO) pathway analysis linked DIAPH1 with lipid metabolism as well, identifying pathways such as “Fatty Acid catabolic process,” “Lysophospholipid transport,” and “Vesicle-mediated cholesterol transport” (Supplementary Table 6).

We next undertook an in-depth analysis of these data to identify putative mechanisms underlying the effects of *Diaph1* on cholesterol and lipid metabolism. With respect to lipid uptake, RNAseq data revealed no significant differences in the mRNA transcripts encoding the fatty acid transporter *Cd36* (Supplementary data 6); although fatty acid binding protein 7, *Fabp7*, was identified as one of the differentially-expressed genes, its expression was higher in the livers of *Ldlr*^*−/−*^*Diaph1*^*−/−*^ vs. *Ldlr*^*−/−*^ mice fed WD (Supplementary data 6), thus not likely accounting for the observed differences in plasma and hepatic cholesterol and triglyceride concentrations between genotypes. There were no differences in expression of genes regulating hepatic fatty acid oxidation, such as *Ppara, Cpt1, Cpt2, Ucp2, Acadl*, or *Acadm* (Supplementary data 6). RNAseq revealed that there were no differences in expression of genes regulating Importins and *Scap*, which have been ascribed roles in the processing and cellular transport of Sterol Regulatory Element Binding Proteins (SREBPs)^17^ (Supplementary data 6). Despite significant differences in plasma and hepatic concentrations of cholesterol in *Ldlr*^*−/−*^*Diaph1*^*−/−*^ vs. *Ldlr*^*−/−*^ mice, RNAseq revealed no significant differences in the expression of the genes linked to cholesterol biosynthesis^18^.

We performed reverse transcription (RT) quantitative PCR (RT-qPCR) analysis on select genes implicated in multiple facets of lipid metabolism using liver tissue retrieved from a distinct cohort of *Ldlr*^*−/−*^ *Diaph1*^*−/−*^ and *Ldlr*^*−/−*^ mice fed WD for 16 weeks. First, as shown in Fig. 4b, consistent with the RNAseq data, mRNA transcripts encoding *Lpin1* and *Lpin2* were significantly lower in the livers of *Ldlr*^*−/−*^ *Diaph1*^*−/−*^ vs. *Ldlr*^*−/−*^ mice, *p* = 0.0015 and *p* = 0.0285, respectively. Genes related to “fatty acid biosynthesis,” including acetyl co-A carboxylase enzymes, *Acaca* and *Acacb*; glycerol-3-phosphate acyltransferase, *Gpat2*, and fatty acid synthase, *Fasn*, were significantly lower in *Ldlr*^*−/−*^*Diaph1*^*−/−*^ vs. *Ldlr*^*−/−*^ livers, *p* = 0.0013, *p* = 0.0029, *p* = 0.0179 and *p* = 0.0127, respectively (Fig. 4b). Second, as shown in Fig. 4c, we verified the RNAseq findings and found no significant differences in genes regulating “cholesterol and triglyceride metabolism”, including the transcription factors *Srebf1*, *Srebf2*, *Rxra*, *Nrlh2* (encodes LXRβ), and *Nrlh3* (encodes LXRα). With respect to genes in the Peroxisome Proliferator-Activated Receptor (PPAR) transcription factor family, RT-qPCR experiments confirmed that there were no differences in *Ppargc1a* and *Ppard* in *Ldlr*^*−/−*^*Diaph1*^*−/−*^ vs. *Ldlr*^*−/−*^ livers (Fig. 4c). In the context of lipid secretion and transport, there were no significant differences in triglyceride and cholesterol transporters such as *Mttp, Abca1*, or *Abcg1*, which confirmed the RNAseq findings (Fig. 4c).

Further, we assessed triglyceride secretion in vivo in chow-fed *Ldlr*^*−/−*^ and *Ldlr*^*−/−*^ *Diaph1*^*−/−*^ mice. After an overnight fast, mice were given an intraperitoneal injection of [^35^S] methionine/cysteine labeling mixture combined with pluronic F127 poloxamer-407, the latter to inhibit lipoprotein clearance from plasma. When comparing *Ldlr*^*−/−*^ vs. *Ldlr*^*−/−*^ *Diaph1*^*−/−*^ mice, there were no significant differences in triglyceride secretion or secretion of apolipoprotein B100 (apoB100) or apolipoprotein B48 (apoB 48) into the plasma (Supplementary Fig. 6a–c).

Collectively, these data indicate that deletion of *Diaph1* in *Ldlr*^*−/−*^ mice downregulated key genes implicated in hepatic lipid metabolism, without affecting the mRNA expression of upstream regulatory transcription factors such as *Srebf1*, *Srebf2* or *Mxlipl*, which encode sterol and carbohydrate regulatory element binding proteins, SREBP1, SREBP2 and Carbohydrate Response Element Binding Protein (ChREBP), respectively.

### Nuclear content of SREBP1, SREBP2 and ChREBP is reduced upon deletion of *Diaph1* in *Ldlr*^−/−^ mice

**Fig. 5:**
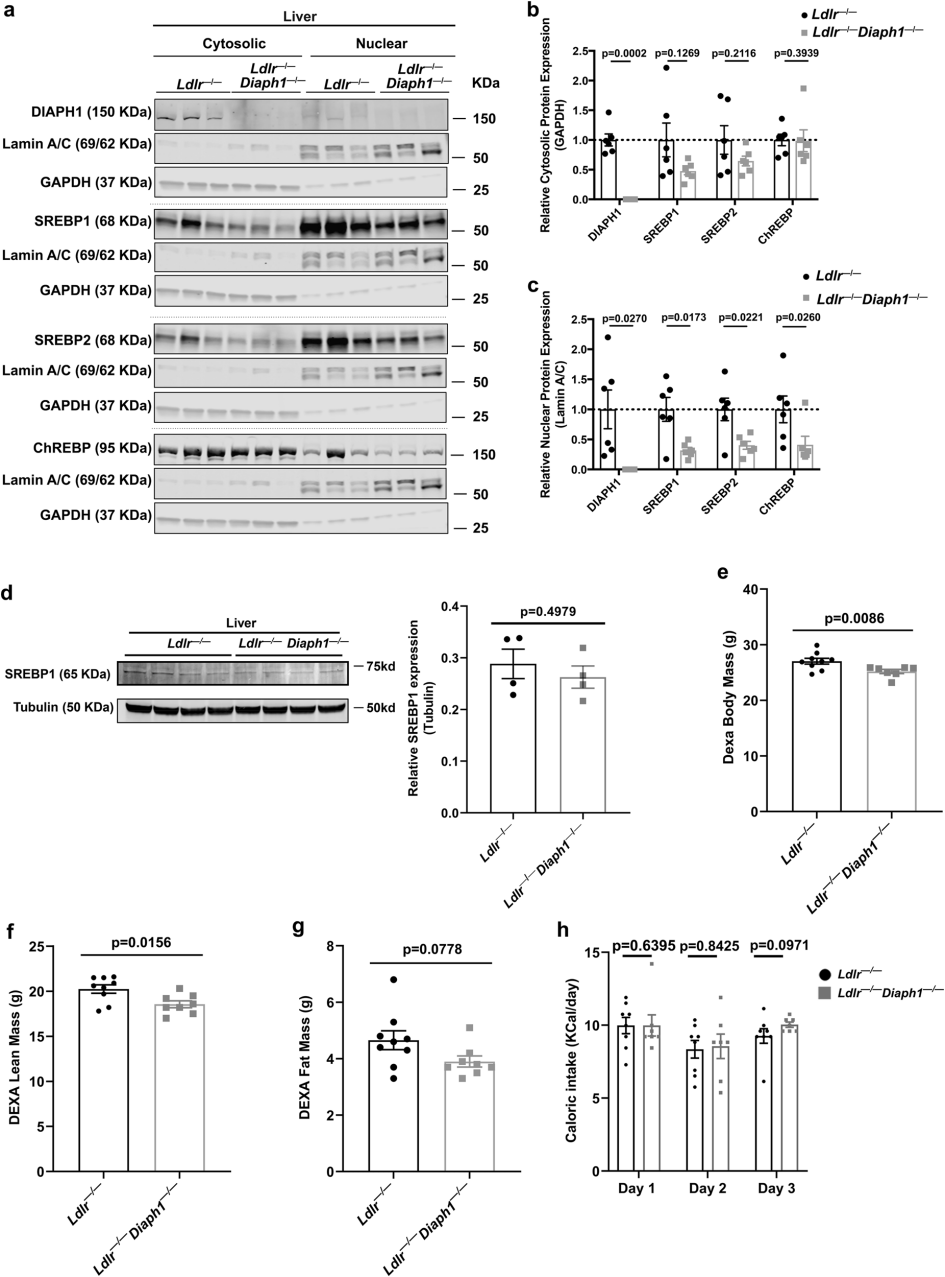
Deletion of *Diaph1* in *Ldlr*^−/−^ mice reduces nuclear content of SREBP1, SREBP2 and ChREBP in liver. *Ldlr* ^*−/−*^ and *Ldlr*^*−/−*^ *Diaph1*^*−/−*^ male mice were fed WD for 16 weeks. **a** Representative Western Blots for the detection of cytosolic and nuclear DIAPH1, SREBP1, SREBP2 and ChREBP performed on liver fractions isolated from the indicated mice. **b** Quantification of cytosolic DIAPH1, SREBP1, SREBP2 and CHREBP, relative to GAPDH. **c** Quantification of nuclear DIAPH1, SREBP1, SREBP2 and ChREBP, relative to Lamin A/C. **d** Representative Western blot and quantification of total SREBP1 normalized to tubulin in total liver of the indicated mice. **e**–**g** DEXA scans were performed for determination of body mass (**e**), lean mass (**f**), and fat mass (**g**). **h** Caloric intake was determined over 3 consecutive days. **i** mRNA expression of the gene encoding RAGE (*Ager*) was determined in the livers of the indicated male mice after 16 weeks WD. The mean ± SEM is reported. The number of independent mice/group is indicated in the figure as individual data points. Statistical analyses regarding testing for the normality of data followed by appropriate statistical analyses were described in Materials and Methods. *P*-values were determined by unpaired T-test or Wilcoxon rank-sum test depending on if the data passed the Shapiro-Wilk normality test.

SREBP1, SREBP2, and ChREBP are central regulators of cholesterol, triglyceride and fatty acid biosynthetic pathways^19^. As RNAseq data illustrated that numerous *Srebf1/Srebf2* or *Mlxipl*-dependent genes were downregulated in the livers of *Ldlr*^*−/−*^ *Diaph1*^*−/−*^ vs. *Ldlr*^*−/−*^ mice, we hypothesized that DIAPH1 affects the activity of these transcription factors independently of changes in their mRNA expression. To test this premise, we prepared cytoplasmic and nuclear extracts from the livers of *Ldlr*^*−/−*^ mice expressing or devoid of *Diaph1*. There were no significant differences in cytoplasmic SREBP1, SREBP2 or ChREBP (normalized to GAPDH) (Fig. 5a, b). In contrast, nuclear SREBP1, SREBP2 and ChREBP (normalized to Lamin A/C) were significantly lower in *Ldlr*^*−/−*^ *Diaph1*^*−/−*^ mice vs. *Ldlr*^*−/−*^ mice livers, *p* = 0.0173, *p* = 0.0221 and *p* = 0.0260, respectively (Fig. 5a, c). Importantly, total SREBP1 protein expression, normalized to tubulin, did not differ in the livers of *Ldlr*^*−/−*^ *Diaph1*^*−/−*^ vs. *Ldlr*^*−/−*^ mice; *p* = 0.4979 (Fig. 5d). To determine if deletion of *Diaph1* in mice devoid of *Ldlr* affected other transcription factors related to metabolism, we probed for CEBPα content and found that cytoplasmic or nuclear amounts of this factor did not significantly differ between *Ldlr*^*−/−*^ vs. *Ldlr*^*−/−*^ *Diaph1*^*−/−*^ livers, *p* = 0.5848 and *p* = 0.6683, respectively (Supplementary Fig. 7a–c). Collectively, these data suggested that DIAPH1 had no effect on total levels of SREBP1 in the liver but that DIAPH1-dependent effects on nuclear content of SREBP1, SREBP2 and ChREBP appeared to be independent of the mRNA transcripts encoding these factors.

### Effect of *Diaph1* deletion in *Ldlr*^−/−^ mice on metabolic factors

As nuclear translocation of the transcription factors SREBP1, SREBP2 and ChREBP may be regulated by metabolic pathways, we investigated multiple factors related to body mass and composition, and glucose/carbohydrate and insulin metabolism.

With respect to body mass and composition, *Ldlr*^*−/−*^ mice devoid of *Diaph1* weighed modestly but significantly less than *Ldlr*^*−/−*^ mice (26.2 ± 0.2 vs. 27.7 ± 0.2 g, respectively), *p* < 0.0001 (Supplementary Table 7). We examined body composition by Dual-Energy X-ray Absorptiometry (DEXA) scanning. Total body mass by DEXA was significantly lower in the *Ldlr*^*−/−*^ *Diaph1*^*−/−*^ vs. *Ldlr*^*−/−*^ mice, *p* = 0.0086; lean mass was significantly lower in the *Ldlr*^*−/−*^ *Diaph1*^*−/−*^ vs. *Ldlr*^*−/−*^ mice, *p* = 0.0156; there were no significant differences in fat mass between genotypes, *p* = 0.0778 (Fig. 5e–g). Caloric intake measured on three consecutive days revealed no significant differences between *Ldlr*^*−/−*^ *Diaph1*^*−/−*^ vs. *Ldlr*^*−/−*^ mice on any of these three days, *p* > 0.05 (Fig. 5h).

We next examined metabolic factors and the potential effects of DIAPH1. We found that there were no statistically significant differences in the concentrations of plasma glucose, serum insulin, serum glucagon, insulin/glucagon ratio or the Homeostatic Model of Insulin Resistance (HOMA-IR) when comparing *Ldlr*^*−/−*^ vs. *Ldlr*^*−/−*^*Diaph1*^*−/−*^ mice, p > 0.05 (Supplementary Table 7). Collectively, these data suggested that DIAPH1-dependent effects on nuclear content of SREBP1, SREBP2 and ChREBP appeared to be independent of classical insulin- and glucose/carbohydrate-related metabolic factors.

### DIAPH1 contributes to atherosclerosis at least in part through effects on lipid not carbohydrate metabolism in *Ldlr*^−/−^ mice

To provide further insight into the question of whether DIAPH1-dependent roles in atherosclerosis were mediated through regulation of lipid vs. glucose/carbohydrate or insulin metabolism, we performed a series of correlation analyses. In male *Ldlr*^*−/−*^ and *Ldlr*^*−/−*^ *Diaph1*^*−/−*^ mice, atherosclerotic lesion area was significantly correlated with lesion neutral lipid content, 0.80 (*p* = 0.0057) and 0.84 (*p* = 0.0026), respectively (Supplementary Table 8a, left) and atherosclerotic lesion area was significantly correlated with lesion macrophage content in both *Ldlr*^*−/−*^ and *Ldlr*^*−/−*^ *Diaph1*^*−/−*^ mice, 0.93 (p = 0.00028) and 0.78 (*p* = 0.0134), respectively (Supplementary Table 8b, left). Further, atherosclerotic lesion neutral lipid content was significantly associated with macrophage content in *Ldlr*^*−/−*^ and *Ldlr*^*−/−*^ *Diaph1*^*−/−*^ mice, 0.93 (p = 0.0003) and 0.82 (p = 0.0068), respectively (Supplementary Table 8c, left).

We tested the associations between the plasma concentrations of cholesterol and triglyceride with atherosclerosis and lesion characteristics. In both *Ldlr*^*−/−*^ and *Ldlr*^*−/−*^ *Diaph1*^*−/−*^ mice, the concentrations of plasma cholesterol significantly correlated with atherosclerotic lesion area (0.83 and 0.86), lesion neutral lipid content (0.93 and 0.97) and lesion macrophage content (0.84 and 0.92), respectively, *p* < 0.01 (Supplementary Table 8d–f, left). In contrast, in *Ldlr*^*−/−*^ and *Ldlr*^*−/−*^ *Diaph1*^*−/−*^ mice, the concentrations of plasma triglyceride did not significantly correlate with atherosclerotic lesion area (0.53 and 0.0183), lesion neutral lipid content (0.51 and 0.22) and lesion macrophage content (0.36 and 0.10), respectively, *p* > 0.05 (Supplementary Table 8g–i, left). Furthermore, in *Ldlr*^*−/−*^ and *Ldlr*^*−/−*^ *Diaph1*^*−/−*^ mice, there were no significant associations between atherosclerotic lesion area and concentrations of glucose (−0.09 and 0.37) or insulin (−0.43 and −0.35), respectively, *p* > 0.05 (Supplementary Table 8j, k, left).

We performed similar analyses in female mice, as illustrated in Supplementary Table 9. The concentrations of plasma cholesterol did not significantly correlate with atherosclerotic lesion area in female *Ldlr*^*−/−*^ mice (−0.12; *p* = 0.7755), but did significantly correlate with atherosclerotic lesion area in the *Ldlr*^*−/−*^ *Diaph1*^*−/−*^ mice (0.70; *p* = 0.0364). As in male mice, the concentrations of plasma triglyceride did not correlate with atherosclerotic lesion area in either *Ldlr*^*−/−*^ or *Ldlr*^*−/−*^ *Diaph1*^*−/−*^ mice (0.003; *p* = 0.9948) and (0.20; *p* = 0.6105), respectively. Similarly, analogous to male mice, in female mice, plasma concentrations of glucose did not correlate with atherosclerotic lesion area in *Ldlr*^*−/−*^ or *Ldlr*^*−/−*^ *Diaph1*^*−/−*^ mice (−0.05; *p* = 0.8955) and (0.43; *p* = 0.2522), respectively (Supplementary Table 9). Collectively, these data suggested that lipid (cholesterol)-driven and not glycemia- or insulin-related mechanisms appeared more likely to contribute to atherosclerosis in these mice. However, these correlation analyses did not discern if there were specific roles for DIAPH1 in these processes.

Hence, we specifically queried if the deletion of *Diaph1* in *Ldlr*^*−/−*^ male mice exerted more prominent effects on the associations with atherosclerosis through lipid or glucose/insulin-related factors when compared to *Ldlr*^*−/−*^ mice. To address this point, we tested if the change in the slope of the lines generated from each set of compared parameters in Supplementary Table 8 was more relevant when comparing male *Ldlr*^*−/−*^ *Diaph1*^*−/−*^ vs. *Ldlr*^*−/−*^ mice. We found that the only genotypic dependence which was significant, both statistically and in terms of effect size, was the dependence of atherosclerotic lesion area on the concentration of plasma cholesterol, *p* = 0.0082 (Supplementary Table 8d, right and Supplementary Fig. 8). The increase of atherosclerotic lesion area per unit plasma cholesterol in *Ldlr*^*−/−*^ *Diaph1*^*−/−*^ mice was approximately one-third of its value in *Ldlr*^*−/−*^ mice. This result implies that about two-thirds of the effect of cholesterol on atherosclerotic lesion formation appears to occur through DIAPH1.

Analogous findings were demonstrated in female mice; as shown in Supplementary Table 9 (right), the only significant genotype-dependent factor (*Ldlr*^*−/−*^ *Diaph1*^*−/−*^ vs. *Ldlr*^*−/−*^) in female mice was the dependence of atherosclerotic lesion area on the concentration of plasma cholesterol (p = 0.0224), but not on the plasma concentrations of triglyceride or glucose.

### Effects of deletion of *Diaph1* in *Ldlr*^−/−^ mice on signal transduction regulatory factors

On account of the evidence linking DIAPH1 to atherosclerosis through lipid metabolism, we examined potential signaling pathways by which DIAPH1 might regulate nuclear translocation of SREBP1, SREBP2 and ChREBP in the livers of the mice under study. First, phosphorylation of AKT has been shown to stimulate nuclear translocation of SREBP1 and SREBP2^20^. We found that the phosphorylated (Ser473) AKT/total AKT was significantly higher in the livers of *Ldlr*^*−/−*^ *Diaph1*^*−/−*^ vs. *Ldlr*^*−/−*^ mice, *p* = 0.0341 (Supplementary Fig. 9a, c), which is in disagreement with our findings that the nuclear SREBP1, SREBP2 and ChREBP were lower in the livers of the *Ldlr*^*−/−*^*Diaph1*^*−/−*^ vs. *Ldlr*^*−/−*^ mice. AKT-related regulation of SREBPs and lipogenesis has been shown to be potentially regulated by mTORC1 pathways^21^. However, there were no significant differences in phosphorylated (Ser2448) mTOR/total mTOR or its downstream target, phosphorylated (Ser240/244) S6/total S6, respectively, when comparing *Ldlr*^*−/−*^ *Diaph1*^*−/−*^ and *Ldlr*^*−/−*^ livers, *p* = 0.2857 and *p* = 0.5905, respectively (Supplementary Fig. 9a, d, e). Furthermore, phosphorylated AMPKα has been shown to affect SREBP nuclear translocation;^22^ we found that there were no differences in phosphorylated (Thr172) AMPK/total AMPK in *Ldlr*^*−/−*^ *Diaph1*^*−/−*^ vs. *Ldlr*^*−/−*^ livers, p = 0.8948 (Supplementary Fig. 9a, b). Overall, these findings indicated that signal transduction pathways responsive to metabolic cues were not significantly different when comparing *Ldlr*^*−/−*^ *Diaph1*^*−/−*^ vs. *Ldlr*^*−/−*^ livers in a manner that would support differences in nuclear translocation of SREBPs or ChREBP.

### Reduced nuclear content of SREBP1, SREBP2 and ChREBP upon deletion of *Diaph1* in *Ldlr*^−/−^ mice and relationship to ROCK, LIMK1, Cofilin pathway

**Fig. 6:**
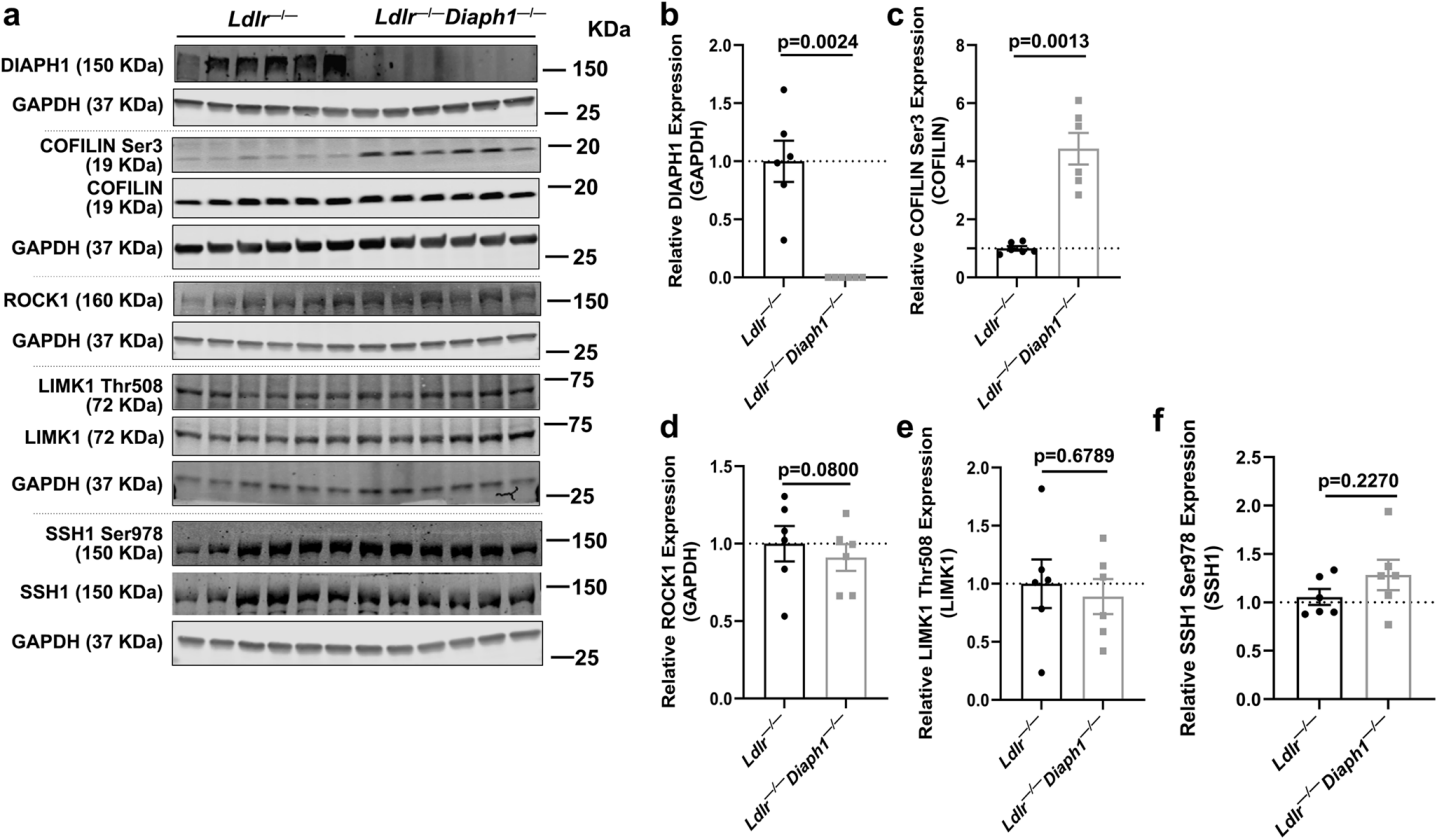
Deletion of *Diaph1* in *Ldlr*^−/−^ mice increases phosphorylated (Ser3)/Cofilin/total Cofilin in liver. *Ldlr* ^*−/−*^ and *Ldlr*^*−/−*^ *Diaph1*^*−/−*^ male mice were fed WD for 16 weeks. **a** Representative Western Blots for the detection of DIAPH1, phosphorylated (Ser3) Cofilin and total Cofilin, ROCK1, phosphorylated (Thr508) LIMK1 and total LIMK1, phosphorylated (Ser978) SSH1 and total SSH1 on total liver lysates isolated from the indicated mice. **b** Quantification of DIAPH1 relative to GAPDH. **c** Quantification of phosphorylated Cofilin (Ser3) relative to total Cofilin. **d** Quantification of ROCK1 relative to GAPDH. **e** Quantification of phosphorylated LIMK1 (Thr508) relative to total LIMK1. **f** Quantification of phosphorylated SSH1 (Ser978) relative to total SSH1. In **b**–**f**, the mean ± SEM is reported. The number of independent mice/group is indicated in the figure as individual data points. Statistical analyses regarding testing for the normality of data followed by appropriate statistical analyses were described in Materials and Methods. *P*-values were determined by unpaired T-test.

Until this point, multiple data described above do not implicate DIAPH1 directly in the classical metabolic factors or signaling pathways that regulate nuclear translocation of SREBP1, SREBP2 and ChREBP, that is, AKT, mTOR/S6 and AMPK pathways. These considerations suggested that distinct DIAPH1-dependent pathways likely regulate the nuclear translocation of these key transcription factors. Accordingly, review of the GO and Reactome pathways highlighted multiple DIAPH1-dependent pathways linked to protein localization, actin cytoskeleton and overall protein transport (Supplementary Tables 5, 6). Previous work highlighted roles for the ROCK-LIMK-Cofilin pathway in nuclear translocation of the SREBPs induced by shear stress^23^. Hence, we probed for these factors in the livers of the mice under study and found that there were no significant differences in ROCK1 or phosphorylated (Thr508) LIMK1/total LIMK1 in *Ldlr*^*−/−*^ vs. *Ldlr*^*−/−*^ *Diaph1*^*−/−*^ liver tissue, *p* = 0.0800 and *p* = 0.6789, respectively. However, phosphorylated (Ser3) Cofilin/total Cofilin was significantly higher in the livers of *Ldlr*^*−/−*^ *Diaph1*^*−/−*^ vs. *Ldlr*^*−/−*^ mice, *p* = 0.0013 (Fig. 6a–e). Note that our studies examine Cofilin1, and not Cofilin2, as the expression of the latter is restricted to striated muscle^24^ and that our RNAseq studies did not reveal differences in expression of the genes encoding Cofilin 1 or 2 (*Cfl1* or *Cfl2*) (Supplementary data 6). To determine if Slingshot1 (SSH1), a phosphatase linked to regulation of phosphorylation of Cofilin^25^ might account for these differences, we probed for this factor in the livers of the mice under study; no differences in phosphorylated (Ser978) SSH1/total SSH1 were observed between genotypes, *p* = 0.2270 (Fig. 6a, f). In addition, RNAseq results from the livers of *Ldlr*^*−/−*^ vs. *Ldlr*^*−/−*^*Diaph1*^*−/−*^ mice revealed that expression of *Ctsd* which encodes Cathepsin D and is linked to regulation of phosphorylation of Cofilin^26^, also did not differ (Supplementary data 6). To determine if DIAPH1-dependent phosphorylation of Cofilin was unique to the liver, we assessed the phosphorylation status in the whole aorta and found that phosphorylated Cofilin (Ser3)/total Cofilin was also significantly higher in the aortas of *Ldlr*^*−/−*^ *Diaph1*^*−/−*^ vs. *Ldlr*^*−/−*^ mice, *p* = 0.0399. (Supplementary Fig. 10a, c). Collectively, these data suggested that in two different organs, liver and aorta, the expression of DIAPH1 was associated with the phosphorylation state of Cofilin 1. As roles for phosphorylation of Cofilin have been ascribed to the regulation of actin polymerization^27–29^, these findings pointed us to new directions for probing DIAPH1-dependent mechanisms in the regulation of the nuclear translocation of the transcription factors under study.

### Silencing of *Diaph1* in Hepa 1-6 cells increases Cofilin phosphorylation, reduces F-actin intensity at baseline and after treatment with RAGE ligands, and affects nuclear translocation of SREBP1

**Fig. 7:**
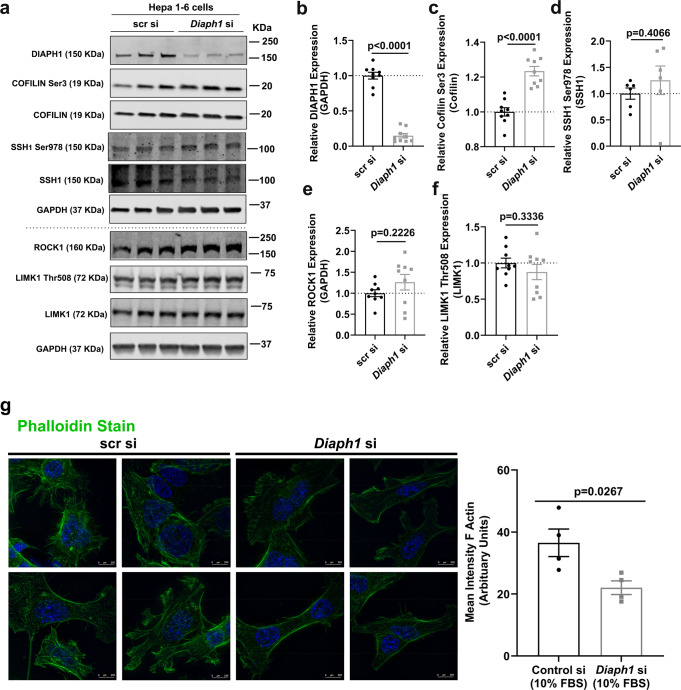
Silencing of *Diaph1* increases phosphorylated (Ser3) Cofilin/total Cofilin and DIAPH1 and RAGE ligands contribute to F-actin polymerization in Hepa 1-6 cells. **a** Representative Western blots for the detection of DIAPH1, phosphorylated (Ser3) Cofilin, total Cofilin, phosphorylated (Ser978) SSH1, total SSH1, ROCK1, phosphorylated (Thr508) LIMK1, total LIMK1 and GAPDH performed on mouse Hepa 1-6 cells after *Diaph1* or scramble control siRNA knockdown. **b** Quantification of DIAPH1 relative to GAPDH. **c** Quantification of phosphorylated (Ser3) Cofilin relative to total Cofilin. **d** Quantification of phosphorylated (Ser978) SSH1 relative to total SSH1. **e** Quantification of ROCK1 relative to GAPDH. **f** Quantification of phosphorylated (Thr508) LIMK1 relative to total LIMK1. **g** Immunofluorescence staining and quantification of the mean intensity of F-actin (phalloidin) in mouse Hepa1-6 cells after *Diaph1* or scramble control siRNA knockdown. Scale bar: 250 µm. The mean ± SEM is reported. The number of independent biological/independent replicates is indicated in the figure as individual data points. Statistical analyses regarding testing for the normality of data followed by appropriate statistical analyses were described in Materials and Methods. *P*-values were determined by unpaired T-test or Wilcoxon rank-sum test depending if data passed the Shapiro-Wilk normality test.

We next focused our experiments in hepatocyte-like cells and utilized mouse Hepa 1-6 cells with siRNA approaches to silence *Diaph1* vs. controls (Fig. 7a, b). Analogous to the effect of deletion of *Diaph1* in livers, silencing of *Diaph1* did not affect expression of ROCK1 (*p* = 0.2226), phosphorylated (Thr508) LIMK1/LIMK1 (*p* = 0.3336), or phosphorylated SSH1 (Ser978)/SSH1 (*p* = 0.4066) (Fig. 7a, d–f). In contrast, phosphorylated (Ser3) Cofilin/total Cofilin was significantly higher in *Diaph1-*silenced Hepa 1-6 cells vs. scramble controls, *p* < 0.0001 (Fig. 7a, c). As the phosphorylation state of Cofilin is linked to actin polymerization, we hypothesized that silencing *Diaph1* in Hepa 1-6 cells might modulate F-actin intensity. We silenced *Diaph1* as illustrated in Hepa 1-6 cells (Supplementary Fig. 11). Compared to scr control, silencing of *Diaph1* significantly reduced F-actin intensity by Phalloidin staining, which reflects the extent of F-actin polymerization, *p* = 0.0267 (Fig. 7g).

**Fig. 8:**
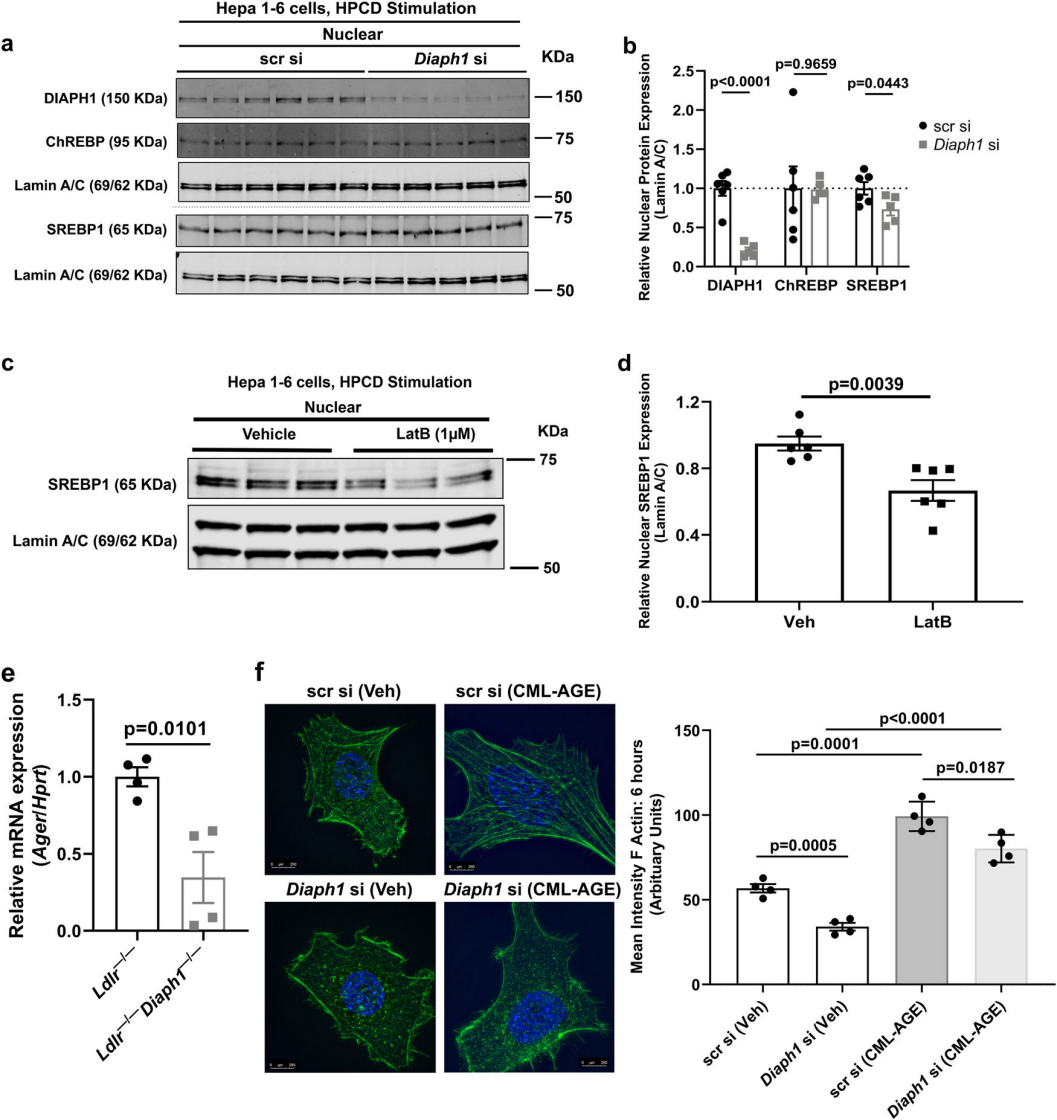
RAGE, DIAPH1, actin organization and SREBP1. **a** Western blots for the detection of nuclear DIAPH1, ChREBP and SREBP1 performed on mouse Hepa 1-6 cells after *Diaph1* or scramble control siRNA knockdown and 30-min sterol depletion with 1% 2-hydroxypropyl-β-cyclodextrin (HPCD). **b** Quantification of nuclear DIAPH1, SREBP1, and ChREBP, relative to Lamin A/C. **c** Western blots for the detection of nuclear SREBP1 performed on mouse Hepa 1-6 cells after 30 min pre-treatment with latrunculin B (LatB; 1 µm) followed by the addition for 30 min of sterol depletion with 1% HPCD. **d** Quantification of nuclear SREBP1 relative to Lamin A/C. **e** mRNA expression of the gene encoding RAGE (*Ager*) was determined in the livers of the indicated male mice after 16 weeks WD. **f** Hepa 1-6 cells bearing scramble control or *Diaph1* siRNA silencing were treated with RAGE ligand CML-AGE (100 µg/ml) or vehicle for 6 h followed by quantification of the mean intensity of F-actin (phalloidin). Scale bar: 250 µm. The mean ± SEM is reported. The number of independent biological/independent replicates is indicated in the figure as individual data points. Statistical analyses regarding testing for the normality of data followed by appropriate statistical analyses were described in Materials and Methods. *P*-values were determined by unpaired T-test or Wilcoxon rank-sum test depending if data passed the Shapiro-Wilk normality test.

As these studies in Fig. 7g indicated that DIAPH1 contributes to F-actin polymerization in Hepa 1-6 cells, we next probed if silencing of *Diaph1* in Hepa 1-6 cells directly affected nuclear translocation of the transcription factors under study. We employed 2-hydroxypropyl-β-cyclodextrin (HPCD) with its sterol chelating properties to elicit nuclear translocation of these factors in the cell-based model^30^. Of note, in Hepa 1-6 cells we were unable to detect SREBP2. Silencing of *Diaph1* significantly reduced nuclear translocation of SREBP1 induced by HPCD compared to scramble control, *p* = 0.0443, but had no effect on nuclear translocation of ChREBP, *p* = 0.9659 (Fig. 8a, b).

Hence, we focused our next experiments on SREBP1. As actin organization has been demonstrated to regulate gene transcription^31^, we tested if nuclear translocation of SREBP1 was linked to the modulation of actin organization in Hepa 1-6 cells. To test this premise, we treated DIAPH1-expressing Hepa 1-6 cells with HPCD in the presence of latrunculin-B, which reduces F-actin polymerization^32^ and found that compared to treatment of Hepa 1-6 cells with HPCD and vehicle, treatment of these cells with HPCD and latrunculin-D significantly attenuated nuclear content of SREBP1; *p* = 0.0039 (Fig. 8c, d).

To address potential mechanisms by which DIAPH1 contributes to actin polymerization in the livers of mice with atherosclerosis, we probed for involvement of the RAGE pathway in *Ldlr*^*−/−*^ mice fed WD for 16 weeks. First, consistent with roles for RAGE in DIAPH1-dependent mechanisms, we found that mRNA expression of *Ager* was significantly lower in the livers of the *Ldlr*^*−/−*^*Diaph1*^*−/−*^ mice vs. the *Ldlr*^*−/−*^ mice fed WD for 16 weeks, *p* = 0.0101 (Fig. 8e). Second, we note that previous studies demonstrated that the ligands of RAGE accumulate in atherosclerosis^7^ and that RAGE ligands induce the formation of RAGE homodimers on the cell surface, which results in the recruitment of DIAPH1^33^. On account of these considerations, we tested if RAGE ligands induced F-actin polymerization and if DIAPH1 was required. As shown in Fig. 8f, treatment of Hepa 1-6 cells bearing scr si with RAGE ligand carboxymethyllysine (CML)-advanced glycation end product (AGE) for 6 h resulted in a significant increase in F-actin polymerization compared to vehicle (Veh) treatment, *p* = 0.0001. However, in *Diaph1-*silenced Hepa 1-6 cells treated with CML-AGE, significantly less F-actin polymerization was noted compared with scr si cells treated with CML-AGE, *p* = 0.0187 (Fig. 8f). Collectively, these data link RAGE ligands to induction of actin polymerization, at least in part through DIAPH1.

## Discussion

DIAPH1 is a large complex molecule whose multiple functions are mediated through its distinct domains^34^. Among these multiple functions, the formin homology 1 (FH1) domain of DIAPH1^9^ binds to the cytoplasmic domain of RAGE. As RAGE has been extensively studied in atherosclerosis, here we sought to test potential roles for DIAPH1 in vascular pathology. Through multiple experimental strategies, this work identified that deletion of *Diaph1* in atherosclerosis-prone mice protected from progression of atherosclerosis and uncovered unanticipated roles for DIAPH1 in the regulation of lipid metabolism. Although this work identified that deletion of *Diaph1* reduced hepatic expression of genes such as *Acaca*, *Acacb*, *Gpat2, Lpin1, Lpin2* and *Fasn* in *Ldlr*^*−/−*^ mice, it was surprising that the mRNA transcripts encoding the master transcriptional regulators of cholesterol and triglyceride metabolism, such as *Srebf1*, *Srebf2*, and *Mlxipl*, as well as genes regulating expression of the PPAR family (*Ppargc1a* and *Ppard*) and the LXR family (*Nrlh2* and *Nrlh3*), did not differ in the livers of *Ldlr*^*−/−*^ mice expressing or devoid of *Diaph1*. In contrast, although there were no differences detected at the mRNA level, studies in the mouse liver underscored that nuclear content of SREBP1, SREBP2 and ChREBP proteins was reduced upon deletion of *Diaph1*. Yet, our data suggested that these processes did not appear to be predominantly regulated by classical metabolic factors that may affect the activities of these transcription factors, such as glucose, carbohydrate or insulin pathways. Indeed, the plasma concentrations of glucose, glucagon and insulin, and their correspondingly regulated signaling pathways, such as AKT, mTOR and AMPK^20–22^, which may govern the activities of the SREBPs and ChREBP, did not differ by DIAPH1 expression status. Furthermore, although body mass was modestly but significantly lower in male *Ldlr*^*−/−*^ *Diaph1*^*−/−*^ vs. *Ldlr*^*−/−*^ mice, in female *Ldlr*^*−/−*^ *Diaph1*^*−/−*^ vs. *Ldlr*^*−/−*^ mice, there were no differences in body mass; in both cases, however, the mice devoid of both the *Ldlr* and *Diaph1* displayed significantly less atherosclerosis and lower plasma concentrations of cholesterol vs. the *Ldlr*^*−/−*^ mice. Nevertheless, we acknowledge that subtle differences in body mass and composition may have contributed to the observed differences in atherosclerosis and lipid metabolism and that studies using distinct *Diaph1*-tissue targeted deleted mice will be required to fully dissect these relative contributions.

Previous studies in endothelial cells linked the RHO-ROCK-LIMK-Cofilin pathway to nuclear translocation of SREBPs and ChREBP; however, the proximate mechanisms were not identified^23^. Here, in both liver tissue and in mouse Hepa 1-6 cells, the present work revealed that genetic deletion or silencing of *Diaph1*, respectively, resulted in significantly higher phosphorylated (Ser3) Cofilin/total Cofilin compared to the respective controls. Cofilin, an actin binding molecule, plays key roles in actin cytoskeleton organization; yet, its biology is complex. Cofilin may bind to F-actin and G-actin and has been implicated in both elongation vs. severing of actin filaments^27–29^. In general, the phosphorylation of Cofilin is linked to its inactivation^35^. As F-actin binding suppresses phosphorylation of Cofilin Ser3^36,37^, reduced F-actin, which was demonstrated by deletion/silencing of *Diaph1*, should cause increased phosphorylation of Cofilin. This is exactly what was observed in our studies in Hepa 1-6 cells. Hence, although we acknowledge that next studies must determine the precise biophysical relationships between DIAPH1, Cofilin and actin, and between DIAPH1, phosphorylated (Ser3) Cofilin and actin, the work presented in this manuscript, nevertheless, suggests new insights into contributory roles for RAGE ligands, DIAPH1 and actin cytoskeleton organization in mechanisms linked to nuclear translocation of SREBP1.

Indeed, precedent for roles for actin organization on nuclear translocation of transcription factors has been demonstrated but the full scope of the upstream regulators of these processes has yet to be identified. Specifically, phosphorylated (Ser3) Cofilin mediates Angiotensin II-stimulated nuclear translocation of NF-kB (p65) in HK2 cells^38^. In other studies, actin organization and Cofilin phosphorylation were linked to nuclear translocation of transcription factors beyond SREBPs and ChREBPs, such as NF-kB and Serum Response Factors complexes^39,40^. The complexity of Cofilin phosphorylation and nuclear translocation of transcription factors was underscored by studies in T cells in which phosphorylated (Ser3) Cofilin was associated with suppression of nuclear translocation of NF-kB components^41^. These findings in T cells are directly analogous to those identified in the present work, suggesting that deletion of *Diaph1* in liver or in Hepa1-6 cells was associated with increased phosphorylation of Cofilin and suppression of nuclear translocation of SREBPs. Certainly, these intricacies of the phosphorylation state of Cofilin and nuclear translocation (or not) of various transcription factors suggests finely-tuned cell type- and stress-dependent regulation of these processes. As noted above, in-depth biophysical studies will be required to further probe these complex mechanisms.

With respect to implications for the RAGE pathway in DIAPH1-dependent regulation of F-actin polymerization, it is important to note that previous studies linked S100A8/A9 (ligands of RAGE) to phosphorylation of Cofilin in MDA-MB-231 breast cancers through RAGE, as revealed in experiments with silencing of *AGER*^42^. In that study, although the content of phospho-Cofilin was not normalized to total Cofilin, the authors did suggest that RAGE ligands contributed to activation of NF-kB and epithelial-mesenchymal transition through actin polymerization pathways^42^. Hence, it will be important to address if/how RAGE ligands/RAGE, DIAPH1 and Cofilin act in concert or independently to regulate nuclear translocation of transcription factors. It is, however, acknowledged that factors beyond modulation of the actin cytoskeleton may underlie the effects of *Diaph1* silencing/deletion on regulation of SREBP1 nuclear translocation in the present work, especially as these first studies were performed in mice globally devoid of *Diaph1*. Hence, future studies in tissue-specific *Diaph1*-deleted mice, such as in hepatocytes, will be required in order to probe the full range of potential DIAPH1-dependent mechanisms.

In the present studies, we found that despite significant reductions in plasma and hepatic concentrations of cholesterol, no significant differences in the expression of genes directly regulating cholesterol biosynthesis were identified. It is possible that DIAPH1-dependent regulation of cholesterol metabolism may be mediated through modulation of the activities of the protein products of the genes regulating cholesterol biosynthesis. It is also possible that DIAPH1-dependent regulation of *Fasn* in the livers of WD-fed *Ldlr*^*−/−*^ mice, as illustrated by our RNAseq data and confirmatory RT-qPCR studies (Fig. 4), may account for these findings. Specifically, through the actions of FASN, acetoacetyl CoA, critical to cholesterol biosynthesis, is formed through the condensation of acetyl CoA and malonyl CoA; processes leading to generation of the fatty acid palmitate. Indeed, it was recently shown that in macrophages, FASN-mediated generation of acetoacetyl CoA contributes to cholesterol synthesis^43^. Hence, it is plausible that DIAPH1-dependent effects on plasma and hepatic cholesterol were mediated, at least in part, through FASN.

Of note, we demonstrated that the aortic arches of male *Ldlr*^*−/−*^ *Diaph1*^*−/−*^ mice displayed significantly lower macrophage content compared to *Ldlr*^*−/−*^ mice. Although lesional macrophage content in both *Ldlr*^*−/−*^ *Diaph1*^*−/−*^ and *Ldlr*^*−/−*^ mice was significantly correlated with atherosclerosis and plasma cholesterol and triglyceride concentrations, our analyses (Supplementary Table 8) probing specific roles for the *Diaph1* genotype in *Ldlr*^*−/−*^ mice suggested that DIAPH1 appears to exert negligible effects on the dependence of atherosclerotic lesion area on macrophage content. These findings were supported by the results of additional experiments reported herein regarding DIAPH1 and its apparently complex effects on inflammation. Specifically, although expression of mRNA transcripts encoding *Nos2* and *Tnfa* was significantly lower, and expression of mRNA transcripts encoding *Il10* and *Arg1* was significantly higher in the aortas of *Ldlr*^*−/−*^ *Diaph1*^*−/−*^ vs. *Ldlr*^*−/−*^ mice, by flow cytometry, there were no genotype-dependent differences in the percentage of pro- vs. anti-inflammatory macrophage markers in the aortic arches and there were no differences in plasma protein concentrations of TNF-alpha or IL6 between the two genotypes. In previous work, pharmacological antagonism of RAGE/DIAPH1 in mice with type 1 and type 2 diabetes resulted in significant reductions in plasma concentrations of TNF-alpha and IL6^44^, thereby emphasizing that the effects of DIAPH1 are dependent on the specific immuno-metabolic characteristics in the discrete milieus. Therefore, although the results of the present work do not support a clear compelling role for modulation of inflammation underlying DIAPH-dependent effects on atherosclerosis, it is important to note the following: First, there is precedent for intrinsic roles for DIAPH1 expression in immune cells and inflammation. For example, our previous investigations demonstrated regulation of hypoxia-stimulated expression of *Egr1* and downstream pro-inflammatory and pro-thrombotic genes in macrophages via serum SRF pathways through DIAPH1^14^. In other work, DIAPH1 was implicated in activation and migration of neutrophils^45^. Second, the present studies were performed in mice globally devoid of *Diaph1*, thereby potentially masking discrete effects of DIAPH1 in other cell types, such as T lymphocytes, in the overall inflammatory milieu. For these reasons, we note that future studies in mice bearing distinct immune cell-specific deletion of *Diaph1* in the absence of *Ldlr*, such as in myeloid cells or T lymphocytes, should directly uncover if myeloid *Diaph1* contributes to atherosclerosis and if such findings are dependent- or independent of differences in plasma/liver content of cholesterol and triglyceride. Such experiments, coupled with single cell/single nucleus transcriptomic studies, may distinguish the effects of DIAPH1 in vascular cells, immune cells or hepatocytes and their cross-talk on lipid metabolism, inflammation and atherosclerosis.

In the present study, we demonstrated that both male and female *Ldlr*^*−/−*^ *Diaph1*^*−/−*^ mice displayed significantly less atherosclerosis at the aortic sinus compared with *Ldlr*^*−/−*^ mice; however, in both sexes, although the mice devoid of both *Ldlr*^*−/−*^ and *Diaph1*^*−/−*^ displayed significantly lower plasma concentrations of cholesterol compared to that observed in *Ldlr*^*−/−*^ mice; only in male but not female *Ldlr*^*−/−*^ *Diaph1*^*−/−*^ mice, were significant reductions in plasma concentrations of triglyceride also observed. These considerations notwithstanding, our correlation analyses revealed that the plasma concentrations of cholesterol, but not triglycerides, significantly correlated with atherosclerotic lesion area in male *Ldlr*^*−/−*^ *Diaph1*^*−/−*^ mice and *Ldlr*^*−/−*^ mice. In female mice, the concentrations of plasma cholesterol correlated with atherosclerotic lesion area only in *Ldlr*^*−/−*^ *Diaph1*^*−/−*^ mice but not *Ldlr*^*−/−*^ mice. It is notable that multiple reports in the literature have identified sex differences in plasma concentrations of cholesterol and/or triglyceride in mice devoid of *Apoe* or the *Ldlr* in various settings, such as during pharmacological interventions or upon superimposed genetic modifications^46–52^.

Finally, a question of great interest has been the dissection of overlapping vs. unique roles for RAGE and DIAPH1 in regulation of signal transduction and gene expression stimulated by RAGE ligands. Multiple studies have demonstrated that deletion of *Ager* reduces atherosclerosis and attenuates vascular inflammation^4–7^. Interestingly, although previous studies did not ascribe lipid regulation functions to RAGE, in fact, closer inspection suggested potential roles for modest RAGE-dependent reductions in cholesterol and triglyceride concentrations in *Ager*-deficient mice^53,54^. Indeed, in a recent study using a model of diabetic donor *Ldlr*^*−/−*^ atherosclerotic plaques transplanted into recipient diabetic wild-type (C57BL/6 J), *Ager*^*−/−*^ mice fed standard chow, concentrations of serum cholesterol were modestly but significantly lower in the *Ager*^*−/−*^ vs. wild-type recipient mice, although there were no differences in concentrations of serum triglyceride^7^. In that study, in male diabetic mice globally devoid of *Diaph1*, modest but significantly lower plasma concentrations of cholesterol and triglyceride were observed compared to wild-type diabetic male mice in the same C57BL/6 J background^7^. Although these mice were diabetic and did not have atherosclerosis, these findings nevertheless suggested that DIAPH1 might contribute to regulation of lipid metabolism; the underlying mechanisms or direct effects on atherosclerosis were not explored in that study. Collectively, the results of the present studies buttress the connections between RAGE and DIAPH1 and suggest that blockade of RAGE/DIAPH1 may be a key adjunctive strategy in therapeutic approaches to atherosclerosis, at least in part through regulation of lipid metabolism.

In summary, our findings unveil new roles for the formin DIAPH1 in the regulation of cholesterol and triglyceride metabolism in a manner independent of direct transcriptional regulation of *Srebf1, Srebf2*, and *Mxlipl*. This work presents a new lens into DIAPH1 functions in the regulation of hepatic lipid metabolism, actin organization, nuclear translocation of SREBP1 and their collective impact on atherosclerosis.

## Materials and Methods

### Animal studies and induction of atherosclerosis

All experiments were performed according to the National Institutes of Health Guide for the Care of Laboratory Animals and the protocols were approved by the Institutional Animal Care and Use Committee at NYU Grossman School of Medicine. Mice (C57BL/6 J background) were deficient for the low-density lipoprotein receptor (*Ldlr*^*−/−*^) (The Jackson Laboratories, Stock No 002207, Bar Harbor ME) or for Diaphanous 1 (DIAPH1) (*Diaph1*^*−/−*^)^55^ backcrossed > 10 generations into *Ldlr*^*−/−*^ (*Ldlr*^*−/−*^ *Diaph1*^*−/−*^). Male and female mice were used in this study. The mice were housed under a 12 h (h) light/dark cycle in a specific pathogen-free facility and had free access to food and water. Mice were fed a Western diet (Research Diets, Inc., D01061401Ci; 0.15% cholesterol) for 16 weeks, starting at 6 weeks of age, unless otherwise stated. At sacrifice, mice were deeply anesthetized with ketamine/xylazine injection. Whole blood was collected from the aorta after a 6 h fast, unless otherwise indicated. For serum isolation, whole blood was allowed to clot in BD Microtainer SST (365967) and collected by centrifugation. For plasma isolation, whole blood was collected with EDTA and then subjected to centrifugation. For tissue collection, mice were perfused through a butterfly needle heart puncture with 1x phosphate-buffered saline (PBS). Mouse aortic arches and roots and livers were removed after perfusion with cold PBS, embedded in optimal cutting temperature (OCT) compound and frozen until analyses (see below).

### Dual Energy X-Ray (DEXA) absorptiometry

DEXA scans were performed using the Lunar PIXImus DEXA instrument (PIXImus, WI). Before each scan session, the instrument was calibrated, mice were weighed, briefly anesthetized via isoflurane inhalation and placed on the scanner. The mean lean mass and fat mass were recorded.

### Serum insulin and glucagon

Serum concentrations of insulin or glucagon were determined using the Insulin ELISA kit (Mercodia Mouse Insulin ELISA #10-1247-10), and the Glucagon Quantikine ELISA kit (R&D Systems #DGCG0), respectively, according to the manufacturer’s instructions. HOMA I-R was calculated as glucose (mMol/L) X Insulin (mIU/L) /22.5.

### Plasma liver function analyses

Plasma was tested for concentrations of alanine aminotransferase (ALT), aspartate aminotransferase (AST), alkaline phosphatase (ALP), total protein (TP), albumin (ALB), globulins (GLOB), albumin/globulins ratio (A/G), and total bilirubin (TBIL). The studies were performed at Charles River Labs following their protocols.

### Plasma lipid concentrations

Plasma total cholesterol (Infinity, Thermo Fisher Scientific, 948541) and triglyceride (Infinity, Thermo Fisher Scientific, TR22421) concentrations were measured according to manufacturers’ directions. HDL-C was measured using a kit from Wako (997-01301).

### Fast-performance liquid chromatography (FPLC)

Plasma samples were filtered using column-filters units of 0.22 µm pores (Millipore, UFC30GV00) and lipoproteins were separated in Superose^TM^6 10/300GL column (GE Healthcare, GE29-0915-96) on a Shimadzu FPLC system using FPLC Buffer (150 mM NaCl, 1 mM EDTA). Equal volumes of filtered plasma were injected into the FPLC system for each mouse. Following separation, 72 fractions were collected for each mouse plasma sample and total cholesterol content was quantified in each single fraction using an enzymatic assay (Wako Diagnostics, 439-17501).

### Plasma ELISAs for detection of inflammation

Plasma was assessed for concentrations of TNF-alpha and IL6 using commercially-available ELISA kits from R&D Systems according to the manufacturer’s instructions: TNF-alpha (MTA00B, Lot # P338520) and IL6 (M6000B, Lot # P342576).

### Liver Triglyceride measurements

Measurement of liver triglyceride content was performed as follows: 100 mg of liver tissue was homogenized in chloroform:methanol using zirconia beads. Lower organic phase containing triglycerides was isolated adding 0.1 M NaCl and 3 M KOH in 65% ethanol. Colorimetric triglyceride assay (Thermo Fisher Scientific, TR22421) was performed in the samples following manufacturer´s recommendations.

### Amplex red assay

Single cell suspensions from mouse livers were prepared by incubating liver tissue with Hyaluronidase I (Sigma, H3506), Collagenase type XI (Sigma, C7657), Collagenase type I (Sigma, C1639) and DNase I (Qiagen, 79254) in PBS with 0.2% BSA and 2 mM EDTA for 30 min at 37 °C^56^. Cells were passed through a 100 µm cell strainer in 1x PBS containing 0.2% BSA and 2 mM EDTA. Mouse liver single cell suspension intracellular cholesterol content was quantified using the Amplex Red Cholesterol Assay Kit (Invitrogen, A12216) following the manufacturer’s recommendations. Total cholesterol was measured using the Amplex Red Assay reagent containing cholesterol esterase. Free cholesterol was measured using the Amplex Red Assay reagent lacking cholesterol esterase. Samples were normalized to total protein levels measured with the Pierce BCA Protein Assay kit (Thermo Scientific, 23225). Plates were read using the Spectra Max Reader^57^.

### Determination of triglyceride, apolipoprotein B100 (apoB100) and apolipoprotein B48 (apoB48) secretion

After an overnight fast, mice were given an intraperitoneal injection of 200 µCi of [^35^S] methionine/cysteine protein labeling mix (NEG772002MC, Perkin Elmer) combined with 1,000 mg/kg pluronic F127 poloxamer-407 (BASF, P2443, Sigma-Aldrich) to inhibit lipoprotein clearance from plasma as previously described^58^. Triglyceride secretion was calculated using plasma collected at 2 h post-injection. Total plasma apoB100 and apoB48 secretion was determined by taking 2 µl of plasma from the 2 h time point and separating the proteins by SDS-PAGE gel electrophoresis followed by densitometric quantification using ImageJ. Plasma from *Apobec1*^*−/−*^ mice (which express only apo B100) was used as control to identify apoB100 and apoB48 bands^59,60^.

### Histological measurement of atherosclerosis

Atherosclerosis was measured by the following three methods: (1) at the aortic arch; (2) at the aortic sinus; and (3) by *en face* analysis of the aorta according to established methods per published recommendations:^61^ Aortic arch and brachiocephalic artery analyses was performed as follows, the arch of the aorta was dissected under a microscope and frozen in OCT embedding medium for serial cryosectioning. To quantify cross-sectional lesion area in the brachiocephalic artery, the Y-shaped piece of brachiocephalic artery was sectioned distally to proximally at 6 μm thickness, starting from the subclavian and carotid arteries. Atherosclerotic lesions lumenal to the internal elastic lamina were quantified in 6 equidistant (100 μm) H&E-stained sections 300–600 μm from the branching point of the brachiocephalic into the carotid and subclavian arteries were collected. The processing of the tissues was handled identically in all of the mice to ensure uniformity. H&E staining is described below. Atherosclerosis at the aortic sinus was assessed as follows, mouse hearts were removed, oriented in a supine position and cut above the midline using a standard blade. The apex of the heart was discarded. Plastic molds were filled with OCT and the heart was placed midline down. The molds were placed on pre-cooled metal racks in dry ice and allowed to cool until they were completely set. The frozen roots were then placed into a −80 °C freezer for storage until sectioning. OCT-embedded hearts were sectioned through the aortic root (6 µm). Four cross sections (50 µm apart) of the aortic sinus were cut per mouse per slide and 5 slides per mouse were prepared. The first section was harvested when the first cusp became visible in the lumen of the aorta. Tissue blocks were cut into sections (10 µm thick). The processing of the tissues was handled identically in all of the mice to ensure uniformity. Sections were fixed with 10% neutral buffered formalin for 7–10 min at room temperature. H&E staining is described in the section to follow.

### H&E Staining of histological sections

Slides were stained with Gill’s Hematoxylin (SigmaAldrich, SLCH6216) by dipping three times into a staining dish. The slides were then rinsed thoroughly in deionized water until run-off was clear. The slides were then stained with a bluing solution for 30 seconds and rinsed with deionizzed water until clear run off. The slides were dehydrated with 70% ethanol solution for 5 min and then dipped once into Eosin to counterstain. Dehydration in 100% ethanol was continued three times for five min each, and in Xylene three times for five min each. Slides were cover-slipped with Eukitt Mounting Medium (Sigma-Aldrich, SKU03989-100ML), covered with a glass coverslip, and left to dry for 24 h. A Keyence imaging microscope (BZ X-800) was used to capture high resolution images of each section. Images were obtained from each slide and the images were quantified using Fiji (Image J) and mean atherosclerotic lesion area of the H&E stained images was calculated per mouse and reported in µm^2^.

### En face lesion area of the mouse aorta

The full descending aorta was dissected, excised, and pinned (Thermo Fisher Scientific, NC9681411); fixed in 4% paraformaldehyde for 10 min. Aorta tissue was stained with Oil Red O.

### Oil Red O staining

Sections were fixed with 10% neutral buffered formalin for 10 min at room temperature. Sections were incubated with propylene glycol for 2 min, then incubated with pre-heated Oil Red O staining solution (American MasterTech, STOROPT) at 60 °C for an additional 8 min. Sections were placed in 85% propylene glycol for 1 min and counterstained with modified Mayer’s hematoxylin (American MasterTech, HXMMHLT) for 1 min, rinsed with water and then mounted with glycerine jelly (Fisher scientific, NC0301797).

### Picrosirius Red (PSR) staining

Sections were fixed with 10% neutral buffered formalin for 10 min at room temperature. Using Picrosirius red stain kit (Polyscience, 24901-500), sections were incubated with Weigert Hematoxylin (Sigma, HT1079) for 10 min, then washed in running tap water for 10 min. Sections were incubated with Solution A 0.2% Phosphomolybdic Acid (Polysciences) for 2 min followed by rinsing in distilled water and then incubated with Solution B (2,4,6 Trinitrophenol + Direct Red 80) (Polysciences) for 60 min. Sections were placed in Solution C (0.1 N Hydrochloric Acid) (Polysciences) for 2 min and dehydrated in ethanol followed by cleaning in xylene and then mounting with xylene-based permount (Fisher, 15820100). Images were taken using the Zeiss Axioplan Wide-field microscope.

### Detection of Aorta AGEs in mouse tissue

Immunohistochemistry was performed on 6 µm OCT-embedded frozen mouse aortic sections using polyclonal rabbit anti-Advanced Glycation End Products (AGE, Abcam, ab23722). Frozen sections were allowed to come to room temperature (from −20 °C). Sections were fixed in 10% Neutral Buffered Formalin for 15 min and then rinsed in distilled water. Antibody incubation and detection was performed on a Ventana Discovery XT (Ventana Medical Systems 750-701) using Ventana reagent buffer and detection kits. Anti-AGE was diluted 1:200 in PBS (Life Technologies Grand Island, New York USA) and incubated for 30 min at 37 °C. AGE was detected with biotinylated horse anti-rabbit (Vector Laboratories, BA-1000), diluted 1:1000 and incubated for 30 min. This was followed by the application of streptavidin-horse radish peroxidase conjugate. The complex was visualized with 3,3 Diaminobenzidine and enhanced with copper sulfate. Slides were washed in distilled water, counterstained with Hematoxylin and mounted with permanent media. Table 1 lists the specific reagents used in this experiment.

**Table 1:** Antibodies for Immunofluorescence and Immunohistochemistry Studies.

Antibody	Company	Catalog Number	Clone#	Dilution	Species	Clonality
**Primary antibodies**						
**Human tissue**						
DIAPH1	Abcam	Ab11173		1:200	Rabbit	Polyclonal
CD68	Dako	M0814		1:200	Mouse	Monoclonal
α-SMA	Millipore Sigma	A2547		1:500	Mouse	Monoclonal
DIAPH1	Abcam	ab129167	EPR7948	1:200	Rabbit	Monoclonal
**Mouse tissue**						
AGE	Abcam	ab23722		1:200	Rabbit	Polyclonal
CD68	Bio-Rad	MCA1957	FA-11	1:1000	Rat	Monoclonal
α-SMA	ThermoFisher Scientific	PA5-18292		1:200	Goat	Polyclonal
RAGE	Genetex	GTX27764		1:150	Goat	Polyclonal
DIAPH1	Abcam	ab129167	EPR7948	1:200	Rabbit	Monoclonal
DIAPH1	Abcam	Ab11173		1:200	Rabbit	Polyclonal

Secondary Antibodies
**Human tissue**
Goat Anti-Rabbit IgG Alexa Fluor 488, Thermofisher Scientific, A11034, 1:200 Dilution
Donkey Anti-Mouse IgG Alexa Fluor 594, Thermofisher Scientific, A21203, 1:200 Dilution
Anti-Rabbit conjugated polymer detection system, Leica BOND Polymer Refine Detection System, DS9800
**Mouse tissue**
Biotinylated horse anti-rabbit IgG, Vector Laboratories, BA-1000, 1:100 Dilution
Biotinylated rabbit anti-rat IgG, Vector Laboratories, BA-4001, 1:1000 Dilution
Donkey anti-goat Alexa Fluor 555, ThermoFisher Scientific, A-32816, 1:200 Dilution
Donkey anti-goat Alexa Fluor 594, ThermoFisher Scientific, A-11058, 1:200 Dilution
Donkey anti-rat Alexa Fluor 555, ThermoFisher Scientific, A-48270, 1:200 Dilution
Goat anti-rabbit Alexa Fluor 488, ThermoFisher Scientific, A11008, 1:200 Dilution

### Detection of aorta CD68

CD68 staining was performed on 10 µm OCT embedded frozen mouse aortic sections using rat anti-mouse CD68 clone FA-11 (AbD Serotech, MCA1957). Frozen sections were brought to room temperature and were fixed in acetone for 15 min, then air dried for 15 min. Antibody incubation and detection was performed on a Ventana Discovery XT (Ventana Medical Systems, 750-701) using Ventana’s reagent buffer and detection kits. Anti-CD68 was diluted 1:1000 in Dulbecco’s PBS from (Life Technologies Grand Island, New York USA) and incubated for 1 h. Sections were incubated for 30 min with mouse-adsorbed, biotinylated rabbit anti-rat IgG (BA-4001, 1:1000, Vector Laboratories) for CD68 detection. This was followed by the application of alkaline phosphatase-streptavidin conjugate (Ventana Medical Systems). The complex was visualized with Naphthol-AS-MX phosphatase (Ventana Medical Systems) and Fast Red complex (Ventana Medical Systems). Slides were washed in distilled water, counterstained with hematoxylin, air dried and then heated for 15 min at 60 °C prior to mounting with permanent media. Table 1 lists the specific reagents used in this experiment.

### Detection of aorta RAGE

Immunohistochemistry was performed on OCT embedded frozen mouse aortic sections. Frozen sections were allowed to come to room temperature. Sections were fixed in acetone for 15 min and then allowed to air dry for 30 min. Sections were blocked for 1 h at room temperature with Protein block serum free ready to use (Dako, X0909). Antibody incubation and detection was performed manually. Goat anti-mouse RAGE (Genetex, GTX27764) was diluted 1:150 in diluent and incubated for 24 h at 4 °C. The following day, the primary antibody was removed and the slides were washed three times with 1x PBS. RAGE was detected with secondary donkey anti-goat Alexa Fluor 594 (ThermoFisher Scientific A-11058) diluted 1:200 and incubated 1 h at room temperature. The secondary antibody was then removed and sections were stained with DAPI, 5 mg/ml, diluted to 1:5000. Slides were washed 3 times with 1x PBS and then washed once with distilled H_2_O. Slides were cover-slip mounted with Prolong gold antifade permanent media (Fisher Scientific, P10144). Appropriate secondary antibody only controls were done in parallel and included with the study sections. The slides were allowed to air dry for 30 min–1 h and stored at 4 °C. Table 1 lists the specific reagents used in this experiment.

### Analysis of human atherosclerosis

Deidentified human coronary artery atherosclerotic specimens with advanced lesions were obtained from CVPath Institute Sudden Death Registry. The study was approved by the CVPath Institutional Review Board (IRB) as an exempt study (#RP0063). The artery segments were fixed in formalin, and 2- to 3 mm segments were embedded in paraffin and cut (5 μm thick). The diagnosis of coronary artery disease and histopathological determination of coronary artery disease were performed by an experienced cardiac pathologist at CVPath Institute. Human sections were deparaffinized using Richard-Allan Scientific 40 Clear-Rite 3 (Thermo Fisher Scientific, 6905) through a series of 100%, 90%, and 70% ethanol for 5 min each, followed by 3 washes. Samples were permeabilized for 10 min in 0.2% Triton X-100 in PBS. Sections were blocked for 1 h in Dako Protein Block, Serum-Free (Agilent, X0909). All primary antibodies were diluted in Antibody diluent (Dako, S3022) and sections were incubated overnight at 4 °C with a combination of antibodies. After three washes, slides were incubated with secondary antibodies diluted in Antibody Diluent for 1 h at 37 °C. Subsequently, slides were washed 3x and stained with 1 μg/mL DAPI (Invitrogen, D3571). Slides were cover-slipped mounted with Prolong gold antifade permanent media (Thermo Fisher Scientific, P10144). Primary antibodies used: rabbit anti-DIAPH1 (Abcam, Ab11173, 1:200 dilution), mouse anti-CD68 (Dako, M0814, 1:200 dilution), and Mouse anti-αSMA (Millipore Sigma, A2547, 1:500 dilution). Different combinations of secondary antibodies utilized: goat anti-rabbit Alexa Fluor 488 (Thermo Fisher Scientific, A-11034, 1:200 dilution), and donkey anti-mouse Alexa Fluor 594 (Thermo Fisher Scientific, A-21203, 1:200 dilution). Appropriate secondary antibody only controls were done in parallel and included with the study sections. Table 1 lists the specific reagents used in this experiment.

### Immunohistochemistry of human and mouse liver for detection of DIAPH1

Deidentified normal human liver specimen (52 year-old male subject) was obtained from the Center for Biospecimen and Research Development at the NYU Grossman School of Medicine. The study was approved by the NYU Grossman School of Medicine Institutional Review Board (IRB), study number s16-00122. The sample was fixed in 10% neutral buffered formalin (Fisher Chemical, SF100-4) for 48 h at room temperature. The sample was then dehydrated through graded ethanols and xylene and infiltrated with paraffin in a Leica ASP300S automated tissue processor. Mouse samples were fixed and processed on a Leica Peloris automated processor. Five µm thick sections were cut onto superfrost slides and deparaffinized for immunostaining on a Leica BondRX automated stainer, following the manufacturer’s instructions. In brief, sections underwent epitope retrieval for 20 min at 100 °C with Leica Biosystems ER2 solution (pH9, AR9640). Slides were incubated with rabbit monoclonal anti-DIAPH1 antibody (Abcam, ab129167, clone EPR7948), diluted 1:200 for 30 min at room temperature or with antibody diluent alone (no primary control) and followed by anti-rabbit HRP-conjugated polymer and the substrate diaminobenzidine (Leica BOND Polymer Refine Detection System, DS9800). Sections were counter-stained with hematoxylin and scanned at a 40x magnification (pixel size 0.22 μm) on a Nanozoomer (Model C9600-12, NDP.scan v3.1.9) whole slide scanner (Hamamatsu) and the image files uploaded to the NYU Grossman School of Medicine’s OMERO Plus image data management system (Glencoe Software). Table 1 lists the specific reagents used in this experiment.

### Image quantification

ImagePro Plus 7.0 software was used to determine CD68 + , Oil Red O + , H&E + , AGEs + , Picrosirius red + , RAGE + , *En face* Oil Red O + areas and calculated as percent of total plaque area. Quantification of mean intensity of phalloidin was completed by measuring and then averaging the mean intensity of 6–8 images per animal using ImageJ. Quantification of intensity within specific positive area was completed using ImageJ by restricting the intensity measurement to only areas within an automated pre-set threshold mask of another signal channel. Averages for each sample were calculated and was used for statistical analysis.

### Flow cytometry

To phenotype aortic macrophages, atherosclerosis was induced by feeding Western diet (Research Diets, Inc., D01061401Ci 0.15% cholesterol) for 18 weeks. After a 5 h fast, the mice were subjected to deep anesthesia with ketamine and xylazine; the aortas were perfused with PBS and the aortic arches were collected from experimental mice and whole aortas were retrieved from control mice; the latter control mice were used for the flow cytometry standardization studies. Aortas were digested using a cocktail of liberase TH (0.88 mg/ml, Roche, 5401151001), deoxyribonuclease (DNase) I (58 µg/ml, Sigma, DN25), and hyaluronidase (99 µg/ml, Sigma, H3506) in HBSS with 0.5% BSA and 1 mM of calcium solution. Digestion was performed for 15 min at 37 °C using the program m_37SDK1 in the Gentle Macs dissociator (Miltenyi). Single cell suspensions were filtered through 100 µM filters (Fisher Scientific, #22363549) and pelleted by centrifugation (400 x g for 5 min at 4 °C). Aortic single cell suspensions were live/dead stained with Fixable Blue Dead Cell Stain Kit (Invitrogen L34961) and blocked with CD16/32 (See Table below for the full list / details of all antibodies used in the flow cytometry studies) for 30 min at 4 °C in the dark. To identify aortic macrophages and monocytes, cells were further incubated with antibodies recognizing CD45R, CD3ε, CD170/Siglec-F and Ly-6G (FITC), as well as CD45 (AF700), CD11b (APC-Cy7), CD206 (BV650), CD163 (PE), CD14 (BV421) and Ly-6C (BV510) (See Table below) for 30 min at 4 °C in the dark. Aortic macrophages were identified as UV-, Lin-, CD45 + and CD11b + , and further characterized as CD206^high^, CD163^high^, CD14^high^ and Ly-6C^high^ subsets. Cells were acquired on a LSRII UV (BD Biosciences) and analyzed with FlowJo 10.8.1 (BD Biosciences). For all flow cytometry experiments, UltraComp eBeads compensation beads (Invitrogen, 01-2222-42) were used to set single stain compensation, and FMO controls were used to set all gates. Table 2 lists the specific reagents used in this experiment.

**Table 2:** Antibodies for Flow Cytometry.

Antibody	Company	Catalog Number	Clone#	Dilution	Fluoro-phores	Species	Clonality
CD16/32	Biolegend	101302	93	1:25		Rat	Monoclonal
CD45	Biolegend	103128	30-F11	1:100	Alexa Fluor 700	Rat	Monoclonal
CD11b	BD Biosciences	557657	M1/70	1:100	APC-Cy7	Rat	Monoclonal
CD45R/B220	Biolegend	103206	RA3-6B2	1:100	FITC	Rat	Monoclonal
CD3ε	Biolegend	100306	145-2C11	1:100	FITC	Armenian Hamster	Monoclonal
CD170 (Siglec-F)	Biolegend	155504	S17007L	1:100	FITC	Rat	Monoclonal
LY-6G	Biolegend	127606	1A8	1:100	FITC	Rat	Monoclonal
CD206	Biolegend	141723	C068C2	1:100	Brilliant Violet 650	Rat	Monoclonal
LY-6C	Biolegend	128033	HK1.4	1:100	Brilliant Violet 510	Rat	Monoclonal
CD14	Biolegend	123329	Sa14-2	1:100	Brilliant Violet 421	Rat	Monoclonal
CD163	Invitrogen	12-1631-82	TNKUPJ	1:100	PE	Rat	Monoclonal

### Cultured mouse hepatocellular carcinoma cells (Hepa 1-6)

The mouse hepatocellular carcinoma cell line Hepa 1-6 was purchased from American Type Culture Collection (ATCC® CRL-1830™). Cells were propagated in Dulbecco’s Modified Eagle Medium (DMEM), supplemented with 10% fetal bovine serum (FBS) and 1% penicillin/streptomycin in a humidified atmosphere of 5% CO_2_ at 37 °C.

#### Detection of Phalloidin and treatment of Hepa 1-6 cells with Carboxymethyllysine (CML)-AGE

Hepa1-6 cells were plated in a 6 well plate the day before silencing/knockdown approaches. Cells were serum-starved and placed in Opti-MEM Reduced Serum Medium (Gibco, Thermo Fisher Scientific, 31985062) for 2 h. Cells were transfected using MISSION siRNA Transfection Reagent (MilliporeSigma, S1452) and 75 nM of scrambled siRNA (QIAGEN, 1022076) or *Diaph1* #8 Flexitube siRNA (QIAGEN, SI02732289) for 48 h in 10% FBS DMEM. Cells were treated with CML-AGE (100 µg/ml) or vehicle for 6 h. Cells were fixed in 4% PFA at 4 °C for 10 min and washed 3x TBST. Cells were then blocked for 1 h in Licor Odyssey Blocking Buffer (Li-cor, P/N 927-40100). Phalloidin 400x solution was diluted to 1x (ThermoFisher Scientific, A12379) and placed on cells at room temperature for 1 h. Cells were then washed with TBST 3 times and then stained with DAPI (1:5000) and coverslipped with fluorescence mounting medium (Dako, S3023).

### Silencing of *Diaph1* and pharmacological treatments

Hepa 1-6 cells were plated into 6 well tissue culture plates the day before application of silencing and scramble reagents. Cells were serum-starved and placed in Opti-MEM Reduced Serum Medium (Gibco, Thermo Fisher Scientific, 31985062) for 2 h. Cells were transfected using MISSION siRNA Transfection Reagent (Millipore Sigma, S1452) and 75 nM of scrambled siRNA (Qiagen, 1022076) or *Diaph1* #8 Flexitube siRNA (Qiagen, SI02732289) for 48 h in 10% FBS DMEM. For sterol depletion, cells were transfected as previously described in 1% FBS DMEM and then incubated in DMEM (no FBS), supplemented with 1% 2-hydroxypropyl-β-cyclodextrin (HPCD) (Sigma, C0926-5G) for 30 min^30^. Where indicated, cells were pre-treated for 30 min with Latrunculin B (LatB) (Sigma, L5288), 1 µM, vs. equal volumes of vehicle (DMSO), prior to the subsequent addition of HPCD for 30 min. Hence, the total incubation time for LatB was 60 min.

### Subcellular fractionation of livers and Hepa 1-6 cells

Cytosolic and nuclear fractions from snap-frozen livers were obtained using the Qproteome Nuclear Protein Kit (Qiagen, 37582), following the manufacturer´s recommendations. Nuclear fractions from Hepa 1-6 cells were obtained using the NE-PER™ Nuclear and Cytoplasmic Extraction Reagents (Thermo Fisher Scientific, 78833), following the manufacturer´s recommendations. The enriched cytosolic and nuclear fractions were confirmed by immunoblotting for GAPDH and Lamin A/C, respectively.

### RNA sequencing and bioinformatics

RNAseq was performed on the top left lobe of livers from a. WT, fed chow; b. *Ldlr*^*−/−*^ fed Western diet; and c, *Ldlr*^*−/−*^ *Diaph1*^*−/−*^, fed Western Diet (*N* = 4 independent mice/group). RNA was isolated with RNeasy® Plus Micro Kit (Qiagen, #74004). RNA integrity numbers (RIN) were measured using an RNA 6000 Pico Kit in 2100 Bioanalyzer (Agilent). Samples with a minimum RIN of 9.0 were prepared for sequencing using the NuGEN Ovation RNA-Seq system v2 reagents for cDNA preparation and the Ovation Ultralow DR Multiplex system for the adapter ligation step. 30 M 50-nucleotide, single-end reads were sequenced on an Illumina 2500 HiSeq using v4 chemistry (Illumina) at the NYU Langone Health Genome Technology Center. Fastq files were aligned to the mm10 assembly of the human genome with Rsubread^62^ and gene expression was quantified with featureCounts^63^. Data were deposited in the Gene Expression Omnibus^64^, with accession number GSE156403. Differential expression was analyzed using weighted Limma-voom^65^ with a significance cutoff of the Benjamini-Hochberg FDR ≤ 0.05. Genes with FDR ≤ 0.05 were analyzed further: iPathwayGuide^66^ was used to analyze differential expression in terms of the KEGG^67^ database using Signaling Pathway Impact Analysis^68^ and the Biological Process Gene Ontology^69^. WebGestalt^70^ was used to analyze differential expression in terms of Reactome pathways^71^. Hierarchical clustering was performed on genes with FDR ≤ 0.05 using Cluster 3.0^72^. Dendrograms and heatmaps were displayed using JavaTreeview^73^.

### Quantitative reverse transcription PCR experiments

Total RNA from livers or aortas was extracted using the RNeasy Plus Mini kit (Qiagen, 74136). cDNA was synthesized using iScript cDNA Synthesis Kit (Bio-Rad, 1708891) and amplified using TaqMan assays using a 7300 Real-Time PCR System (Applied Biosystems, Thermo Fisher Scientific). Table 3 lists the specific reagents used in this experiment.

**Table 3:** Reagents for quantitative reverse transcription PCR studies.

Probe	Thermo Fisher Catalog #
*Abca1*	Mm00442646_m1
*Abcg1*	Mm00437390_m1
*Acaca*	Mm01304257_m1
*Acacb*	Mm01204691_m1
*Ager*	Mm00545815_m1
*Arg1*	Mm00475988_m1
*Ccl2*	Mm00441242_m1
*Diap1*	Mm00492170_m1
*Fasn*	Mm00662319_m1
*Gpat2*	Mm01335101_m1
*Hprt*	Mm03024075_m1
*Il10*	Mm01288386_m1
*Lpin1*	Mm00550511_m1
*Lpin2*	Mm00522390_m1
*Mlxipl*	Mm02342723_m1
*Mttp*	Mm00435015_m1
*Nos2*	Mm00440502_m1
*Nr1h2*	Mm00437265_m1
*Nr1h3*	Mm00443451_m1
*Ppia*	Mm02342430_g1
*Ppard*	Mm00803184_m1
*Ppargc1a*	Mm01208835_m1
*Rxra*	Mm00441185_m1
*Srebf1*	Mm00550338_m1
*Srebf2*	Mm01306292_m1
*Tnf*	Mm00443258_m1
*18* *s*	Hs99999901_s1

### Western blotting

Protein concentrations from previously described subcellular fractions or total protein lysates extracted with RIPA buffer (Cell Signaling, 9806 S) supplemented with protease inhibitor (Thermo Fisher Scientific, A32953) and phosphatase inhibitor (Thermo Fisher Scientific, A32957) cocktails, were quantified using a Pierce BSA Protein Assay kit (Thermo Fisher Scientific, 23225). A total of 30 μg of protein were separated by 7.5% or 4–20% polyacrylamide gel (Bio-Rad, 456-1026 or 456-8096) electrophoresis, transferred to 0.2 μm pore size nitrocellulose membranes (Bio-Rad, 170-4270), blocked for 1 h at room temperature with blocking buffer (LI-COR, 927-60001), and incubated overnight at 4 °C with primary antibody (See Below). After washing, membranes were incubated with secondary antibody (See Below) for 1 h at room temperature. Protein signals were visualized with the Odyssey Imaging System (LI-COR) detection system. Densitometric analysis was performed using Image Studio software (LI-COR). Cytosolic and total protein amounts were calculated relative to GAPDH or Tubulin, nuclear protein amounts were calculated relative to Lamin A/C. Phosphorylated protein was normalized to the respective total protein. Table 4 lists the specific reagents used in this experiment.

**Table 4:** Antibodies for Western Blotting and ELISAs for Detection of TNF-alpha and IL6.

Antibody	Company	Catalog Number	Dilution	Species	Clonality
DIAPH1	BD Biosciences	610849	1:1000	Mouse	Monoclonal
SREBP-1	Santa Cruz	sc-13551	1:2000	Mouse	Monoclonal
SREBP-1	Novus Biologicals	NB100-2215	1:1000	Rabbit	Polyclonal
SREBP-2	Santa Cruz	sc-13552	1:2000	Mouse	Monoclonal
ChREBP	Novus Biologicals	NB400-135	1:2000	Rabbit	Polyclonal
ChREBP	Cell Signaling	58069 S	1:1000	Rabbit	Polyclonal
Lamin A/C	Cell Signaling	2032 S	1:2000	Rabbit	Polyclonal
AKT Ser473	Cell Signaling	9271 S	1:2000	Rabbit	Polyclonal
AKT	Cell Signaling	9272 S	1:2000	Rabbit	Polyclonal
AMPKα Thr172	Cell Signaling	2535 T	1:1000	Rabbit	Polyclonal
AMPKα	Cell Signaling	5831 T	1:2000	Rabbit	Polyclonal
mTOR Ser2448	Cell Signaling	5536 T	1:2000	Rabbit	Polyclonal
mTOR	Cell Signaling	2972 S	1:2000	Rabbit	Polyclonal
S6 Ser240/244	Cell Signaling	2215 S	1:2000	Rabbit	Polyclonal
S6	Cell Signaling	2317 S	1:2000	Rabbit	Polyclonal
ROCK1	Cell Signaling	4035 T	1:2000	Rabbit	Polyclonal
LIMK1 Thr508	Abcam	Ab194798	1:1000	Rabbit	Polyclonal
LIMK1	Cell Signaling	3842 S	1:2000	Rabbit	Polyclonal
SSH1 Ser978	ECM Biosciences	SP3901	1:1000	Rabbit	Polyclonal
SSH1	ECM Biosciences	SP1711	1:1000	Rabbit	Polyclonal
COFILIN Ser3	Cell Signaling	3313 T	1:1000	Rabbit	Polyclonal
COFILIN	Cell Signaling	5175 T	1:2000	Rabbit	Polyclonal
C/EBPα	Cell Signaling	8178 S	1:2000	Rabbit	Polyclonal
GAPDH	Santa Cruz	sc-32233	1:5000	Mouse	Monoclonal
Tubulin	Millipore Sigma	T5168	1:25000	Mouse	Monoclonal
IRDye 680RD Goat anti-mouse	LI-COR	925-68070	1:5000	Goat	Polyclonal
IRDye 800RD Goat anti-rabbit	LI-COR	925-32211	1:5000	Goat	Polyclonal
IL6 ELISA	R&D Systems	M6000B			
TNF-alpha ELISA	R&D Systems	MTA00B			

### Statistics and reproducibility

Sample sizes were based on our previous studies in which similar experimental endpoints were tested. Analyses were performed using GraphPad Prism 8.2.0. Data are presented as mean ± SEM. Normality of the data was assessed using the Shapiro-Wilk normality test. A nonparametric test was performed when data did not follow a normal distribution. Independent 2-sample *t*-tests (2 sided) were used to assess the difference between 2 groups of samples (Mann-Whitney *U* tests were used instead if normality was violated). For over 2 groups, One-way ANOVA was used, and Tukey’s or Holm-Šídák post hoc test for pairwise comparisons or comparisons of selected groups was performed, respectively. Kruskal-Wallis test with post-hoc Dunn’s test was performed instead if the normality test was not passed. Pearson’s correlation coefficient was assessed to evaluate the associations between 2 variables. The dependence of atherosclerotic lesion area, and related quantities such as macrophage content, on molecular concentrations such as cholesterol and glucose, as a function of *Diaph1* genotype, was estimated by ANCOVA (ANalysis of COVAriance)^74^ (Supplementary Tables 8, 9). Normality was tested using the Shapiro-Wilk test^75^, and qqplots^76^. All of the analysis presented in Supplementary Tables 8, 9 were performed in R^77,78^. *P* < 0.05 was denoted statistically significant.

### Reporting summary

Further information on research design is available in the Nature Portfolio Reporting Summary linked to this article.

## Supplementary information

Supplementary Information

Description of Additional Supplementary Files

Supplementary Data 1

Supplementary Data 2

Supplementary Data 3

Supplementary Data 4

Supplementary Data 5

Supplementary Data 6

Reporting Summary

## Data Availability

Primary data are available in Supplementary Data 1–6. RNAseq data are deposited to NCBI GEO GSE156403. Any materials reported in this research are available through Material Transfer Agreement (MTA) with NYU Grossman School of Medicine.
