# Supplementary material for: DIAPH1 mediates progression of atherosclerosis and regulates hepatic lipid metabolism in mice
Source: Commun Biol. 2023 Mar 17;6:280. doi: 10.1038/s42003-023-04643-2 (PMC10023694; doi:10.1038/s42003-023-04643-2)
Supplement: Supplementary file 1 — Supplementary Information [file 42003_2023_4643_MOESM1_ESM.pdf]

Supplementary Information

DIAPH1 Mediates Progression of Atherosclerosis and Regulates Hepatic Lipid Metabolism in Mice

Laura Senatus[1^], Lander Egaña-Gorroño[1^], Raquel López-Díez[1^], Sonia Bergaya[2], Juan Francisco Aranda[1], Jaume Amengual[2], Lakshmi Arivazhagan[1], Michaele B. Manigrasso[1], Gautham Yepuri[1], Ramesh Nimma[1], Kaamashri Mangar[1], Rollanda Bernadin[1], Boyan Zhou[3], Paul F. Gugger[1], Huilin Li[3], Richard A. Friedman,[4] Neil D. Theise[5], Alexander Shekhtman[6], Edward A. Fisher[2], Ravichandran Ramasamy[1], Ann Marie Schmidt[1]*

^ These authors contributed equally

[1]Diabetes Research Program, Department of Medicine, New York University Grossman School of Medicine, NYU Langone Medical Center, New York, NY, USA; [2]The Leon H. Charney Division of Cardiology, Department of Medicine, The Marc and Ruti Bell Program in Vascular Biology, New York University Grossman School of Medicine, NYU Langone Medical Center, New York, NY, USA; [3]Departments of Population Health (Biostatistics) and Environmental Medicine, New York University Grossman School of Medicine, NYU Langone Medical Center, New York, NY, USA; [4]Biomedical Informatics Shared Resource, Herbert Irving Comprehensive Cancer Center and Department of Biomedical Informatics, Columbia University Irving Medical Center, New York, NY, USA; [5]Department of Pathology, New York University Grossman School of Medicine, NYU Langone Medical Center, New York, USA; [6]Department of Chemistry, The State University of New York at Albany, Albany, NY, USA

Supplementary Figures

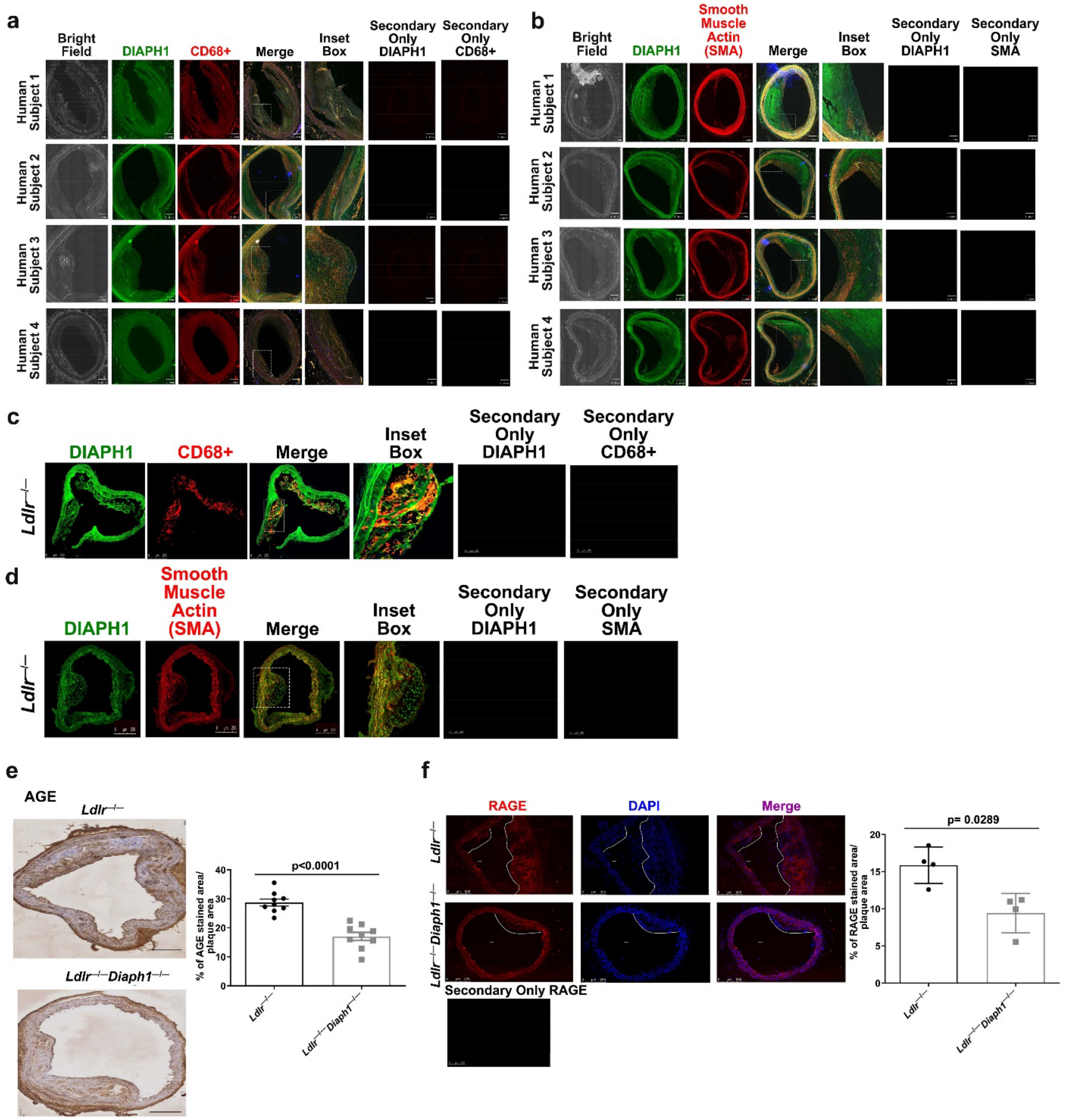

Supplementary Figure 1. DIAPH1 is expressed in human and murine atherosclerotic lesions. **a,** Immunofluorescence staining and colocalization of DIAPH1 and CD68 in N=4 non-diabetic human coronary atherosclerotic lesions. Scale bar: 1.0 mm; and inset box, 50 µm. **b,** Immunofluorescence staining and colocalization of DIAPH1 and smooth muscle alpha actin (SMA) in N=4 non-diabetic human coronary atherosclerotic lesions. Scale bar: 1.0 mm; and inset box, 50 µm. **c,** Immunofluorescence staining and colocalization of DIAPH1 and CD68 in aortic arch of male *Ldlr*$^{-/-}$ mice fed Western Diet (WD) for 16 weeks. Scale bar: 250 µm; and inset box: 50 µm. **d,** Immunofluorescence staining and colocalization of DIAPH1 and SMA in aortic arch of

male *Ldlr*$^{-/-}$ mice fed WD for 16 weeks. Scale bar: 250 µm; and inset box: 50 µm. In a-d, the secondary antibody–alone control is shown. **e,f,** *Ldlr* $^{-/-}$ and *Ldlr*$^{-/-}$ *Diaph1*$^{-/-}$ male mice were fed WD for 16 weeks. **e,** Representative staining and quantification of Advanced Glycation End Products (AGEs) in aortic arch sections. Scale bar: 250 µm. **f,** Representative immunofluorescence staining and quantification of the Receptor of Advanced Glycation End Products (RAGE) in aortic arch sections. Scale bar: 250 µm. The secondary antibody–alone control is shown in the figure. The mean ± SEM is reported. The number of independent mice/group is indicated in the figure as individual data points. Statistical analyses regarding testing for the normality of data followed by appropriate statistical analyses were described in Materials and Methods. *P* values were determined by unpaired t test and Wilcoxon rank-sum test depending on if the data passed the Shapiro-Wilk normality test.

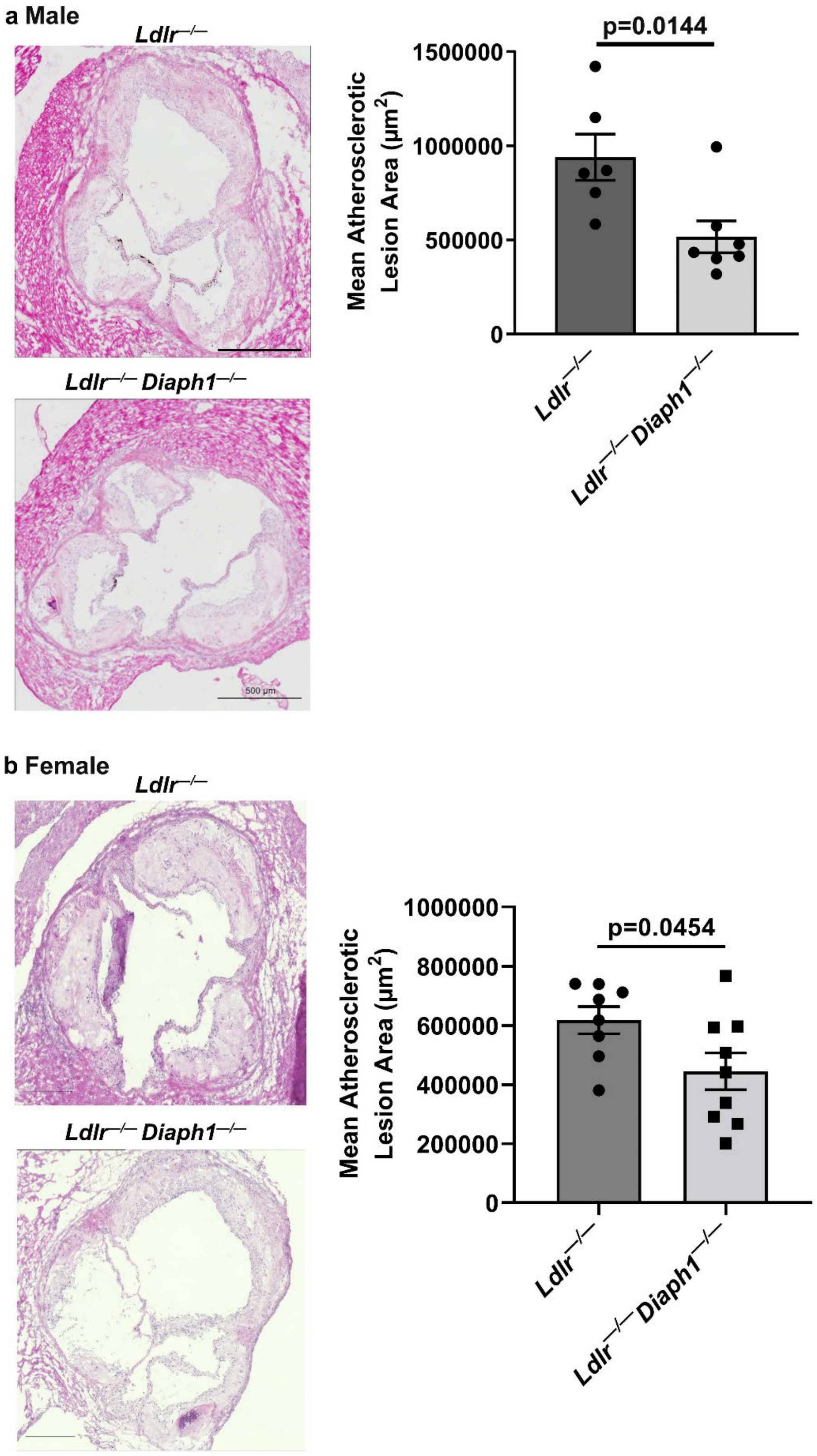

Supplementary Figure 2. Effect of deletion of *Diaph1* in *Ldlr*$^{-/-}$ mice on atherosclerosis at the aortic sinus. Male (**a**) and female (**b**) *Ldlr*$^{-/-}$ mice were fed WD for 16 weeks; at sacrifice,

the mice were perfused and sections were taken from the aortic sinus for assessment of atherosclerosis by H&E staining. Scale bar: (a), 500 µm and (b), 250 µm. The mean ± SEM is reported. The number of independent mice/group is indicated in the figure as individual data points. Statistical analyses regarding testing for the normality of data followed by appropriate statistical analyses were described in Materials and Methods. According to these analyses, *P* values were determined by unpaired T test (a) and Wilcoxon rank sum test (b).

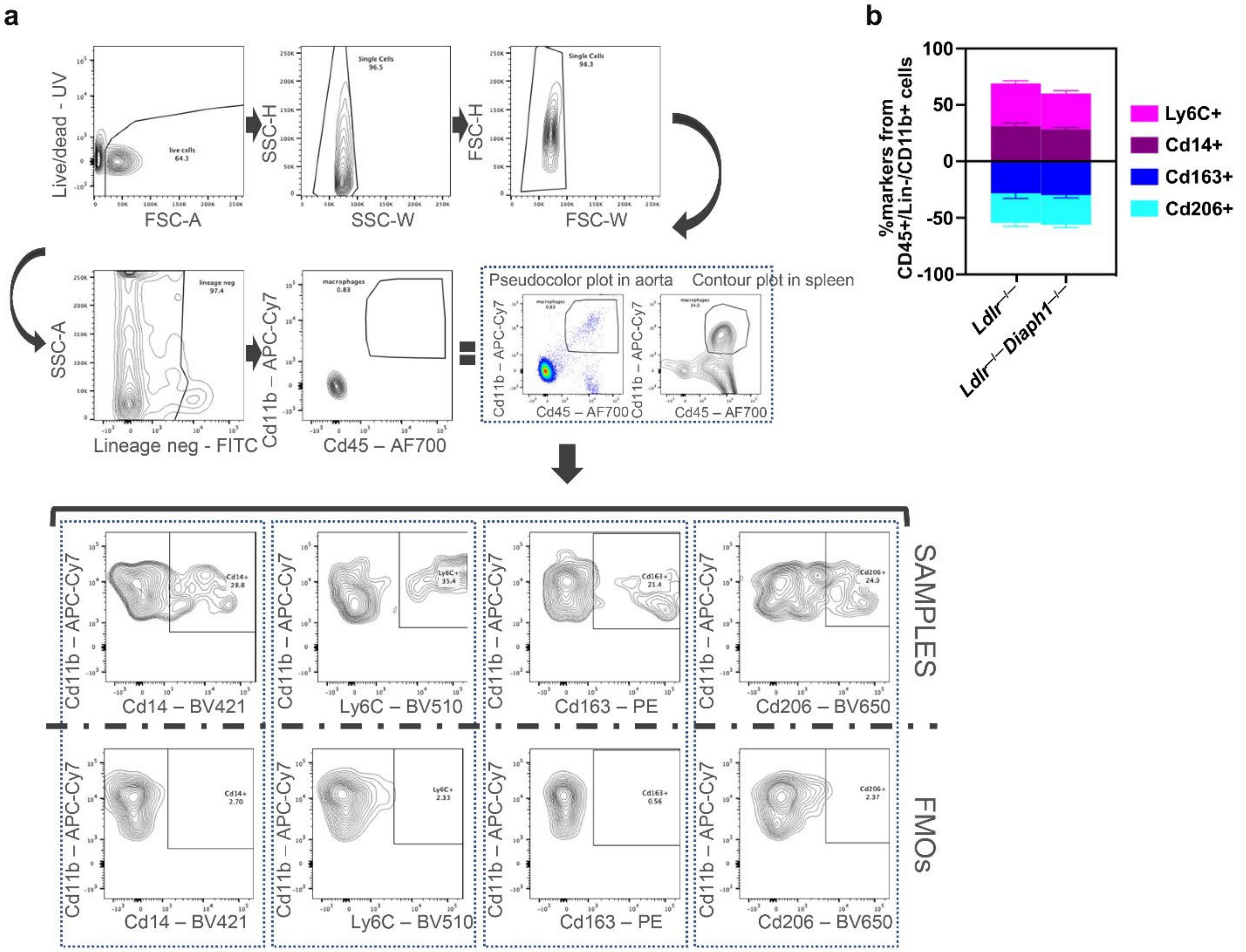

Supplementary Figure 3. Effect of deletion of *Diaph1* in *Ldlr*$^{-/-}$ mice on aortic arch macrophage characterization. *Ldlr*$^{-/-}$ and *Ldlr*$^{-/-}$*Diaph1*$^{-/-}$ male mice were fed WD for 16-18 weeks and aortic arches were perfused and retrieved for flow cytometry assay. **a,** gating strategy used for the analysis and characterization of macrophages isolated from aortic arches. Live single cells gated as CD45+ CD11b+ Lin- are used to delineate populations presumed to be macrophages. Additional gating was used to demonstrate the percentage of either CD14+, Ly6C+, CD163+ and CD206+. **b,** percentage of Ly6C+, CD14+, CD163+ and CD206+ macrophages in each genotype. The mean ± SEM is reported and in n=8 *Ldlr*$^{-/-}$ and n=10 *Ldlr*$^{-/-}$*Diaph1*$^{-/-}$ mice. Statistical analyses regarding testing for the normality of data followed by appropriate statistical analyses were described in Materials and Methods. According to these analyses, *P* values were determined by unpaired t test. **Supplementary Table 1** details all of the biological replicates for each condition and the associated statistical analysis.

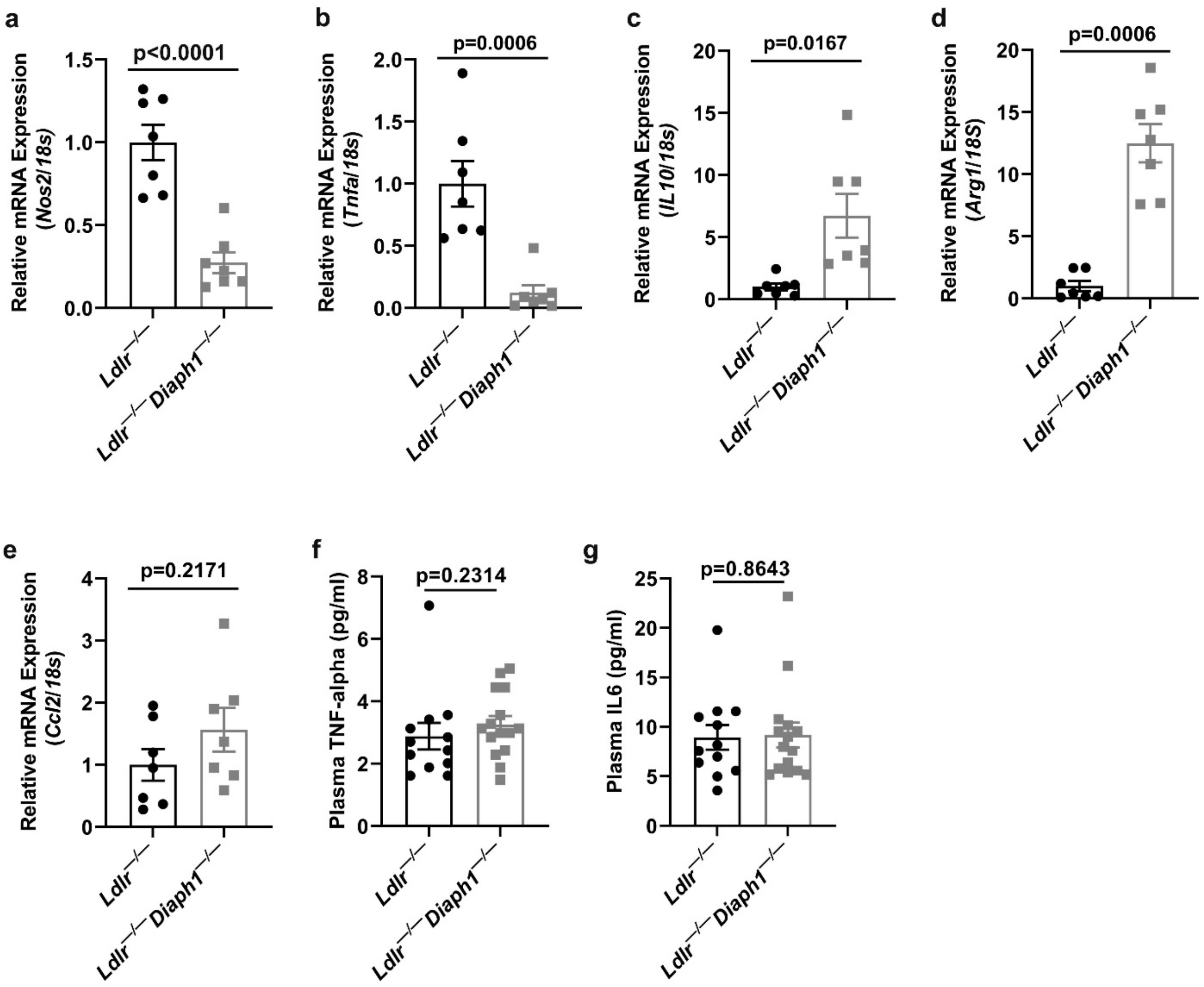

Supplementary Figure 4. Effect of deletion of *Diaph1* in male *Ldlr*$^{-/-}$ mice on aortic inflammation and plasma concentrations of TNF-alpha and IL6. *Ldlr* $^{-/-}$ and *Ldlr*$^{-/-}$ *Diaph1*$^{-/-}$ male mice were fed WD for 16 weeks. **a-e,** the expression of genes encoding inflammatory mediators was determined by RT-qPCR from whole aortas isolated from the indicated mice. **a,** *Nos2,* **b,** *Tnfa,* **c,** *Il10,* **d,** *Arg1,* **e,** *Ccl2.* **f,g,** ELISA for the detection of plasma concentrations of TNF-alpha (f) and IL6 (g) is shown. The mean ± SEM is reported. The number of independent mice/group is indicated in the figure as individual data points. Statistical analyses regarding testing for the normality of data followed by appropriate statistical analyses were described in Materials and Methods. *P* values were determined by unpaired t test and Wilcoxon rank-sum test depending on if the data passed the Shapiro-Wilk normality test.

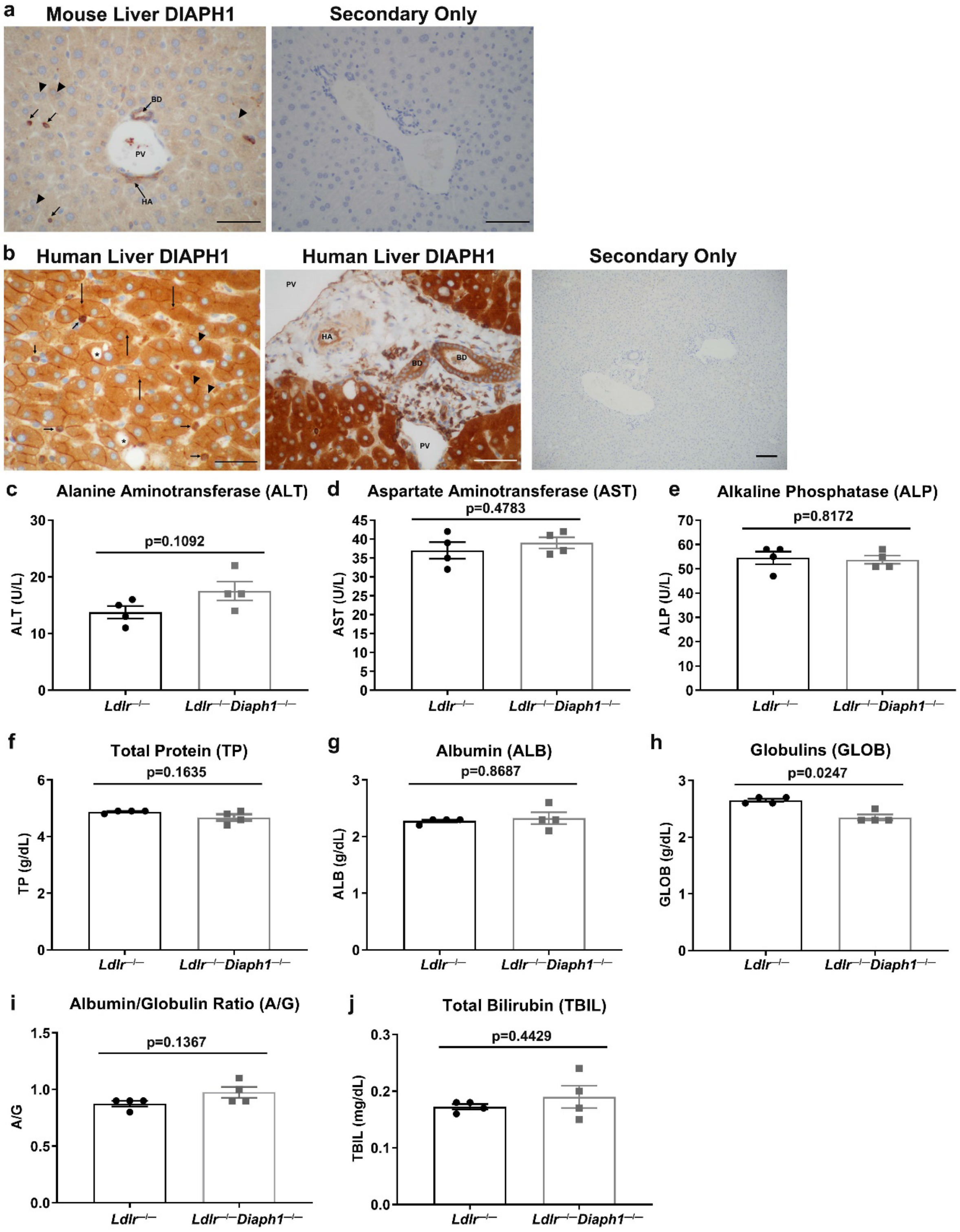

Supplementary Figure 5. DIAPH1 is expressed, at least in part, in hepatocytes of mouse and human liver, and the effect of deletion of *Diaph1* in *Ldlr*$^{-/-}$ mice on plasma analytes. a, mouse and b, human liver. Normal mouse (a) and normal human liver (b), the latter retrieved from a 52-year-old male subject, were subjected to fixation and immunohistochemistry using anti-

DIAPH1 IgG. The descriptions of the results are discussed in detail in the Results section. Abbreviations: BD, bile duct; PV, portal vein; and HA, hepatic artery. Scale bar: 50 µm. c-j, *Ldlr* $^{-/-}$ and *Ldlr* $^{-/-}$ *Diaph1* $^{-/-}$ male mice were fed WD for 16 weeks and plasma was retrieved for detection of: c, alanine aminotransferase (ALT) concentrations. d, aspartate aminotransferase (AST) concentrations. e, alkaline phosphatase (ALP) concentrations. f, total plasma protein (TP) concentrations. g, albumin (ALB) concentrations. h, globulin (GLOB) concentrations. i, albumin/globulins (A/G) ratio. j, total bilirubin (TBIL) concentrations. The mean ± SEM is reported. The number of independent mice/group is indicated in the figure as individual data points. Statistical analyses regarding testing for the normality of data followed by appropriate statistical analyses were described in Materials and Methods. *P* values were determined by unpaired t test and Wilcoxon rank-sum test depending on if the data passed the Shapiro-Wilk normality test.

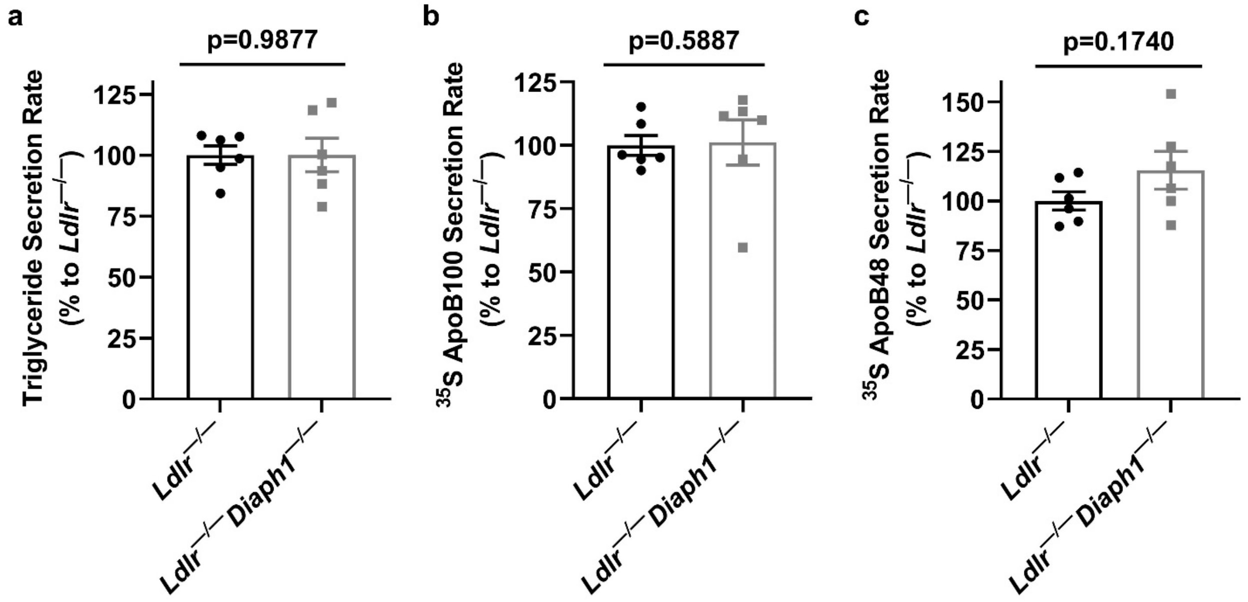

Supplementary Figure 6. Effect of deletion of *Diaph1* in *Ldlr*$^{-/-}$ mice on plasma triglyceride and lipoprotein secretion. *Ldlr* $^{-/-}$ and *Ldlr*$^{-/-}$ *Diaph1*$^{-/-}$ were fed chow diet. Secretion of **a,** triglyceride, **b,** apolipoprotein B100 (apoB100) and **c,** apolipoprotein B48 (apoB48) was determined *in vivo* two hours after injections of [^{35}S]-protein labeled in *Ldlr*$^{-/-}$ and *Ldlr*$^{-/-}$ *Diaph1*$^{-/-}$ mice. The mean ± SEM is reported. The number of independent mice/group is indicated in the figure as individual data points. Statistical analyses regarding testing for the normality of data followed by appropriate statistical analyses were described in Materials and Methods. *P* values were determined by unpaired t test and Wilcoxon rank-sum test depending on if the data passed the Shapiro-Wilk normality test.

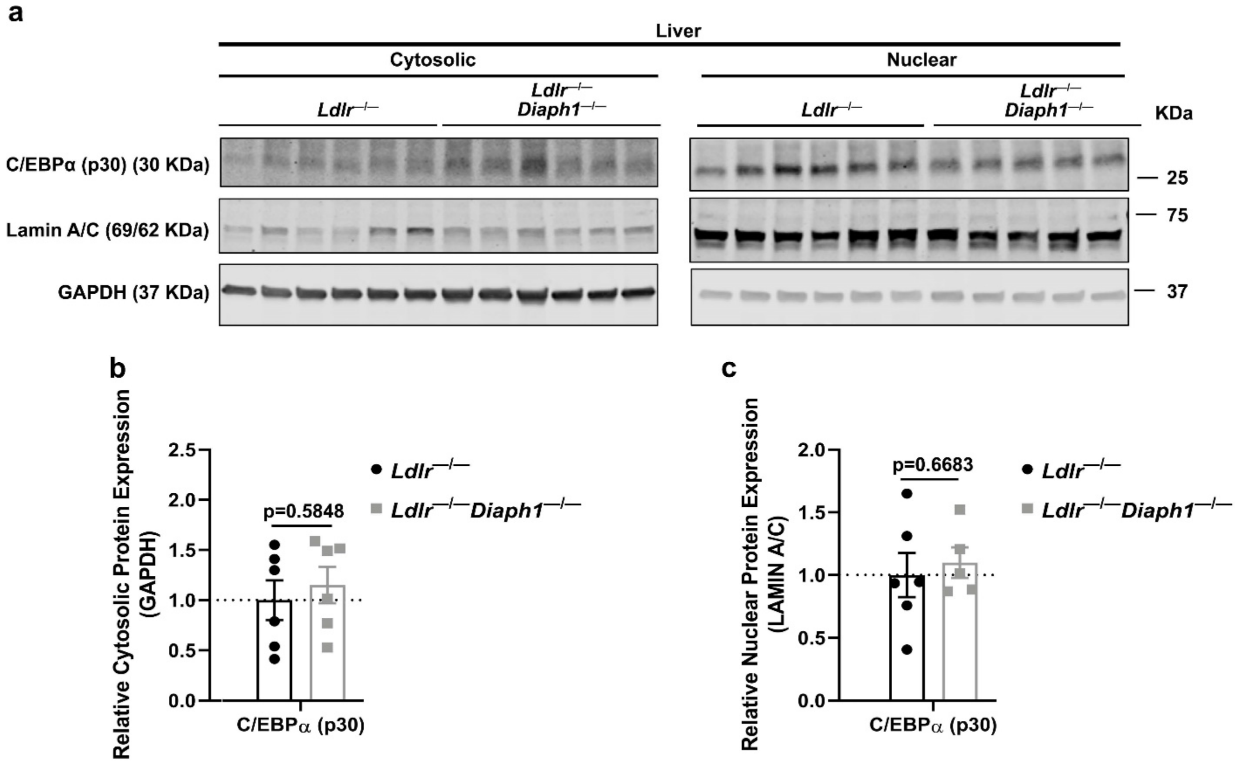

Supplementary Figure 7. Deletion of *Diaph1* in *Ldlr*$^{-/-}$ mice does not affect nuclear content of C/EBPα (p30) in liver. *Ldlr* $^{-/-}$ and *Ldlr*$^{-/-}$ *Diaph1*$^{-/-}$ male mice were fed WD for 16 weeks. **a,** Western blot for the detection of cytosolic and nuclear C/EBPα was performed on liver fractions isolated from the indicated mice. **b,** Quantification of cytosolic C/EBPα relative to GAPDH. **c,** Quantification of nuclear C/EBPα relative to Lamin A/C. The mean ± SEM is reported. The number of independent mice/group is indicated in the figure as individual data points. Statistical analyses regarding testing for the normality of data followed by appropriate statistical analyses were described in Materials and Methods. *P* values were determined by unpaired T test.

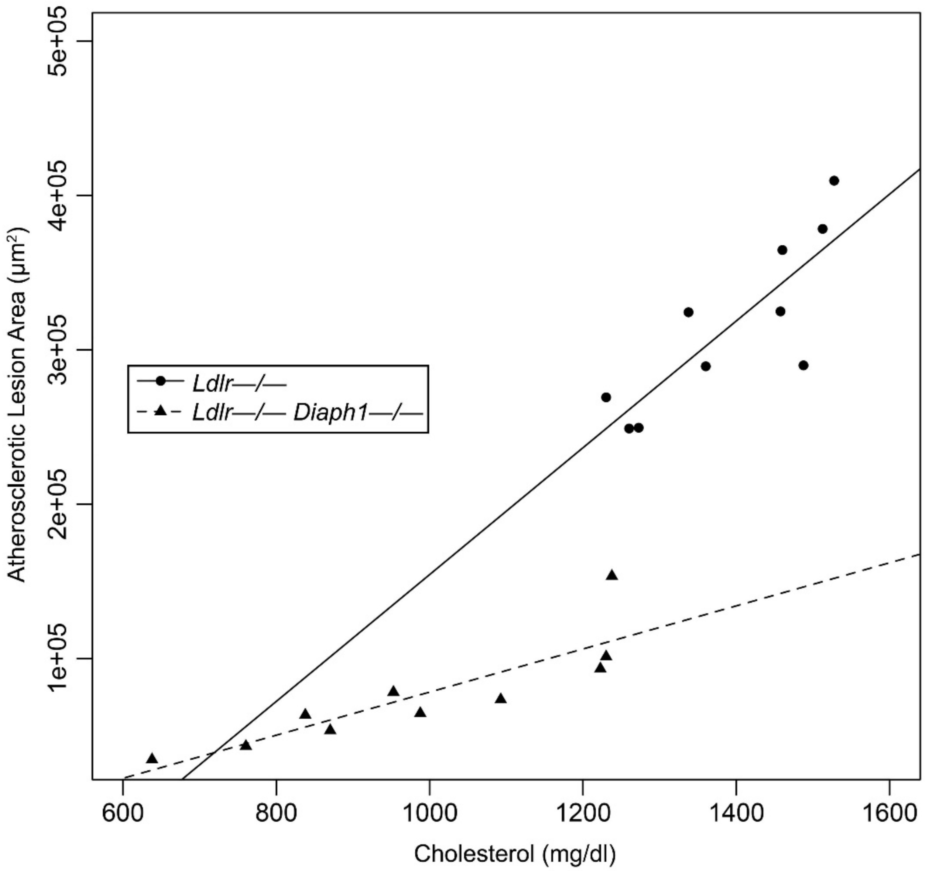

Supplementary Figure 8. **Dependence of atherosclerotic lesion area on the concentration of plasma cholesterol in male *Ldlr* —/— and *Ldlr*—/— *Diaph1*—/— male mice**. The graph is derived from data shown in Supplementary Table 9d.

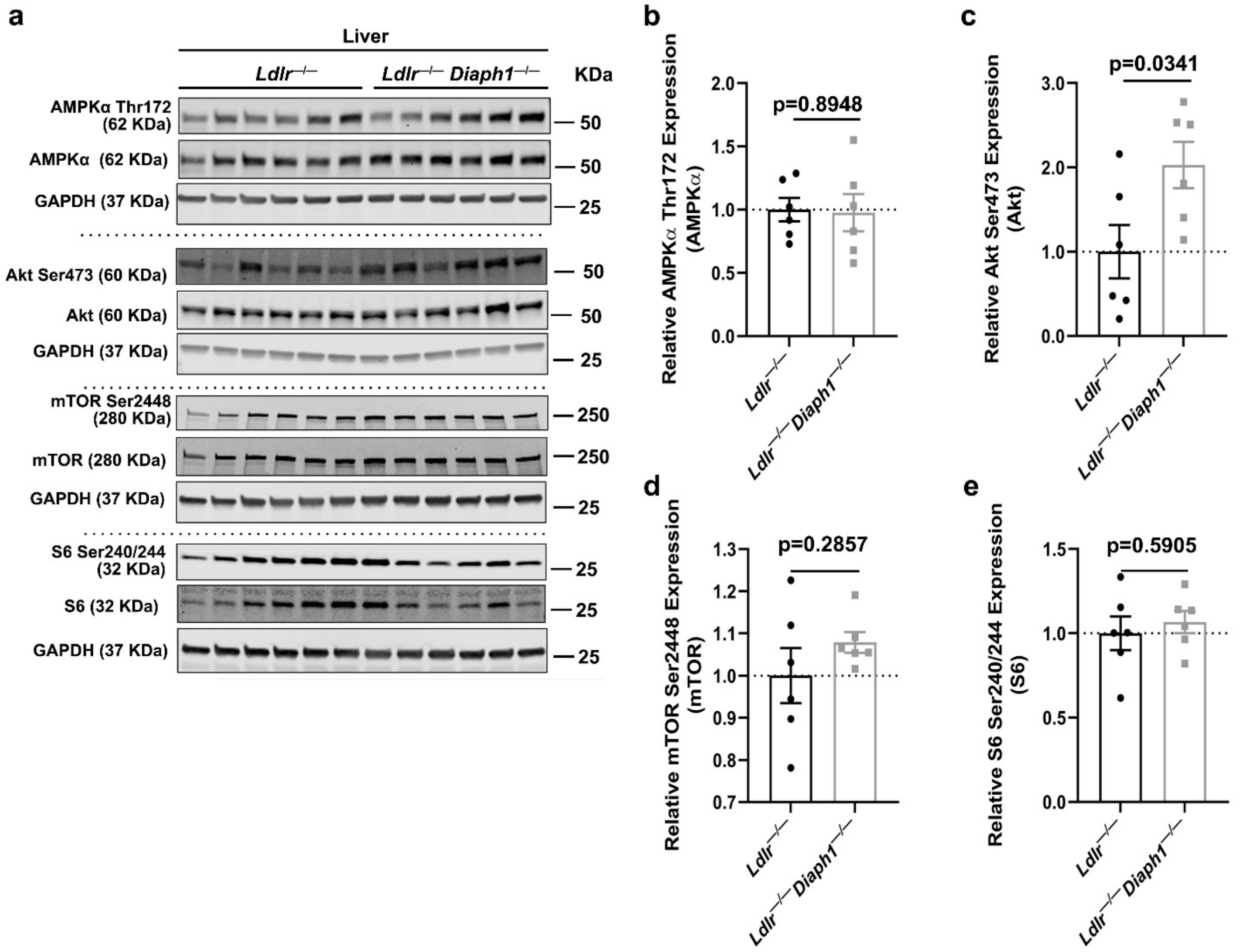

Supplementary Figure 9. Deletion of *Diaph1* in *Ldlr*$^{-/-}$ mice does not affect AMPKα, AKT or mTOR pathways in liver. *Ldlr*$^{-/-}$ and *Ldlr*$^{-/-}$ *Diaph1*$^{-/-}$ male mice were fed WD for 16 weeks. **a,** Western blot for the detection of phosphorylated (Thr172) AMPKα, AMPKα, phosphorylated (Ser473) AKT, AKT, phosphorylated (Ser2448) mTOR, mTOR, phosphorylated (Ser240/244) S6 and S6 performed on total liver lysates from the indicated mice. **b,** Quantification of phosphorylated (Thr172) AMPKα relative to total AMPKα. **c,** Quantification of phosphorylated (Ser473) AKT relative to total AKT. **d,** Quantification of phosphorylated (Ser2448) mTOR relative to total mTOR. **e,** Quantification of phosphorylated (Ser240/244) S6 relative to total S6. The mean ± SEM is reported. The number of independent mice/group is indicated in the figure as individual data points. Statistical analyses regarding testing for the normality of data followed by appropriate statistical analyses were described in Materials and Methods. *P* values were determined by unpaired T test.

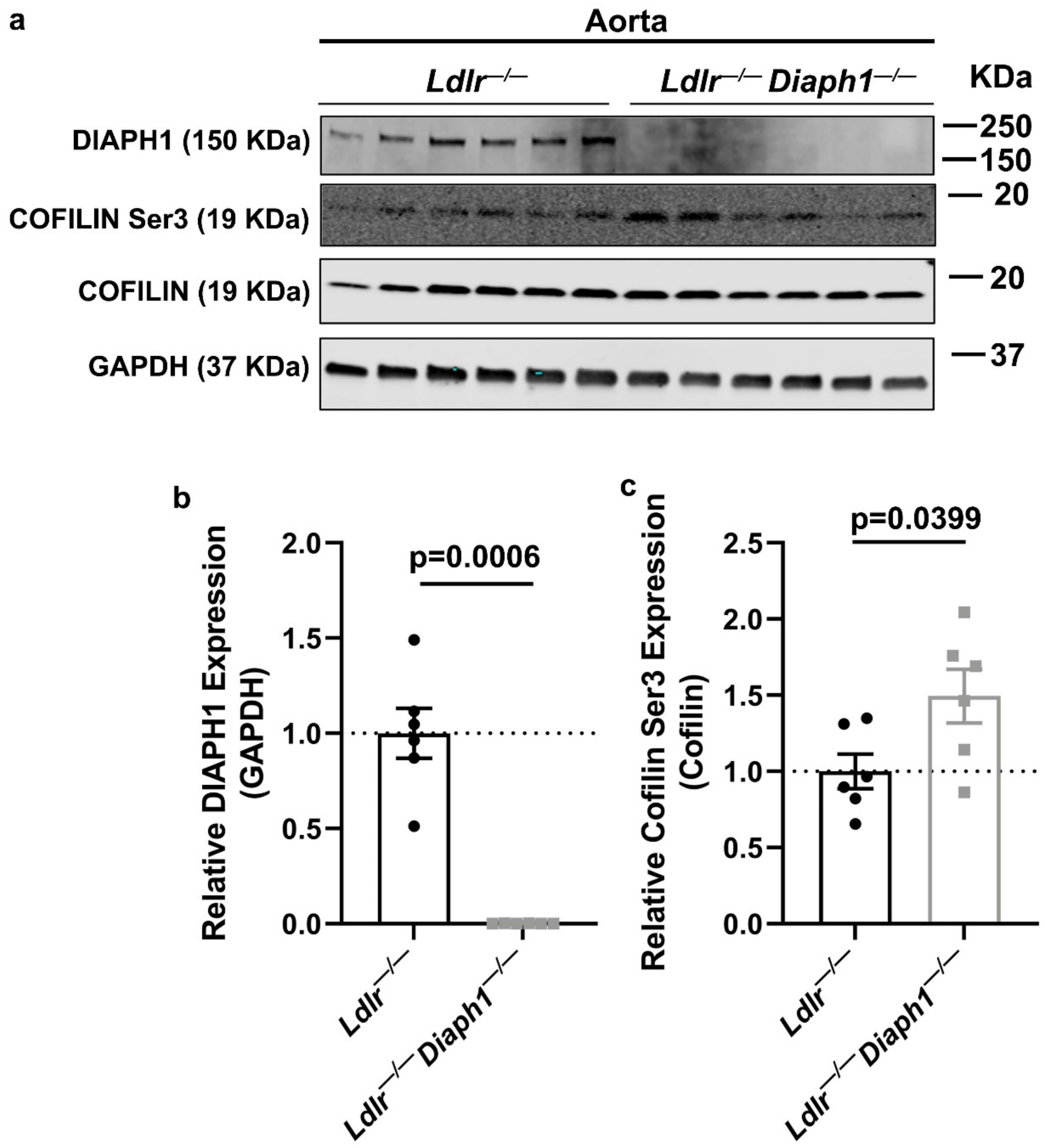

Supplementary Figure 10. Deletion of *Diaph1* in *Ldlr*$^{-/-}$ mice increases phosphorylated (Ser3) Cofilin/total Cofilin in aortas. *Ldlr* $^{-/-}$ and *Ldlr*$^{-/-}$ *Diaph1*$^{-/-}$ male mice were fed WD for 16 weeks. **a,** Western blot for the detection of DIAPH1, phosphorylated (Ser3) Cofilin and total Cofilin was performed on total lysates from aortas isolated from the indicated mice. **b,** Quantification of DIAPH1 relative to GAPDH. **c,** Quantification of phosphorylated (Ser3) Cofilin relative to total Cofilin. The mean ± SEM is reported. The number of independent mice/group is indicated in the figure as individual data points. Statistical analyses regarding testing for the normality of data followed by appropriate statistical analyses were described in Materials and Methods. *P* values were determined by unpaired T test.

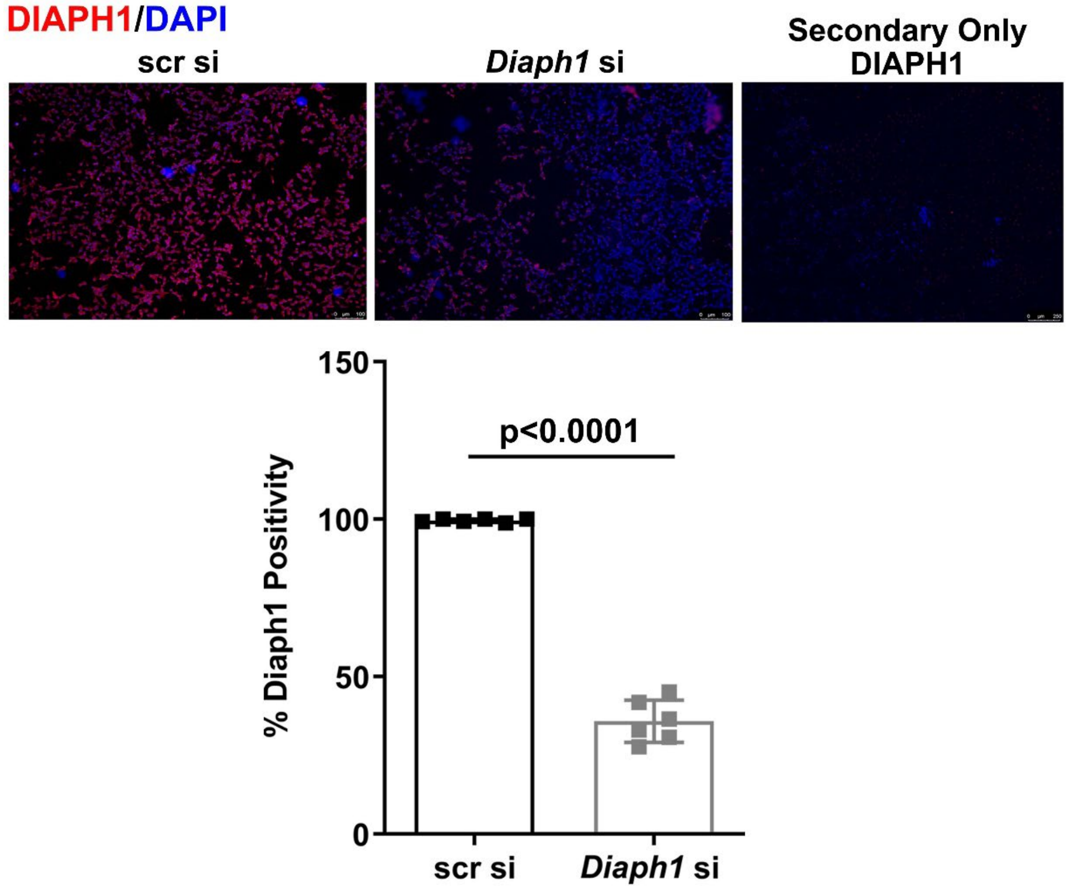

Supplementary Figure 11. Silencing of *Diaph1* in Hepa 1-6 cells. Representative immunofluorescence staining and quantification of DIAPH1 in murine Hepa1-6 after *Diaph1* or scramble control siRNA treatments. Scale bar: 250 µm. The mean ± SEM is reported. The number of independent mice/group is indicated in the figure as individual data points. Statistical analyses regarding testing for the normality of data followed by appropriate statistical analyses were described in Materials and Methods. *P* values were determined by unpaired T test or Wilcoxon rank-sum test depending if data passed the Shapiro-Wilk normality test.

Supplementary Tables

Supplementary Table 1. Effect of deletion of *Diaph1* in male *Ldlr*$^{-/-}$ mice on aortic arch macrophage characterization: flow cytometry studies

	Ldlr$^{-/-}$ (% markers/total macrophages)				*Ldlr*$^{-/-}$*Diaph1*$^{-/-}$ (% markers/total macrophages)			
	CD14+	Ly6C+	CD163+	CD206+	CD14+	Ly6C+	CD163+	CD206+
	19.70	37.55	13.75	20.07	21.74	37.89	18.63	23.60
	24.49	38.78	7.14	12.24	28.38	31.08	23.65	27.70
	30.21	28.13	21.88	31.25	30.16	28.57	25.40	28.57
	26.47	28.47	32.90	23.32	25.58	24.42	22.09	34.88
	36.97	45.66	30.96	24.72	32.76	36.64	46.12	28.02
	28.43	36.78	35.79	24.45	30.99	25.93	32.31	16.04
	44.00	44.80	46.40	40.80	21.30	44.38	28.40	16.57
	39.29	41.07	36.31	33.33	36.25	26.76	31.63	17.27
					22.30	19.42	36.69	33.81
					35.48	41.13	33.06	37.10
Mean	31.1942	37.6530	28.1411	26.2746	28.4946	31.6227	29.7988	26.3574
SEM	2.8995	2.3299	4.5832	3.0918	1.7652	2.5468	2.5329	2.4616
N	8	8	8	8	10	10	10	10

Unpaired t test

	CD14+	Ly6C+	CD163+	CD206+
pvalue	0.4184	0.1072	0.7429	0.9833

Supplementary Table 2. Body Weight and Biochemical Parameters in Female *Ldlr*$^{-/-}$ vs. *Ldlr*$^{-/-}$*Diaph1*$^{-/-}$ Mice on WD for 16 Weeks

	**Mouse Group**	
**Parameter**	*Ldlr*$^{-/-}$	*Ldlr*$^{-/-}$ *Diaph1*$^{-/-}$
Body Mass (g)	20.0 ± 0.9 (N=8)	21.2 ± 0.7 (N=9); p= 0.3070
Cholesterol (mg/dL)	1094.0 ± 84.1 (N=8)	604.3 ± 21.7 (N=9); p<0.0001
Triglyceride (mg/dL)	116.9 ± 17.0 (N=8)	99.9 ± 10.6 (N=9); p=0.3690
Glucose (mg/dL)	131.5 ± 11.1 (N=8)	128.1 ± 6.3 (N=9); p=0.7880

Values represent Mean±SEM.

Supplementary Table 3. Significant Signaling Pathway Impact Analysis (KEGG) Pathways

Pathway	Size/Overlap	*P*-value^	FDR^
Renin-angiotensin system	14/1	0.002	0.47
Systemic lupus erythematosus	44/6	0.006	0.74
Neuroactive ligand-receptor interaction	67/3	0.017	0.83
Propanoate metabolism	27/4	0.018	0.83
Peroxisome	76/7	0.026	0.83
**Glycerophospholipid metabolism**	67/7	0.042	0.83
Porphyrin and chlorophyll metabolism	35/4	0.043	0.83
Sulfur metabolism	9/2	0.044	0.83

^All p values and FDR values above refer to those computed for the KEGG pathway analysis. All of the genes included within the above pathways fulfill the criteria: p<0.05 and FDR<0.05.

Supplementary Table 4. Reactome "Metabolism" (top hit) pathway: 70 differentially expressed genes

Gene Symbol	Gene Name	Gene function (general)
Tat	tyrosine aminotransferase	tyrosine metabolism
Fmo2	flavin-containing Dimethylaniline monoxygenase2	alkylamine metabolism
Aadat	aminoadipate aminotransferase	amino acid metabolism
Hprt	Hypoxanthine Phosphoribosyltransferase 1	generation of purine nucleotides
Lypla1	lysophospholipase 1	**fatty acid metabolism**
Cpox	coproporphyrinogenIII oxidase	heme metabolism
Adhd3	Abhydrolase domain containing 3	(possible) **phospholipase 1 activity**
Amacr	alpha methyl acyl co-A racemase	**fatty acid metabolism**
Acer2	alkaline ceramidase 2	**ceramide metabolism**
Lpin1	phosphatidate phosphatase 1	**triglyceride synthesis**
Nudt16	nudix hydrolase 16	nucleotide metabolism
Gclm	glutamate-cysteine ligase Modifier subunit	glutathione synthesis
Rrm1	ribonucleotide reductase catalytic Subunit M1	nucleotide metabolism
Mtmr7	myotubularin related protein 7	tyrosine dual specificity phosphatase
Pla1a	phospholipase A1 member A	**fatty acid metabolism**
Ugt2b38	UDP glucuronosyltransferase Family 2 member b28	steroid/drug metabolism
Car1	carbonic anhydrase 1	carbonate dehydratase activity
Sat1	spermidine/spermine N1-Acetyltransferase1	polyamine metabolism
Hscb	HscB mitochondrial iron-sulfur Cluster cochaperone	mitochondrial electron transport metabolism
Serinc1	serine incorporator 1	serine and **lipid metabolism**
Dct	dopachrome tautomerase	tyrosine metabolism
Prkacb	protein kinase CAMP-activated Catalytic subunit beta	serine/threonine protein kinase
Vapa	VAMP associated protein A	vesicle trafficking, migration
Chka	choline kinase alpha	ethanolamine metabolism
Akr1c6	aldo-keto reductase C6	aldo-keto reductase metabolism
Fabp7	fatty acid binding protein 7	**fatty acid metabolism**
Pnpla8	patatin like phospholipase Domain containing 8	**fatty acid metabolism**

Mfsd2a	major facilitator superfamily Domain containing 2a	**lysophosphatidylcholine metabolism**
Pcca	propionyl CoA carboxylase Subunit alpha	**fatty acid** and branched chain amino acid metabolism
Ppt1	palmitoyl protein thioesterase 1	**lipid metabolism**
Idh3a	isocitrate dehydrogenase	tricarboxylic acid metabolism
Otc	ornithine carbamoyltransferase	ornithine metabolism
Echs1	enoyl coA hydratase short chain 1	**fatty acid oxidation**
Ugt3a2	UDP glycosyltransferase family 3 Member A2	glycosyltransferase metabolism
Calm2	calmodulin 2	protein kinases and phosphatases, calcium family
Nudt7	nudix hydrolase 7	nucleotide metabolism
Tpk1	thiamine pyrophosphokinase 1	thiamine metabolism
Gpat2	glycerol-3-phosphate acyltransferase 2	**glycerolipid biosynthesis**
Pdpr	pyruvate dehydrogenase phosphatase Regulatory subunit	**fatty acid synthesis,** TCA cycle
Acacb	acetyl-coA-carboxylase beta	**fatty acid synthesis**
Ip6k2	inositol hexakisphosphate kinase 2	inositol phosphate metabolism
Bbox1	gamma butyrobetaine hydroxylase1	**fatty acid metabolism**
Rapgef4	rap guanine nucleotide exchange Factor 4	GPCR signaling
Alox5ap	arachidonate 5-lipoxygenase Activating protein	leukotriene synthesis
Lpin2	lipin 2	triglyceride metabolism
Mat2b	methionine adenosyltransferase 2b	methione metabolism
Serinc5	serine incorporator 5	serine and glycosphinolipid metabolism
Pfkfb3	6-phospho-fructo-2-kinase/ Fructose-2,6-biphosphatase3	glycolysis
Phyh	Phytanoyl-CoA 2-Hydroxylase	**fatty acid metabolism**
Slc35d1	soluble carrier family 35 member D1	glucuronidation pathway
Acaca	Acetyl-CoA Carboxylase Alpha	**fatty acid synthesis**
Abcd4	ATP Binding Cassette Subfamily D Member 4	**fatty acid transport**
Cryl1	crystallin lambda 1	glucose metabolism
Akr1c20	Aldo-Keto Reductase Family 1 Member C20	aldo-ketoreductase metabolism
Dck	Deoxycytidine Kinase	nucleotide metabolism
L2hgdh	L-2-Hydroxyglutarate Dehydrogenase	pyruvate and TCA metabolism
Acadsb	Short/branched chain	**fatty acid**/branched chain amino acid

	acyl-CoA dehydrogenase	metabolism
Cyp26b	cytochrome p450 family 26 Subfamily B member 1	drug metabolism
Sult2a8	sulfotransferase family 2A, Dehydroepiandrosterone	steroid metabolism
Rab5a	RAB5A, Member RAS Oncogene Family	GPCR signaling
Bpnt1	bisphosphate 3-prime- Nucleotidase	nucleotide metabolism
Ugt2a3	UDP Glucuronosyltransferase Family 2 Member A3	drug metabolism
Slc35b2	soluble carrier family 35 member B2	sulfation processes
Plcd3	1-Phosphatidylinositol-4,5-bisphosphate phosphodiesterase delta-3	**fatty acid metabolism**
Fmod	fibromodulin	collagen metabolism
Hmox1	heme oxygenase	heme metabolism
Lum	lumican	collagen metabolism
Impad1	inositol monophosphatase domain containing 1	inositol metabolism
Akr1d1	Aldo-Keto Reductase Family 1 Member D1	bile acid and steroid metabolism
Rfk	riboflavin kinase	riboflavin metabolism

Supplementary Table 5. Significant Reactome Pathways

Pathway	Size/Overlap	*P*-value^	FDR^
**Metabolism**	1810/70	0.000002	0.004
Phase II Conjugation of compounds	105/10	0.0001	0.11
**Protein Localization**	73/8	0.0003	0.13
**Synthesis of phosphatidylcholine**	27/5	0.0003	0.13
**Synthesis of bile acids and bile salts via 24-hydroxycholesterol**	19/4	0.0008	0.25
**Transport of nucleotide sugars**	9/3	0.0009	0.25
**Triglyceride metabolism**	23/4	0.002	0.31
Biotin transport and metabolism	11/3	0.002	0.31
**Synthesis of phosphatidylethanolamine**	11/3	0.002	0.31
Glucoronidation	24/4	0.002	0.33
**Triglyceride biosynthesis**	12/3	0.002	0.34
Regulation of complement cascade	42/5	0.003	0.34
Cytosolic sulfonation of small molecules	26/4	0.003	0.34
**Peroxisomal protein import**	65/6	0.004	0.43
**Synthesis of bile acids and bile salts**	19/4	0.0008	0.25
**via 7alpha-hydroxycholesterol**	29/4	0.004	0.45
Depolymerization of the nuclear lamina	15/3	0.004	0.45
Complement cascade	48/5	0.005	0.45
**Fatty acid metabolism**	196/11	0.006	0.51
**Glycerophospholipid biosynthesis**	119/8	0.006	0.52
**Metabolism of Lipids**	652/25	0.008	0.65
**ChREBP activates metabolic gene expression**	7/2	0.01	0.67
Pre-NOTCH processing in Golgi	7/2	0.01	0.67
TP53 regulates transcription of genes involved in cytochrome c release	7/2	0.01	0.67
TP53 regulates transcription of genes involved in G2 cell cycle arrest	7/2	0.01	0.67
**Synthesis of bile acids and bile salts via 27-hydroxycholesterol**	20/3	0.01	0.67
**Synthesis of bile acids and bile salts**	39/4	0.01	0.73
**Phospholipid metabolism**	191/10	0.01	0.73
Biological oxidations	251/12	0.01	0.73
Mitochondrial protein import	8/2	0.01	0.73
Terminal pathway of complement	8/2	0.01	0.73

**Transport of vitamins, nucleosides and related molecules**	**41/4**	**0.01**	**0.75**
Neutrophil degranulation	514/20	0.01	0.75
Serine biosynthesis	9/2	0.02	0.81
Pre-NOTCH expression and processing	9/2	0.02	0.82
Receptor mediated mitophagy	11/2	0.03	1
**Bile acid and bile salt metabolism**	51/4	0.03	1
Keratan sulfate degradation	12/2	0.03	1
Metabolism of vitamins and cofactors	189/9	0.03	1
**Peroxisomal lipid metabolism**	30/3	0.03	1
eNOS activation	13/2	0.03	1
Signaling by retinoic acid	56/4	0.04	1
Purine salvage	14/2	0.04	1
Rap1 signaling	14/2	0.04	1
**Transport of small molecules**	686/23	0.04	1
Negative regulation of NOTCH4 signaling	2/1	0.045	1
Transfer of LPS from LBP carrier to CD14	2/1	0.045	1
NOTCH4 activation and transmission of signal to the nucleus	2/1	0.045	1
**Import of palmitoyl CoA into the mitochondrial matrix**	15/2	0.045	1
TP53 regulates transcription of cell death genes	15/2	0.045	1
RA biosynthesis pathway	35/3	0.046	1

^All p values and FDR values above refer to those computed for the Reactome pathway analysis. All of the genes included within the above Reactome pathways fulfill the criteria: $p<0.05$ and $FDR<0.05$.

Categories highlighted in BOLD reflect examples of pathways related to lipid metabolism, protein localization and transport and actin cytoskeleton.

Supplementary Table 6. Significant Gene Ontology Biological Process pathways

Symbol	Pathway	Size/Overlap	*P*-value
GO:0002084	Protein depalmitoylation	9/4	0.000
GO:0070268	Cornification	2/2	0.001
GO:0090285	Negative regulation of protein glycosylation in Golgi	2/2	0.001
GO:006103	2-oxoglutarate metabolic process	16/4	0.003
GO:0090481	Pyrimidine nucleotide-sugar Transmembrane transport	9/3	0.004
GO:1904219	Positive regulation of CDP-diacylglycerol-Serine O-phosphatidlyltransferase activity	3/2	0.004
GO:1904222	Positive regulation of serine C-palmitoyl-transferase activity	3/2	0.004
GO:0007625	Grooming behavior	10/3	0.005
GO:0039689	Negative stranded viral RNA replication	4/2	0.008
GO:0043305	Negative regulation of mast cell degranulation	4/2	0.008
GO:0002043	Blood vessel endothelial cell proliferation involved in sprouting angiogenesis	12/3	0.009
GO:0043484	Regulation of RNA splicing	112/10	0.010
GO:0035020	**Regulation of Rac protein signal transduction**	**13/3**	**0.011**
GO:0010269	Response to selenium ion	5/2	0.013
GO:0015739	Sialic acid transport	5/2	0.013
GO:0042997	Negative regulation of Golgi to plasma membrane protein transport	5/2	0.013
GO:0045919	Positive regulation of cytolysis	5/2	0.013
GO:0033628	**Regulation of cell adhesion by integrin**	27/4	0.018
GO:0031214	Biomineral tissue development	88/8	0.018
GO:0014883	Transition between fast and slow fiber	6/2	0.019
GO:0045964	Positive regulation of dopamine metabolic process	6/2	0.019
GO:0070973	Protein localization to endoplasmic reticulum exit site	6/2	0.019
GO:0075044	Autophagy of host cells involved in interaction with symbiont	6/2	0.019
GO:2000302	Positive regulation of synaptic vesicle exocytosis	6/2	0.019
GO:0006625	Protein targeting to peroxisome	16/3	0.021
GO:0048169	Regulation of long-term neuronal synaptic plasticity	16/3	0.021
GO:0006817	Phosphate ion transport	17/3	0.024
GO:0022011	Myelination in peripheral nervous system	17/3	0.024

GO:0008300	Isoprenoid catabolic process	7/2	0.026
GO:0098887	Neurotransmitter receptor transport, endosome to postsynaptic membrane	7/2	0.026
GO:0008630	Intrinsic apoptotic signaling pathway in response to DNA damage	96/8	0.028
GO:0021846	Cell proliferation in forebrain	18/3	0.029
GO:0045056	Transcytosis	18/3	0.029
GO:0001503	Ossification	212/14	0.030
GO:0006958	Complement activation, classical Pathway	32/4	0.031
GO:0016226	Iron-sulfur cluster assembly	19/3	0.033
GO:0060349	Bone morphogenesis	64/6	0.033
GO:0016559	Peroxisome fission	8/2	0.034
GO:0043653	Mitochondrial fragmentation involved in apoptotic process	8/2	0.034
GO:0044597	Daunorubicin metabolic process	8/2	0.034
GO0044598	Doxorubicin metabolic process	8/2	0.034
GO:0051409	Response to nitrosative stress	8/2	0.034
GO:0090160	**Golgi to lysosomes transport**	8/2	0.034
GO:0046854	Phosphatidylinositol phosphorylation	33/4	0.034
GO:0050873	Brown fat cell differentiation	33/4	0.034
GO:0009062	**Fatty acid catabolic process**	84/8	0.036
GO:0009404	Toxic metabolic process	20/3	0.038
GO:0001519	Peptide Amidation	1/1	0.038
GO:0001869	Negative regulation of complement activation, lectin pathway	1/1	0.038
GO:0002037	Negative regulation of L-glutamate import across plasma membrane	1/1	0.038
GO:0002100	tRNA wobble adenosine to inosine editing	1/1	0.038
GO:0006178	Guanine salvage	1/1	0.038
GO:0006580	Ethanolamine metabolic process	1/1	0.038
GO:0006583	Melanin biosynthetic process from tyrosine	1/1	0.038
GO:0006593	Ornithine catabolic process	1/1	0.038
GO:0007223	Wnt signaling pathway, calcium modulating Pathway	1/1	0.038
GO:0008355	Olfactory learning	1/1	0.038
GO:0009229	thiamine diphosphate biosynthetic process	1/1	0.038
GO:0009231	Riboflavin biosynthetic process	1/1	0.038
GO:0009398	FMN biosynthetic process	1/1	0.038
GO:0010768	FMN biosynthetic process	1/1	0.038
GO:0010768	Negative regulation of transcription from RNA polymerase II promoter in response to UV-induced DNA damage	1/1	0.038
GO:0015862	Uridine transport	1/1	0.038
GO:0015938	Coenzyme A catabolic process	1/1	0.038

GO:0018032	Protein amidation	1/1	0.038
GO:0019372	**Lipoxygenase pathway**	1/1	0.038
GO:0019606	2-oxobutyrate catabolic process	1/1	0.038
GO:0021571	rhombomere 5 development	1/1	0.038
GO:0021572	rhombomere 6 development	1/1	0.038
GO:0021599	abducens nerve formation	1/1	0.038
GO:0021633	optic nerve structural organization	1/1	0.038
GO:0021933	radial glia guided migration of cerebellar granule cell	1/1	0.038
GO:0031460	glycine betaine transport	1/1	0.038
GO:0032263	GMP salvage	1/1	0.038
GO:0033373	Maintenance of protease location in mast cell secretory granule	1/1	0.038
GO:0033382	Maintenance of granzyme B location in T cell secretory granule	1/1	0.038
GO:0035229	Positive regulation of glutamate-cysteine ligase activity	1/1	0.038
GO:0025284	Brain segmentation	1/1	0.038
GO:0035750	Protein localization to myelin sheath abaxonal region	1/1	0.038
GO:005863	dITP catabolic process	1/1	0.038
GO:0036372	Opsin transport	1/1	0.038
GO:0042335	Cuticle development	1/1	0.038
GO:0042701	Progesterone secretion	1/1	0.038
GO:0043585	Nose morphogenesis	1/1	0.038
GO:0045751	Negative regulation of Toll signaling pathway	1/1	0.038
GO:0046963	3’-phosphoadenosine 5’-phosphosulfate transport	1/1	0.038
GO:0048388	Endosomal lumen acidification	1/1	0.038
GO:0051977	**Lysophospholipid transport**	1/1	0.038
GO:0060035	Notochord cell development	1/1	0.038
GO:0060112	Generation of ovulation cycle rhythm	1/1	0.038
GO:0061301	Cerebellum vasculature morphogenesis	1/1	0.038
GO:0061843	Sertoli cell barrier remodeling	1/1	0.038
GO:0070267	Oncosis	1/1	0.038
GO:0071240	Cellular response to food	1/1	0.038
GO:0071395	Cellular response to jasmonic acid stimulus	1/1	0.038
GO:0071420	Cellular response to histamine	1/1	0.038
GO:0090119	**Vesicle mediated cholesterol transport**	1/1	**0.038**
GO:0090326	**Positive regulation of locomotion involved in locomotory behavior**	**1/1**	**0.038**
GO:0097272	Ammonia homeostasis	1/1	0.038
GO:0099564	Modification of synaptic structure, modulating synaptic transmission	1/1	0.038
GO:0120061	Negative regulation of gastric emptying	1/1	0.038

GO:1901329	Regulation of odontoblast differentiation	1/1	0.038
GO:1901398	Regulation of transforming growth factor beta3 activation	1/1	0.038
GO:1901639	XDP catabolic process	1/1	0.038
GO:1901835	Positive regulation of deadenylation-independent decapping of nuclear transcribed mRNA	1/1	0.038
GO:1902340	Negative regulation of chromosome condensation	1/1	0.038
GO:1902725	Negative regulation of satellite cell differentiation	1/1	0.038
GO:1902823	**Negative regulation of late endosome to lysosome transport**	**1/1**	**0.038**
GO:1903996	Negative regulation of non-membrane Spanning protein kinase activity	1/1	0.038
GO:1903999	Negative regulation of eating behavior	1/1	0.038
GO:1904060	Negative regulation of locomotor rhythm	1/1	0.038
GO:1904246	Negative regulation of polynucleotide adenyltransferase activity	1/1	0.038
GO:1904750	**Negative regulation of protein localization to nucleolus**	**1/1**	**0.038**
GO:1905396	Cellular response to flavonoid	1/1	0.038
GO:1905524	Negative regulation of protein autoubiquitination	1/1	0.038
GO:1990511	piRNA biosynthetic process	1/1	0.038
GO:2001037	Positive regulation of tongue muscle cell differentiation	1/1	0.038
GO:2001255	Positive regulation of histone H3-K36 trimethylation	1/1	0.038
GO:2001295	Malonyl-CoA biosynthetic process	1/1	0.038
GO:0031644	Regulation of neurological system process	84/7	0.039
GO:0032233	**Positive regulation of actin filament bundle assembly**	**50/5**	**0.039**
GO:0050710	Negative regulation of cytokine secretion	35/5	0.042
GO:0009064	Glutamine family amino acid metabolic process	51/5	0.042
GO:0030903	Notochord development	10/3	0.043
GO:0031100	Animal organ regeneration	21/3	0.043
GO:0002739	Regulation of cytokine secretion involved	9/2	0.043
GO:0042403	Thyroid hormone metabolic process	9/2	0.043
GO:0051451	Myoblast migration	9/2	0.043
GO:0070278	Extracellular matrix constituent secretion	9/2	0.043
GO:1902950	Regulation of dendritic spine maintenance	9/2	0.043

Categories highlighted in BOLD reflect examples of pathways related to lipid metabolism, protein localization and transport and actin cytoskeleton.

Supplementary Table 7. Body Weight and Biochemical Parameters in Male *Ldlr*$^{-/-}$ vs. *Ldlr*$^{-/-}$*Diaph1*$^{-/-}$ Mice on WD for 16 Weeks

	**Mouse Group**	
**Parameter**	*Ldlr*$^{-/-}$	*Ldlr*$^{-/-}$ *Diaph1*$^{-/-}$
Body Weight (g)	27.7 ± 0.2 (N=22)	26.2 ± 0.2 (N=22); p<0.0001
Glucose (mg/dL)	162.5 ± 3.2 (N=22)	157.1± 2.5 (N=22); p=0.2000
Insulin (µg/L)	0.85 ± 0.4 (N=9)	0.39 ± 0.05 (N=9); p=0.5360
Glucagon (µg/L)	101.8 ± 15 (N=10)	85.8 ± 22 (N=11); p=0.1920
Insulin/Glucagon Ratio	0.0075 ± 0.002 (N=9)	0.0053 ± 5E-04 (N=9); p=0.7960
HOMA-IR Index	8.3 ± 3.3 (N=9)	3.5 ± 0.5 (N=9); p=0.2580

Values represent Mean±SEM.

Supplementary Table 8. Correlation Analysis of the Atherosclerotic Lesion Area and Macrophage/Neutral Lipid Content vs. lipid and other metabolic factors in Male *Ldlr*$^{-/-}$ vs. *Ldlr*$^{-/-}$*Diaph1*$^{-/-}$ Mice on WD for 16 Weeks

Genotype/Variable		Correlation Coefficient/P-value		*Ldlr*$^{-/-}$ *Diaph1*$^{-/-}$ vs. *Ldlr*$^{-/-}$	
A).	Atherosclerotic Lesion Area (H&E) vs. Neutral Lipid Content (ORO)			Change in slope	P-value
	Ldlr$^{-/-}$	0.80	p= 0.0057		
	Ldlr$^{-/-}$ *Diaph1*$^{-/-}$	0.84	p= 0.0026	-7,418 μm^2 / %	0.15
B).	Atherosclerotic Lesion Area (H&E) vs. Macrophage Content (CD68)				
	Ldlr$^{-/-}$	0.93	p=0.00028		
	Ldlr$^{-/-}$ *Diaph1*$^{-/-}$	0.78	p= 0.0134	-5,953 μm^2 / %	0.19
C).	Macrophage Content (CD68) vs. Neutral Lipid Content (ORO)				
	Ldlr$^{-/-}$	0.93	p=0.0003		
	Ldlr$^{-/-}$ *Diaph1*$^{-/-}$	0.82	p= 0.0068	-0.16 %/%	0.54
D).	Atherosclerotic Lesion Area (H&E) vs. Plasma Cholesterol				
	Ldlr$^{-/-}$	0.83	p=0.0030		
	Ldlr$^{-/-}$*Diaph1*$^{-/-}$	0.86	p=0.0014	**-271 μm^2/ mg/dl**	**0.0082**
E).	Neutral Lipid Content (ORO) vs. Plasma Cholesterol				
	Ldlr$^{-/-}$	0.93	p<0.0001		
	Ldlr$^{-/-}$*Diaph1*$^{-/-}$	0.97	p<0.0001	-0.008 %/ mg/dL	0.017
F).	Macrophage Content (CD68) vs. Plasma Cholesterol				
	Ldlr$^{-/-}$	0.84	p= 0.0048		
	Ldlr$^{-/-}$*Diaph1*$^{-/-}$	0.92	p= 0.00038	-0.01 %/ mg/dL	0.053
G).	Atherosclerotic Lesion Area (H&E) vs. Plasma Triglyceride				
	Ldlr$^{-/-}$	0.53	p= 0.1117		
	Ldlr$^{-/-}$*Diaph1*$^{-/-}$	0.0183	p= 0.96	-80 μm^2/mg/dL	0.56
H).	Neutral Lipid Content (ORO) vs Plasma Triglyceride				
	Ldlr$^{-/-}$	0.51	p= 0.1326		
	Ldlr$^{-/-}$*Diaph1*$^{-/-}$	0.22	p= 05498	0.012 %/mg/dL	0.14
I).	Macrophage Content (CD68) vs Plasma Triglyceride				
	Ldlr$^{-/-}$	0.36	p= 0.3371		
	Ldlr$^{-/-}$*Diaph1*$^{-/-}$	0.10	p= 0.7924	0.0034 %/mg/dL	0.63
J).	Atherosclerotic Lesion Area (H&E) vs. Plasma Glucose				
	Ldlr$^{-/-}$	-0.09	p=0.80		
	Ldlr$^{-/-}$*Diaph1*$^{-/-}$	0.37	p= 0.29	969 μm^2 / mg/dl	0.58
K).	Atherosclerotic Lesion Area (H&E) vs. Serum Insulin				
	Ldlr$^{-/-}$	-0.43	p= 0.21		
	Ldlr$^{-/-}$*Diaph1*$^{-/-}$	-0.35	p=0.32	-15,869 μm^2/ μg/L	0.84

Supplementary Table 9. Correlation Analysis of the Atherosclerotic Lesion Area vs. lipid and glucose-related factors in Female *Ldlr*$^{-/-}$ vs. *Ldlr*$^{-/-}$*Diaph1*$^{-/-}$ Mice on WD for 16 Weeks

**Genotype/Variable**		**Correlation Coefficient/P-value**		***Ldlr*$^{-/-}$ *Diaph1*$^{-/-}$ vs. *Ldlr*$^{-/-}$**	
A).	Atherosclerotic Lesion Area (H&E) vs. Plasma Cholesterol			Change in slope	P-value
	Ldlr$^{-/-}$	-0.12	p=0.7755		
	Ldlr$^{-/-}$*Diaph1*$^{-/-}$	0.70	p=0.0364	**2,070 µm^2/ mg/dl**	**0.0224**
B).	Atherosclerotic Lesion Area (H&E) vs. Plasma Triglyceride				
	Ldlr$^{-/-}$	0.003	p= 0.9948		
	Ldlr$^{-/-}$*Diaph1*$^{-/-}$	0.20	p= 0.6105	1,150 µm^2/mg/dL	0.6316
C).	Atherosclerotic Lesion Area (H&E) vs. Plasma Glucose				
	Ldlr$^{-/-}$	-0.05	p=0.8955		
	Ldlr$^{-/-}$*Diaph1*$^{-/-}$	0.43	p=0.2522	4,457 µm^2 / mg/dl	0.2417
