# Supplementary material for: DIAPH1 mediates progression of atherosclerosis and regulates hepatic lipid metabolism in mice
Source: Commun Biol. 2023 Mar 17;6:280. doi: 10.1038/s42003-023-04643-2 (PMC10023694; doi:10.1038/s42003-023-04643-2)
Supplement: Supplementary file 2 — Description of Additional Supplementary Files [file 42003_2023_4643_MOESM2_ESM.pdf]

Description of Additional Supplementary Files

File name: Supplementary Data 1

Description: Source data behind graphs in main manuscript

File name: Supplementary Data 2

Description: Source data behind graphs in supplementary file

File name: Supplementary Data 3

Description: Uncropped blots for Figure 5A-C, Figure 6A-F, Figure 8A-B, Sup Figure 7A-C, Sup Figure 9A-E, Sup Figure 10A-C

File name: Supplementary Data 4

Description: Uncropped blots for Figure 5D.

File name: Supplementary Data 5

Description: Uncropped blots for Figure 8C

File name: Supplementary Data 6

Description: Differentially expressed genes in *Ldlr*$^{-/-}$*Diaph1*$^{-/-}$ *vs. Ldlr*$^{-/-}$ with FDR < 0.05
