# Supplementary material for: DIAPH1 mediates progression of atherosclerosis and regulates hepatic lipid metabolism in mice
Source: Commun Biol. 2023 Mar 17;6:280. doi: 10.1038/s42003-023-04643-2 (PMC10023694; doi:10.1038/s42003-023-04643-2)
Supplement: Supplementary file 5 — Supplementary Data 3 [file 42003_2023_4643_MOESM5_ESM.pdf]

Figure 5A-B-C

Exp. Code: 062-4-FRACTIONATION in livers

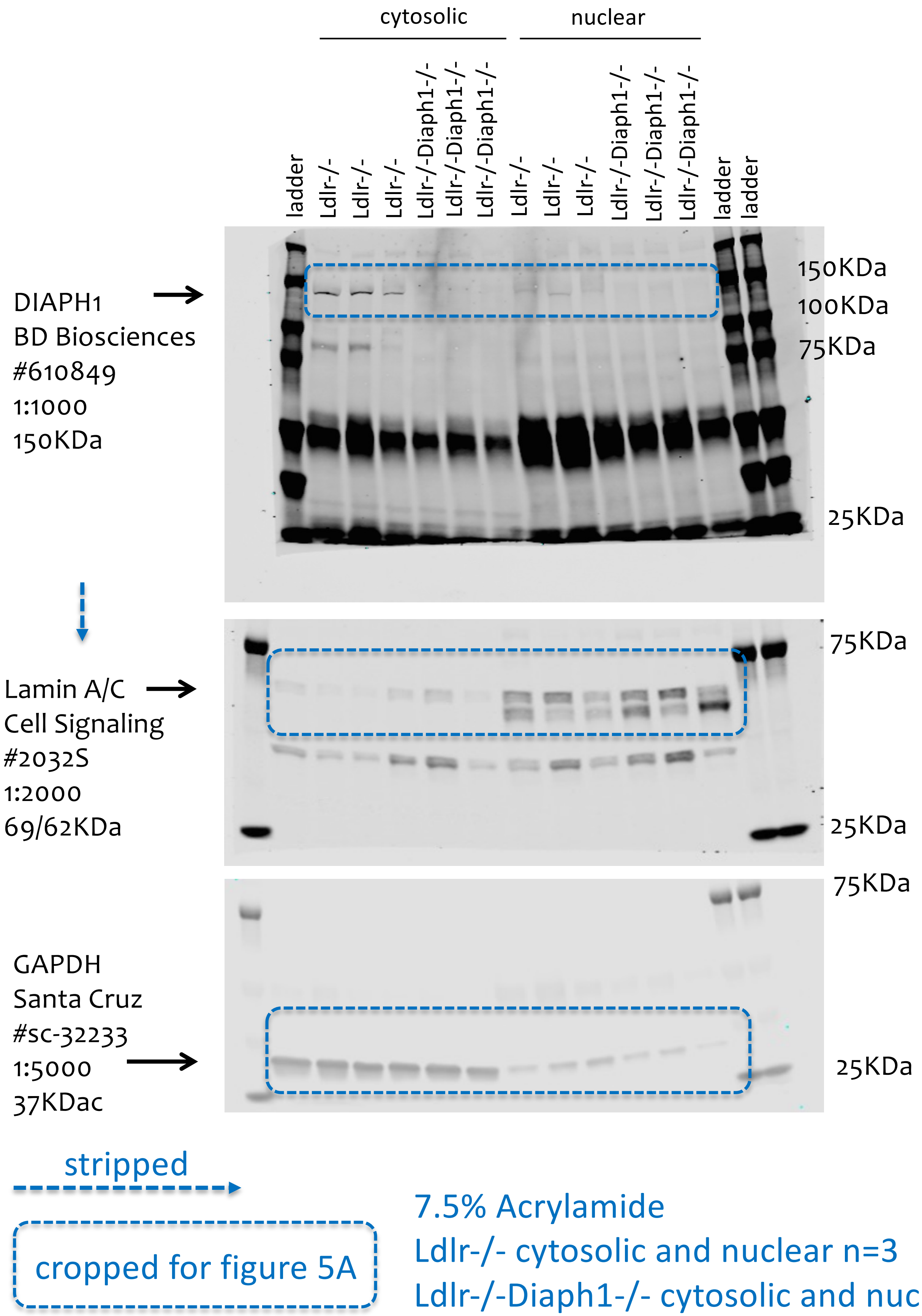

7.5% Acrylamide

Ldlr-/- cytosolic and nuclear n=3

Ldlr-/-Diaph1-/- cytosolic and nuclear n=3

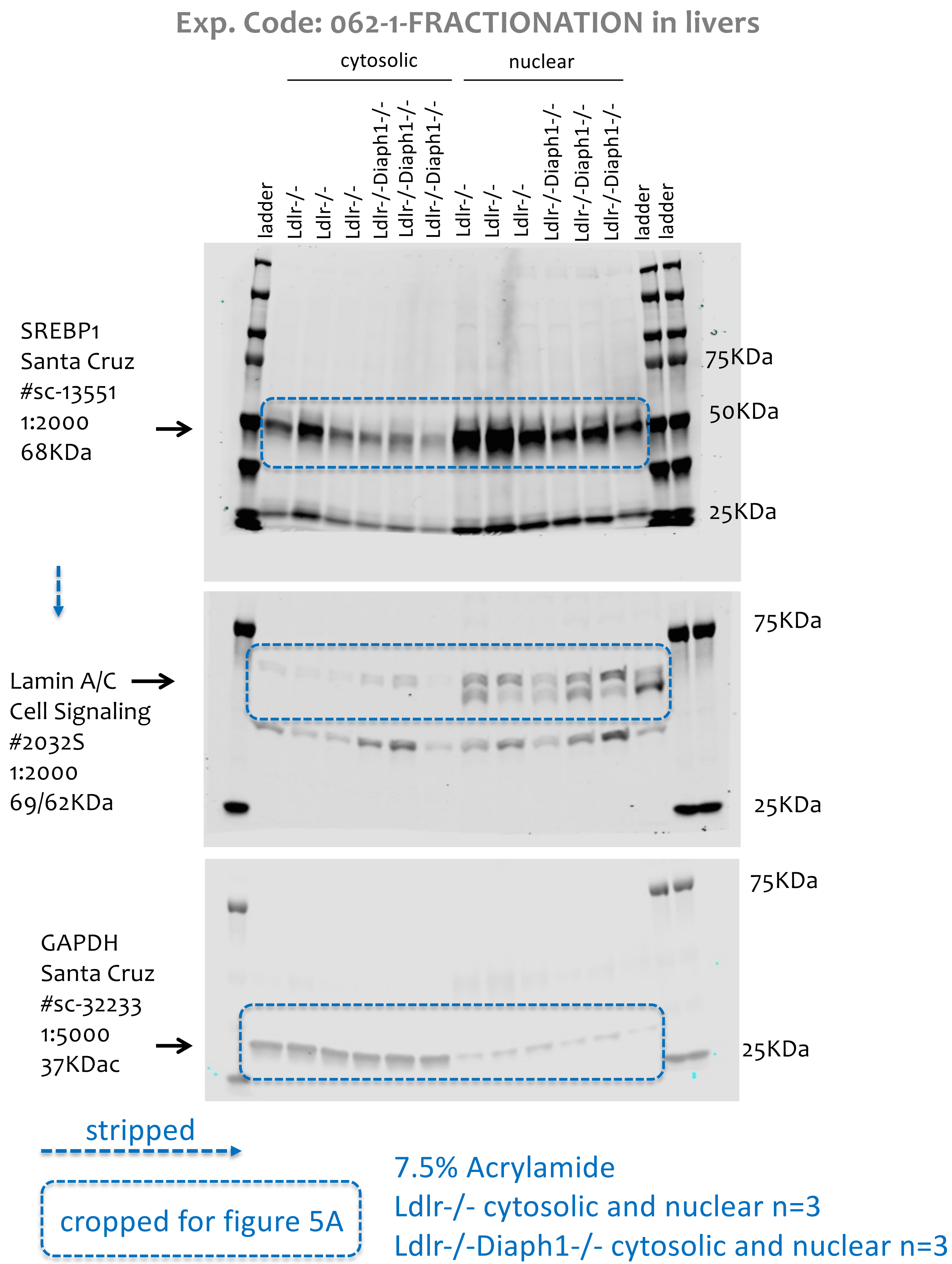

Exp. Code: 062-1-FRACTIONATION in livers
cytosolic
nuclear
ladder
Ldlr-/-
Ldlr-/-
Ldlr-/-
Ldlr-/-Diaph1-/-
Ldlr-/-Diaph1-/-
Ldlr-/-Diaph1-/-
Ldlr-/-
Ldlr-/-
Ldlr-/-
Ldlr-/-Diaph1-/-
Ldlr-/-Diaph1-/-
Ldlr-/-Diaph1-/-
ladder
ladder
SREBP1
Santa Cruz
#sc-13551
1:2000
68KDa
75KDa
50KDa
25KDa
Lamin A/C
Cell Signaling
#2032S
1:2000
69/62KDa
75KDa
25KDa
GAPDH
Santa Cruz
#sc-32233
1:5000
37KDac
75KDa
25KDa
stripped
cropped for figure 5A
7.5% Acrylamide
Ldlr-/- cytosolic and nuclear n=3
Ldlr-/-Diaph1-/- cytosolic and nuclear n=3

Exp. Code: 062-2-FRACTIONATION in livers

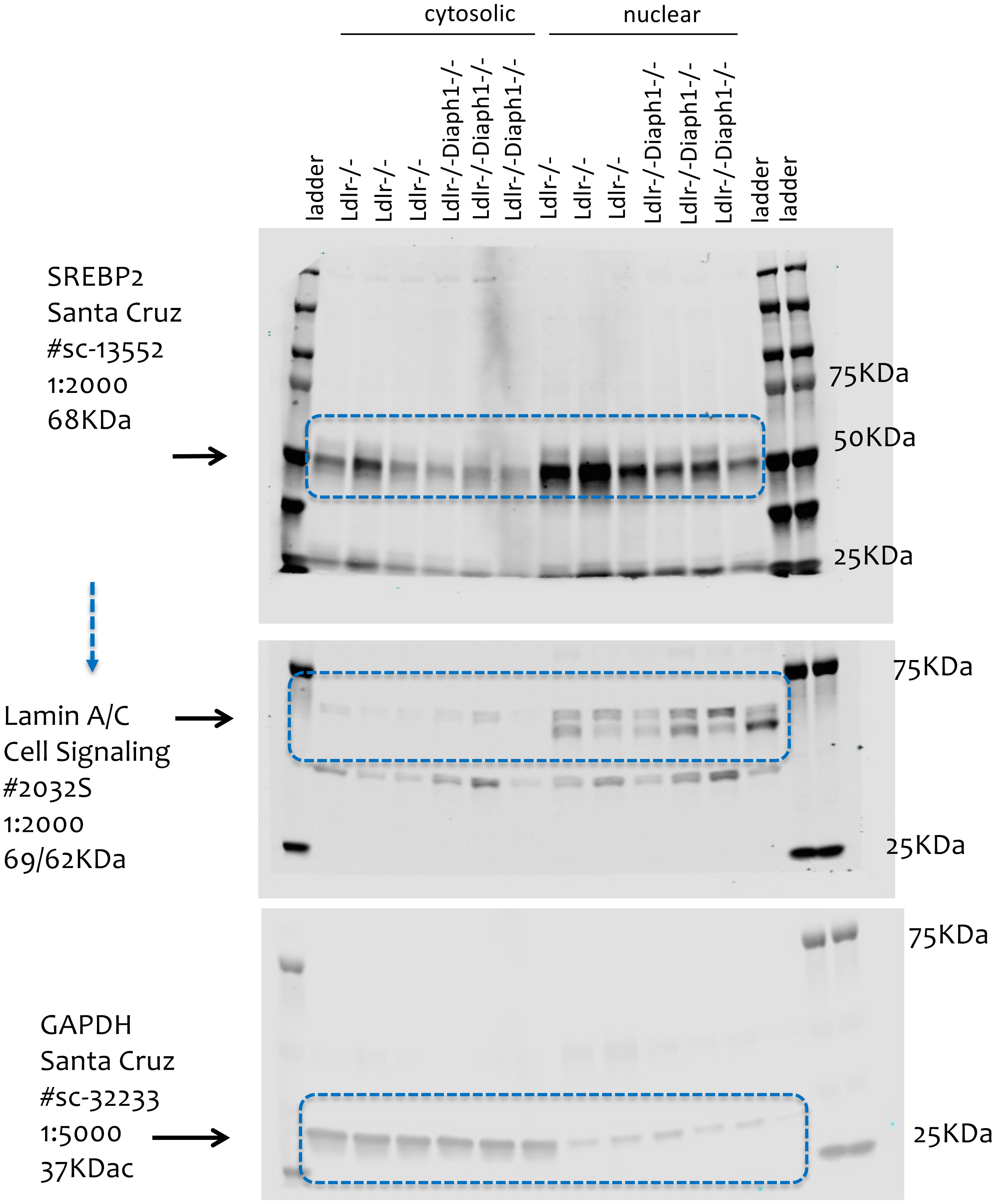

stripped

cropped for figure 5A

7.5% Acrylamide
Ldlr-/- cytosolic and nuclear n=3
Ldlr-/-Diaph1-/- cytosolic and nuclear n=3

Exp. Code: 062-3-FRACTIONATION in livers

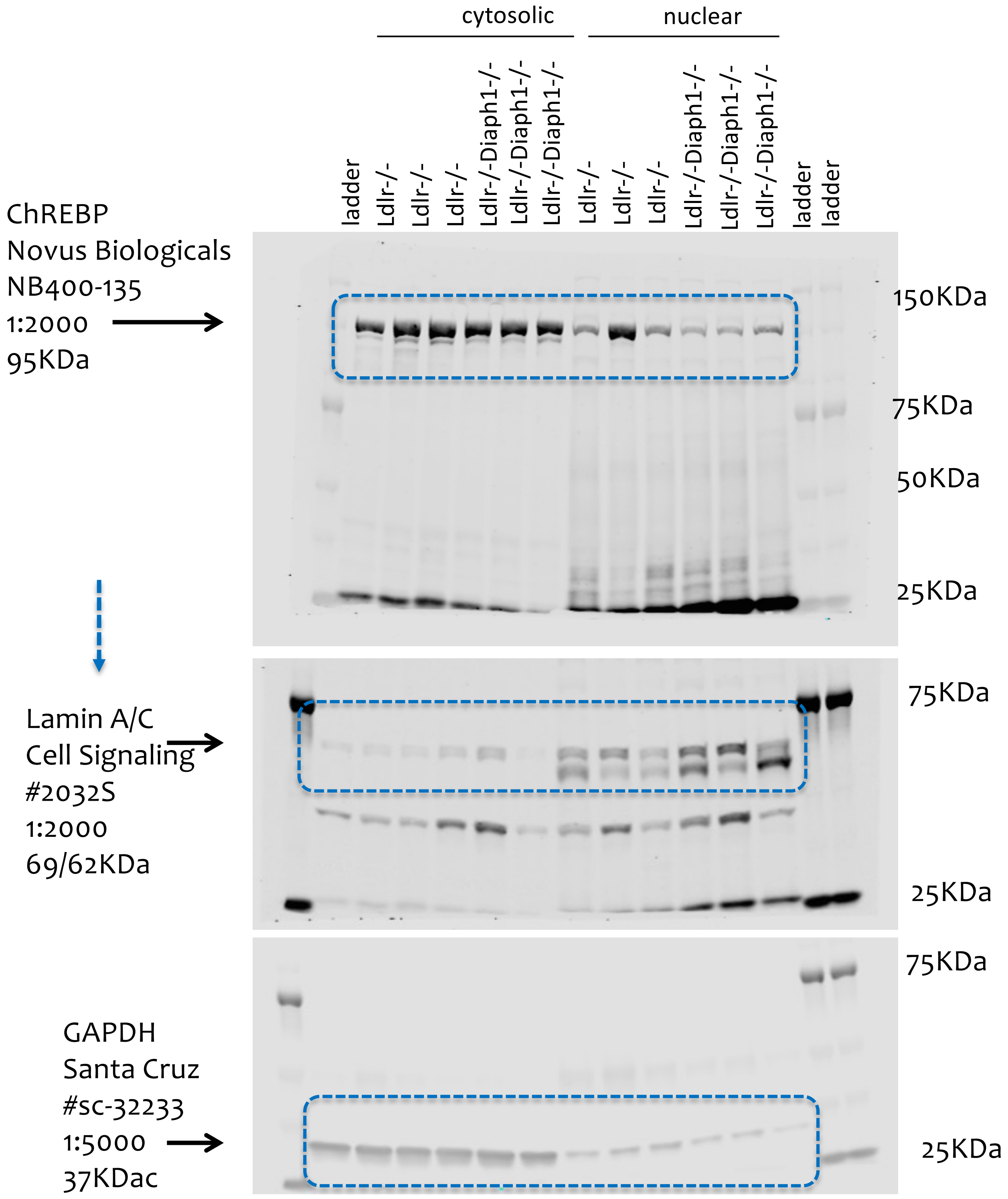

stripped

cropped for figure **5A**

7.5% Acrylamide

Ldlr-/- cytosolic and nuclear n=3

Ldlr-/-Diaph1-/- cytosolic and nuclear n=3

Exp. Code: 061-8-FRACTIONATION in livers

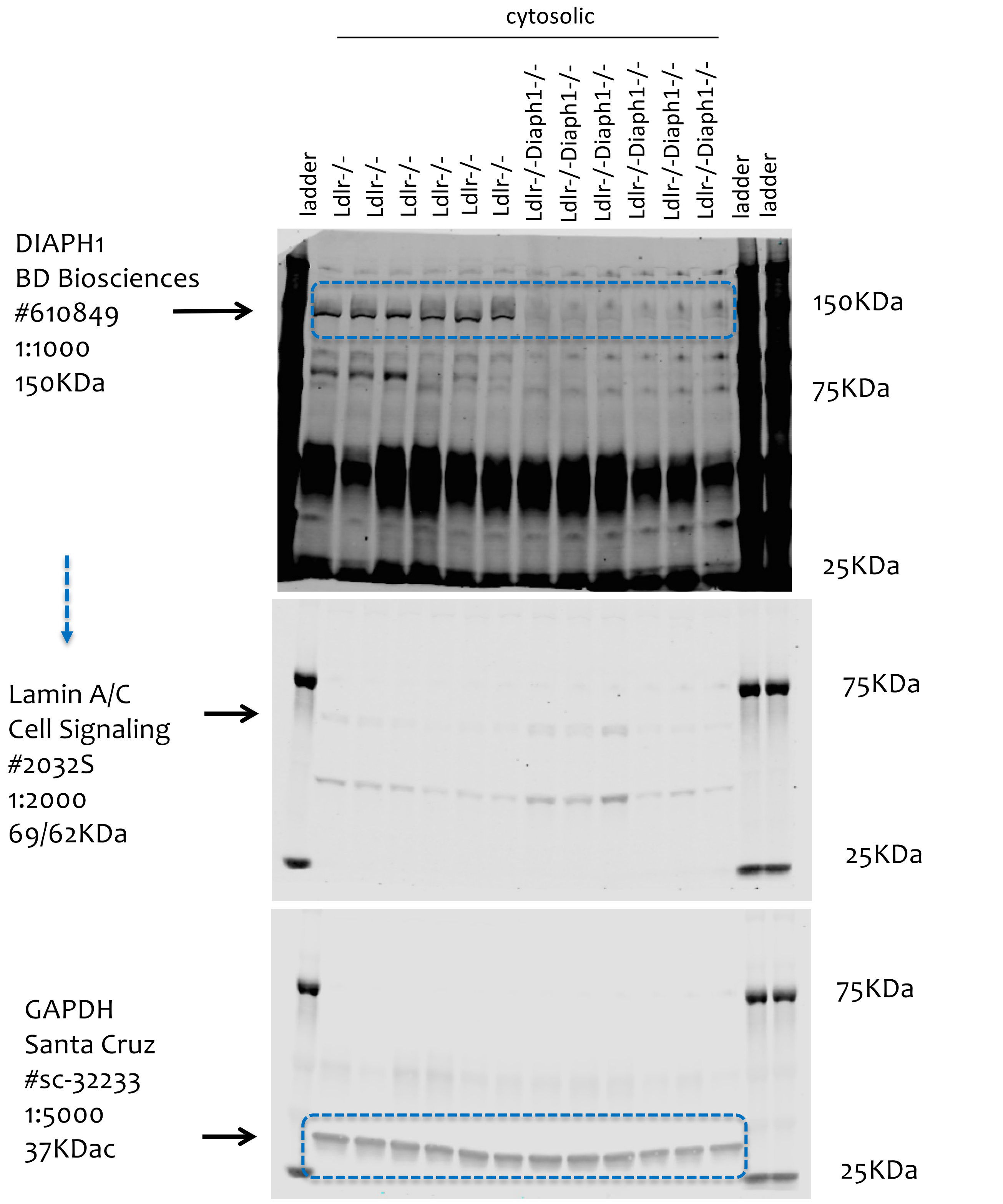

stripped

quantified in figure 5B

7.5% Acrylamide
Ldlr-/- cytosolic n=6
Ldlr-/-Diaph1-/- cytosolic n=6

Exp. Code: 061-5-FRACTIONATION in livers

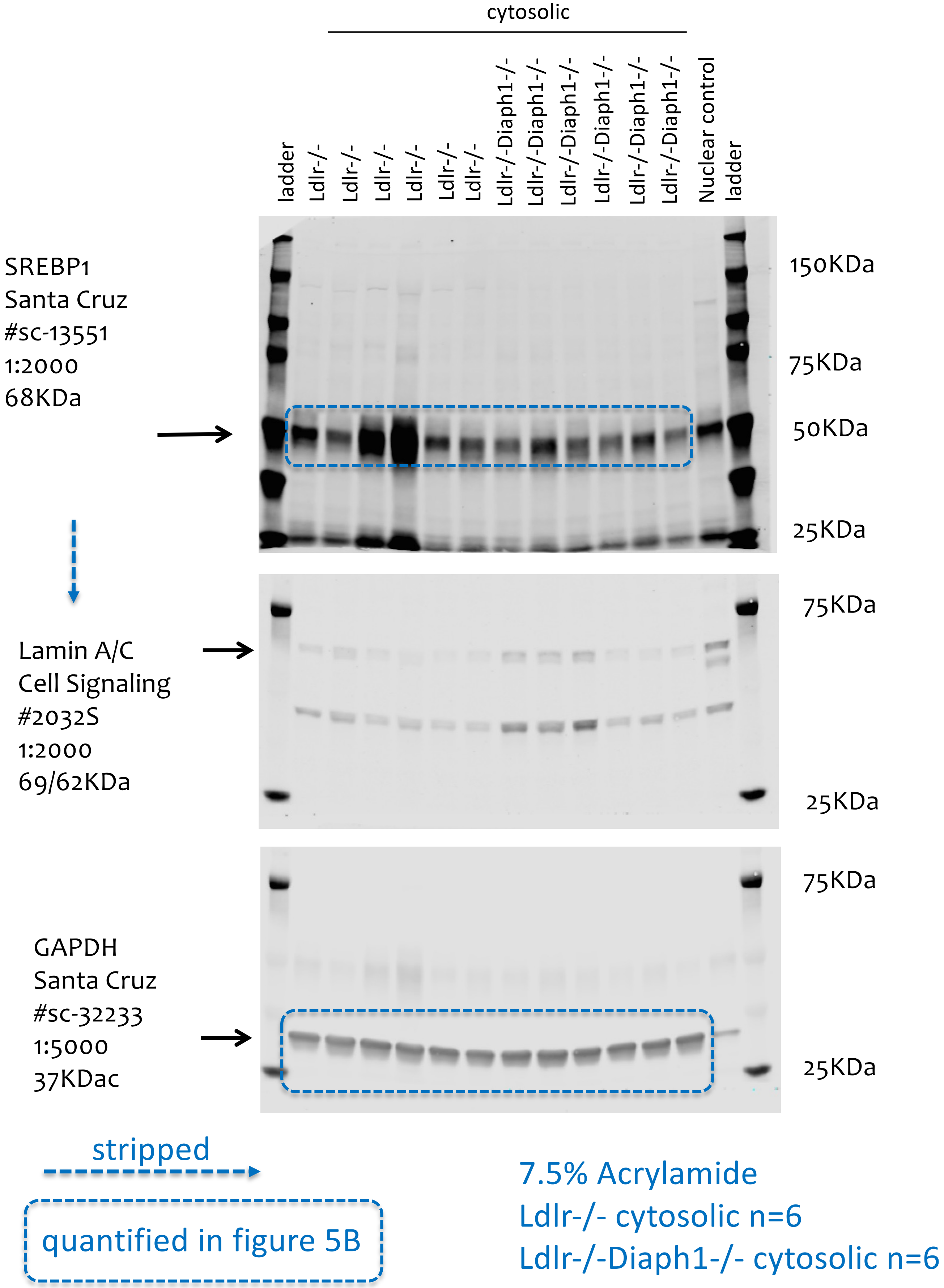

Exp. Code: 061-6-FRACTIONATION in livers

cytosolic

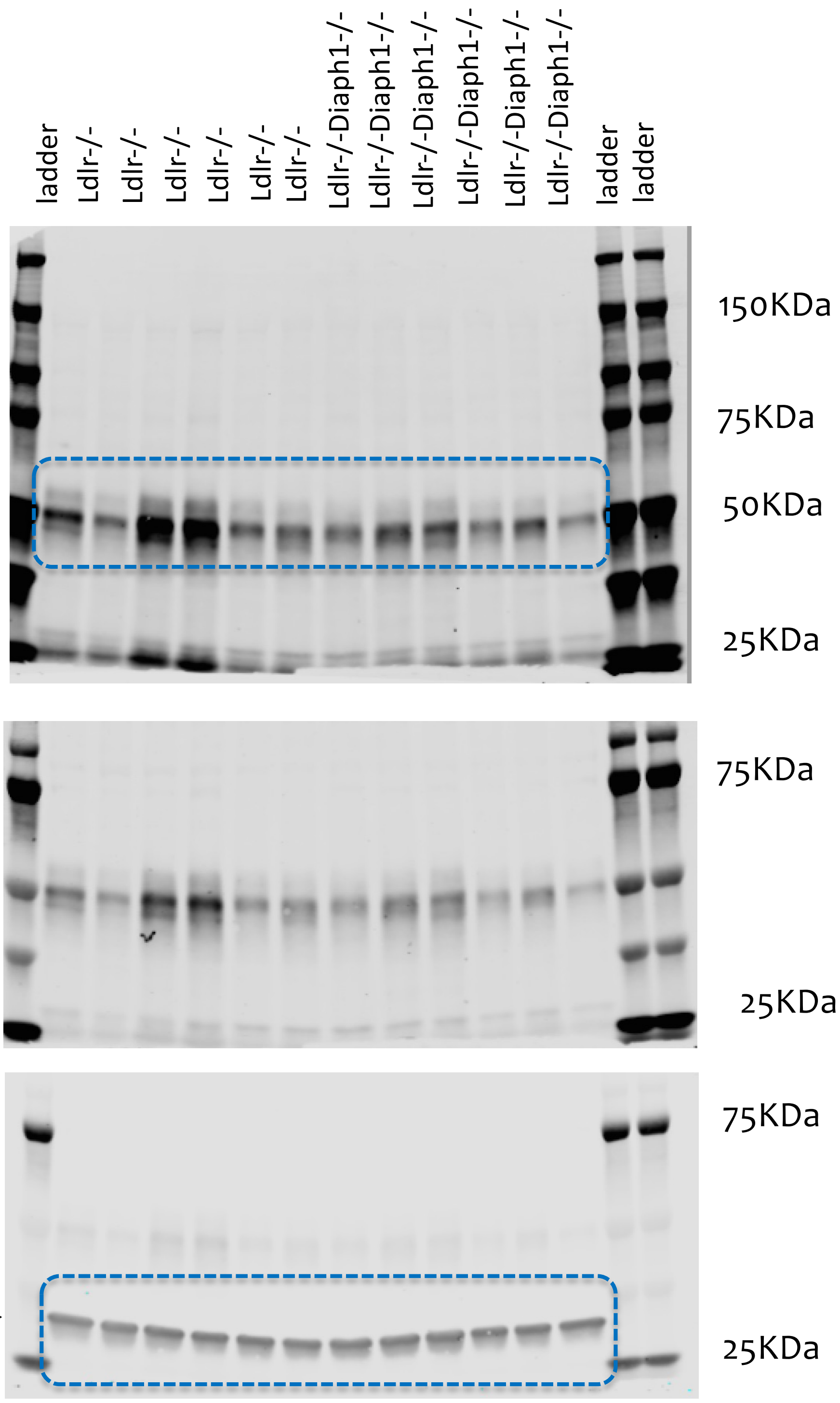

SREBP2
Santa Cruz
#sc-13552
1:2000
68KDa

Lamin A/C
Cell Signaling
#2032S
1:2000
69/62KDa

GAPDH
Santa Cruz
#sc-32233
1:5000
37KDac

stripped

quantified in figure 5B

7.5% Acrylamide
Ldlr-/- cytosolic n=6
Ldlr-/-Diaph1-/- cytosolic n=6

Exp. Code: 061-7-FRACTIONATION in livers

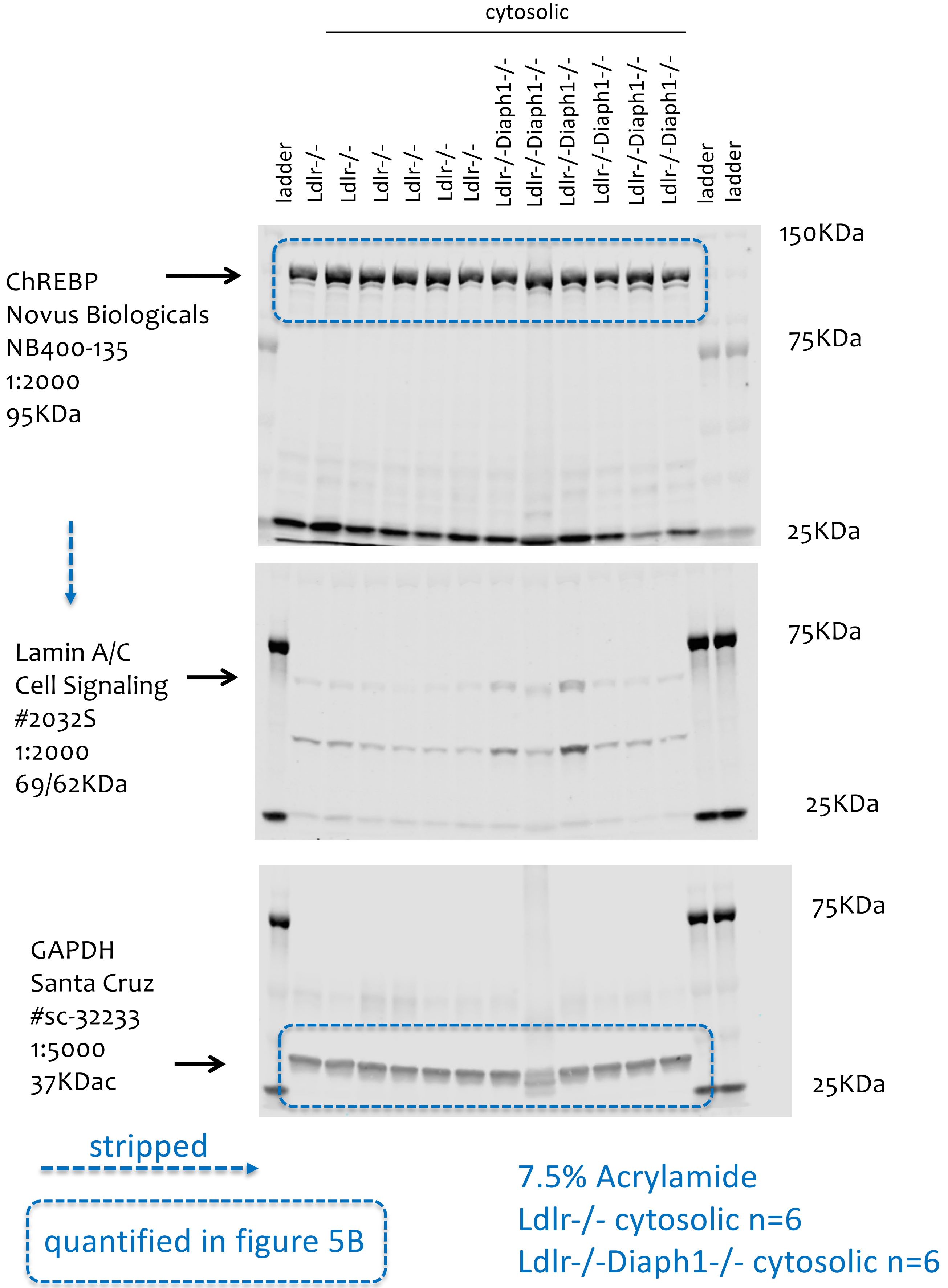

Exp. Code: 061-4-FRACTIONATION in livers

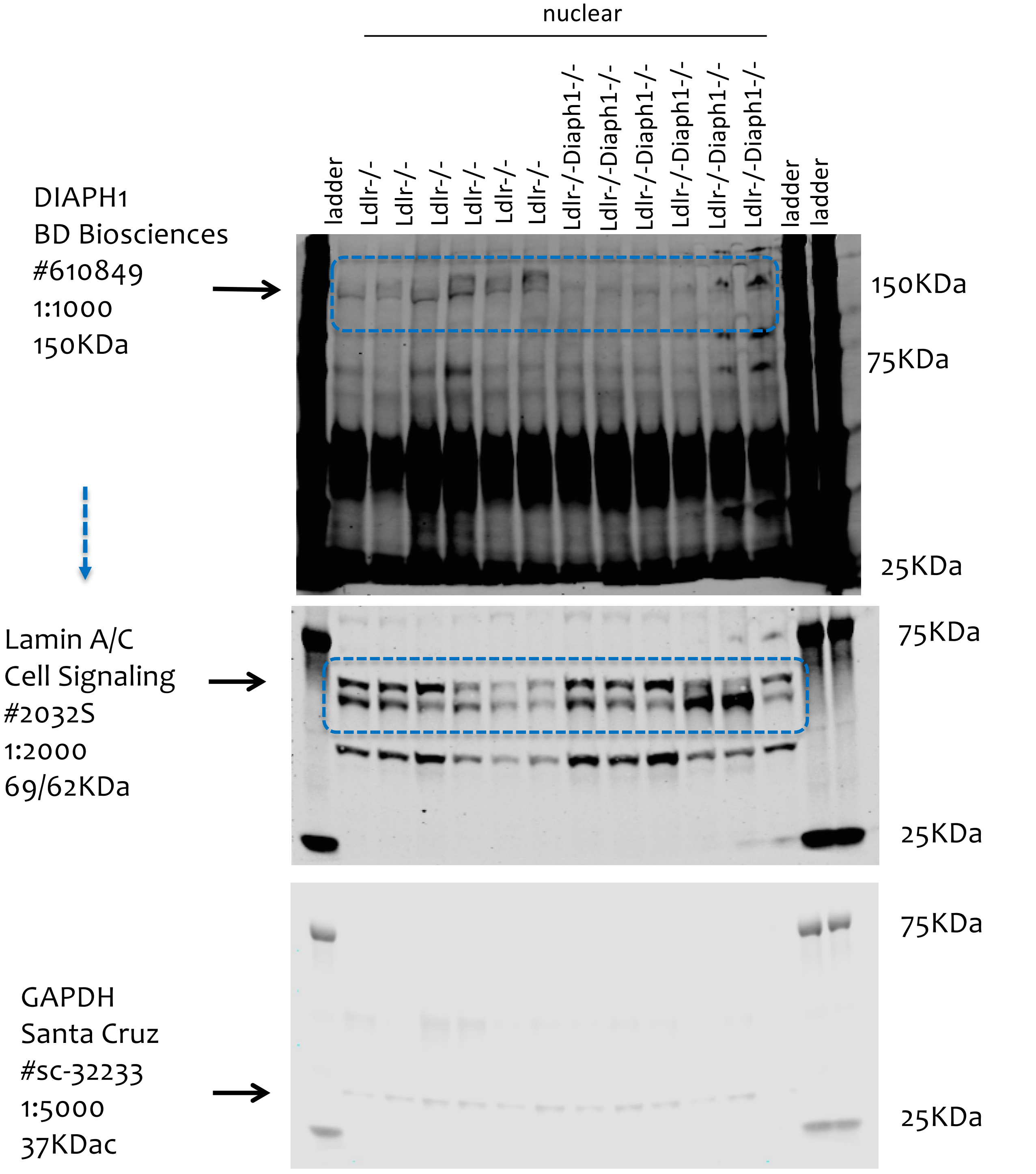

stripped

quantified in figure 5C

7.5% Acrylamide
Ldlr-/- nuclear n=6
Ldlr-/-Diaph1-/- nuclear n=6

Exp. Code: 061-1-FRACTIONATION in livers

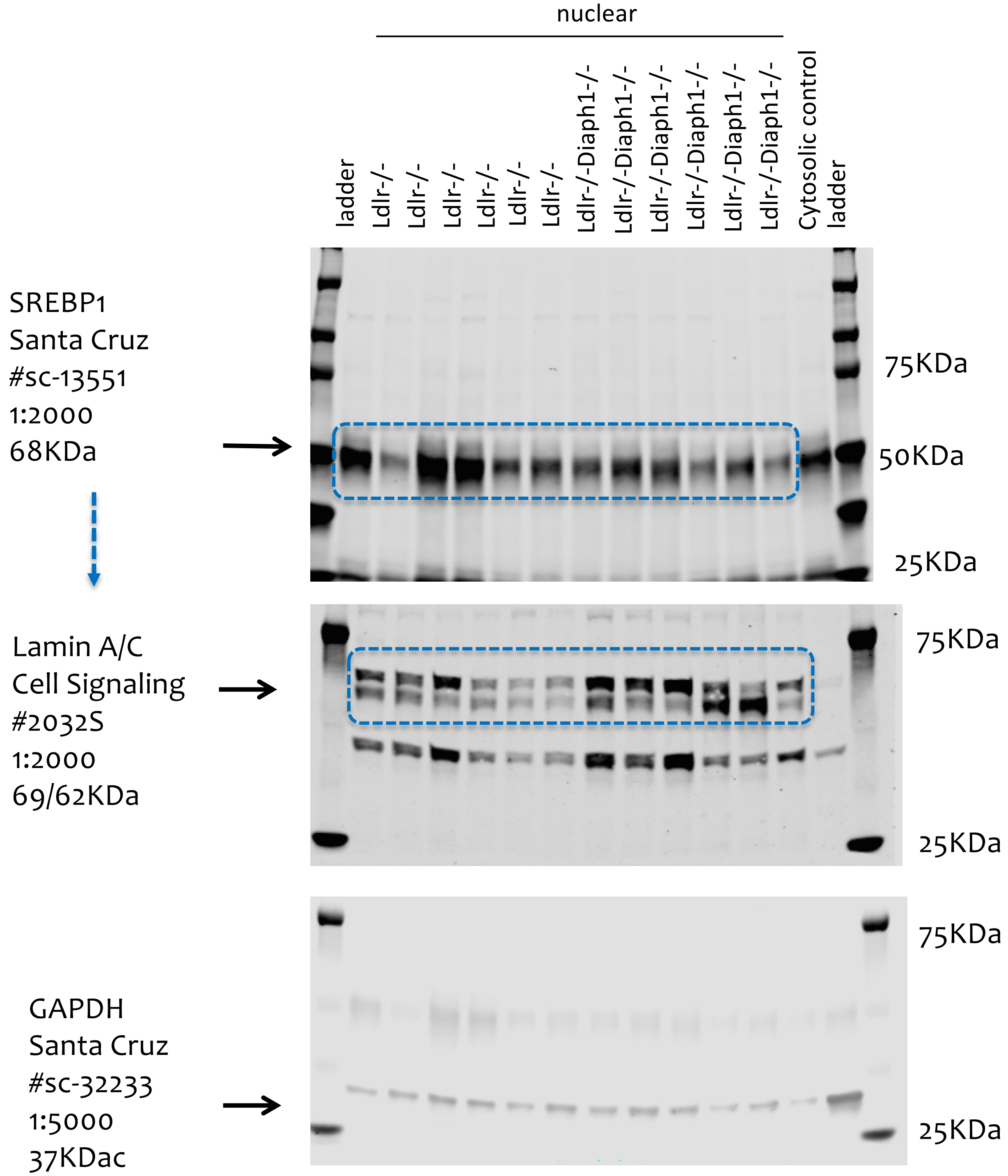

stripped

quantified in figure 5C

7.5% Acrylamide
Ldlr-/- nuclear n=6
Ldlr-/-Diaph1-/- nuclear n=6

Exp. Code: 061-2-FRACTIONATION in livers

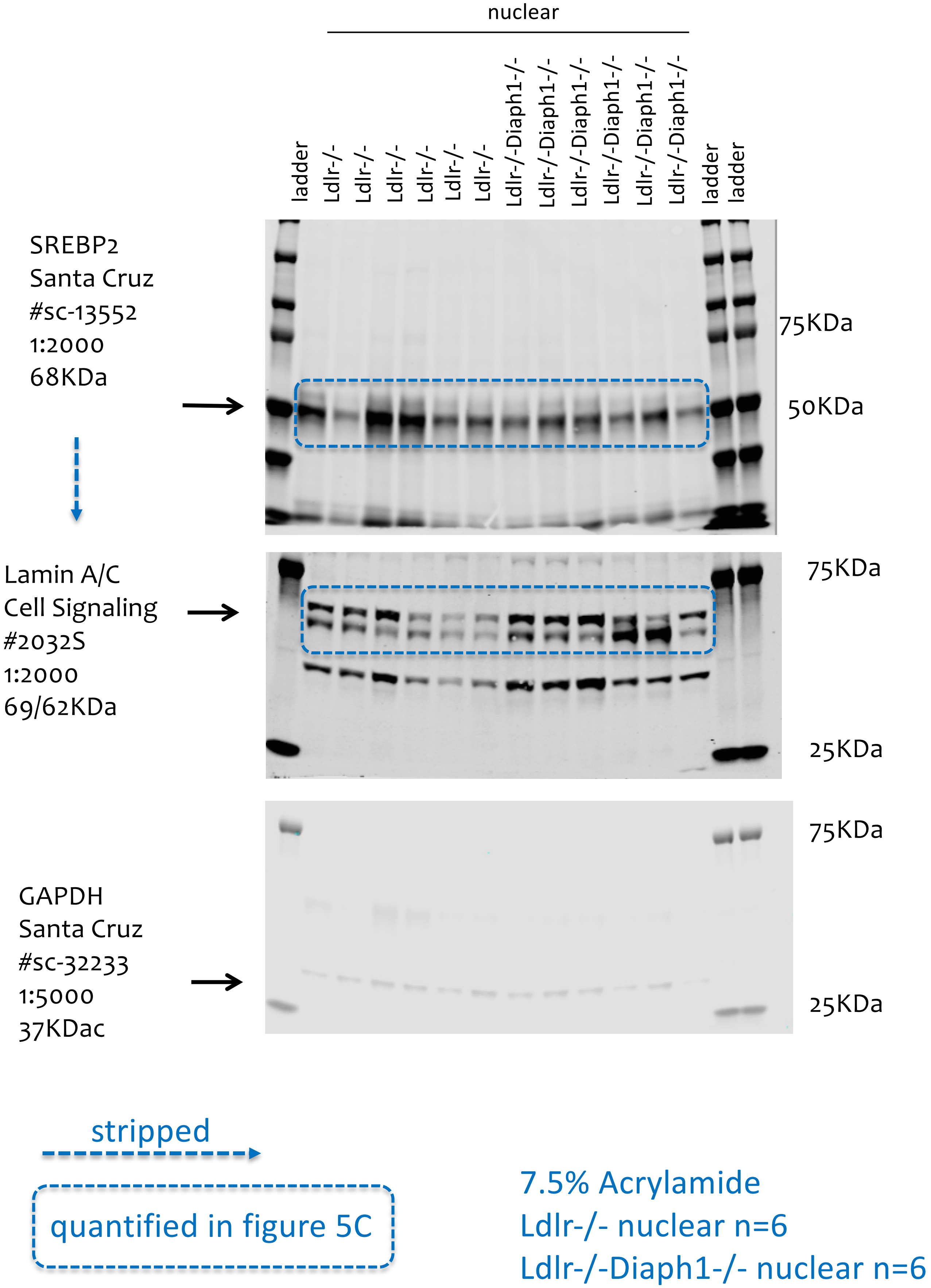

Exp. Code: 061-3-FRACTIONATION in livers

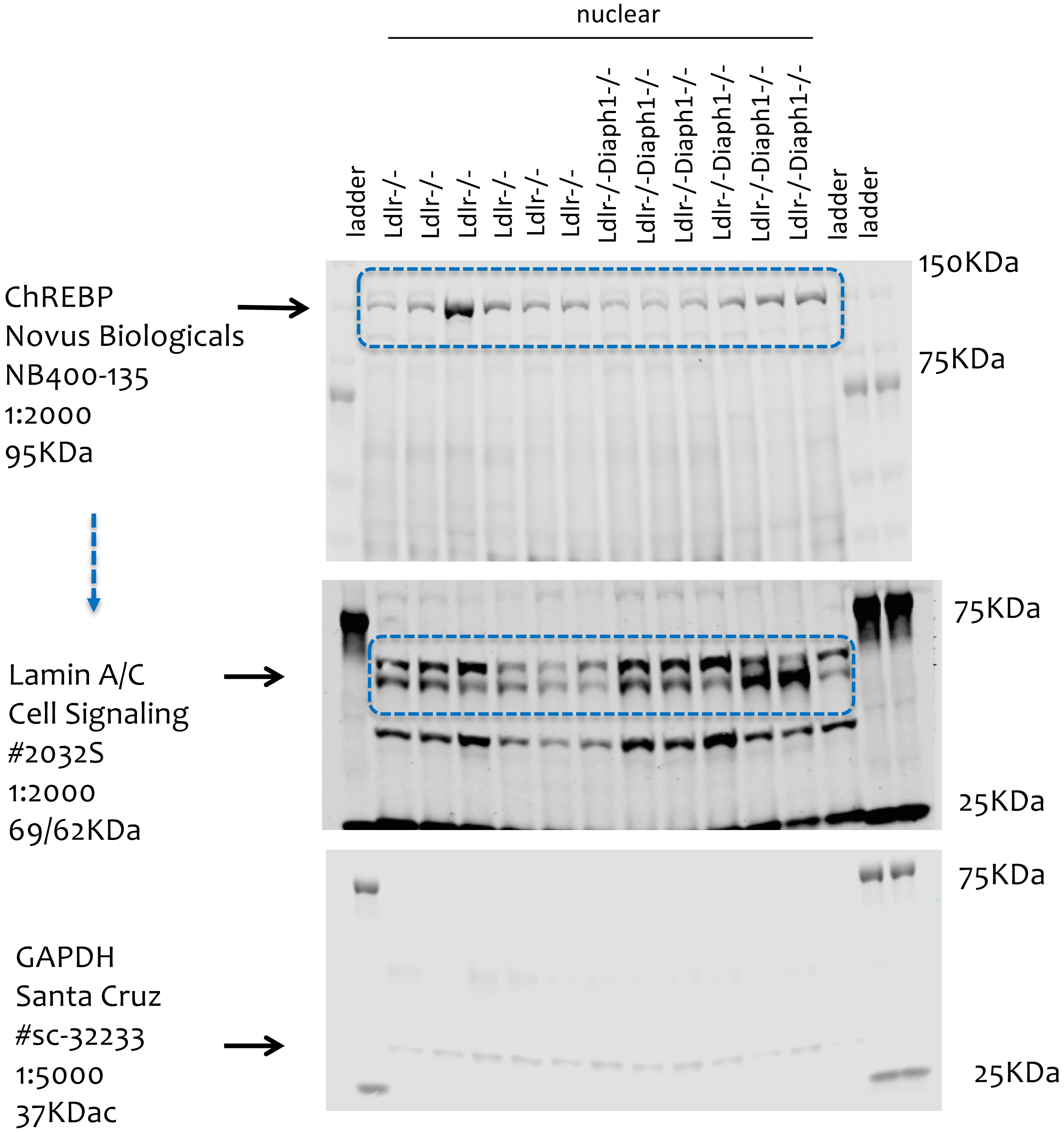

stripped

quantified in figure 5C

7.5% Acrylamide
Ldlr-/- nuclear n=6
Ldlr-/-Diaph1-/- nuclear n=6

Figure 6A-B-C-D-E-F

Exp. Code: 065-1-TOTAL LYSATE in livers

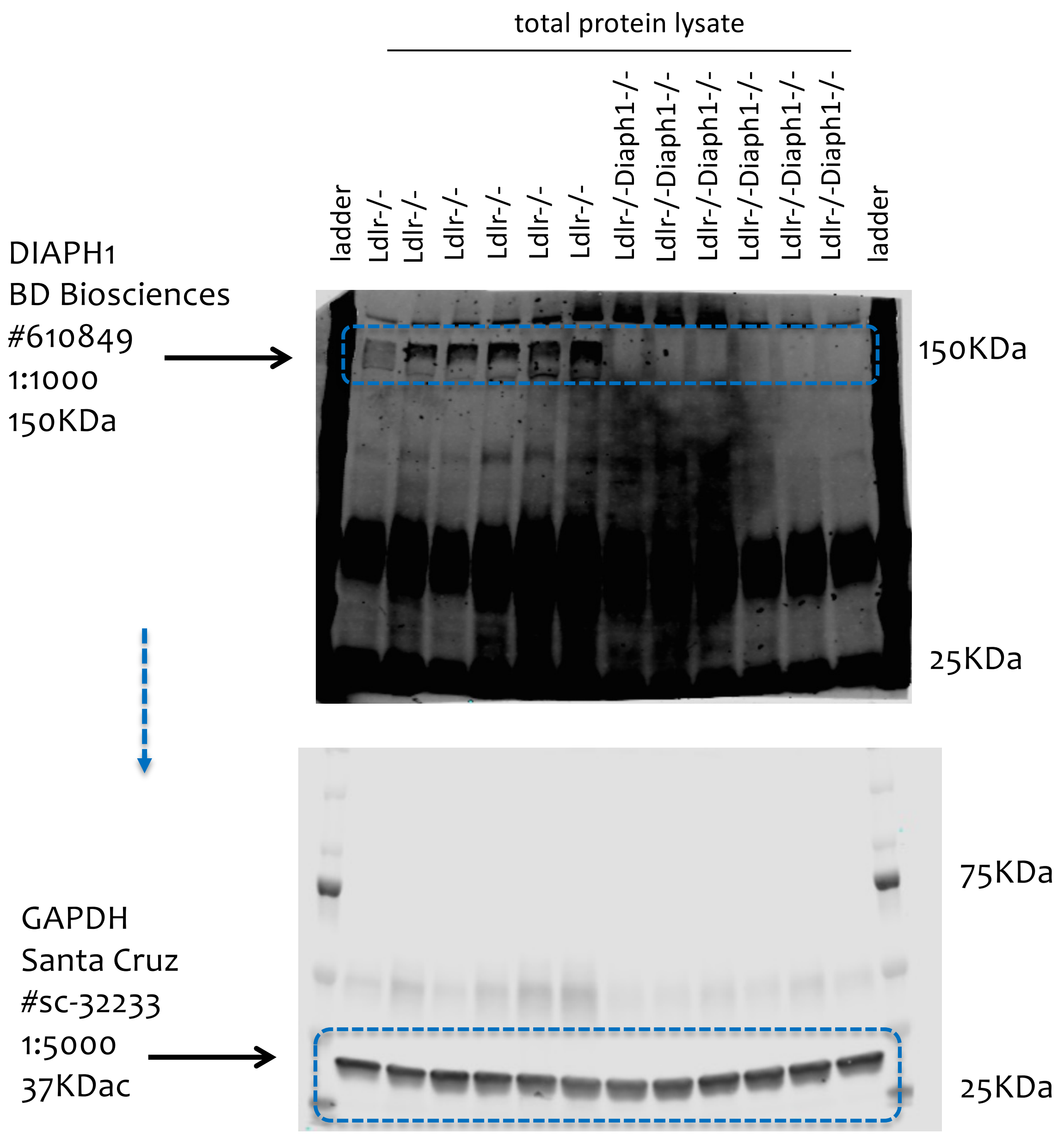

stripped

cropped for figure 6A
quantified in figure 6B

7.5% Acrylamide
Ldlr-/- n=6
Ldlr-/-Diaph1-/- n=6

Exp. Code: 065-14-TOTAL LYSATE in livers

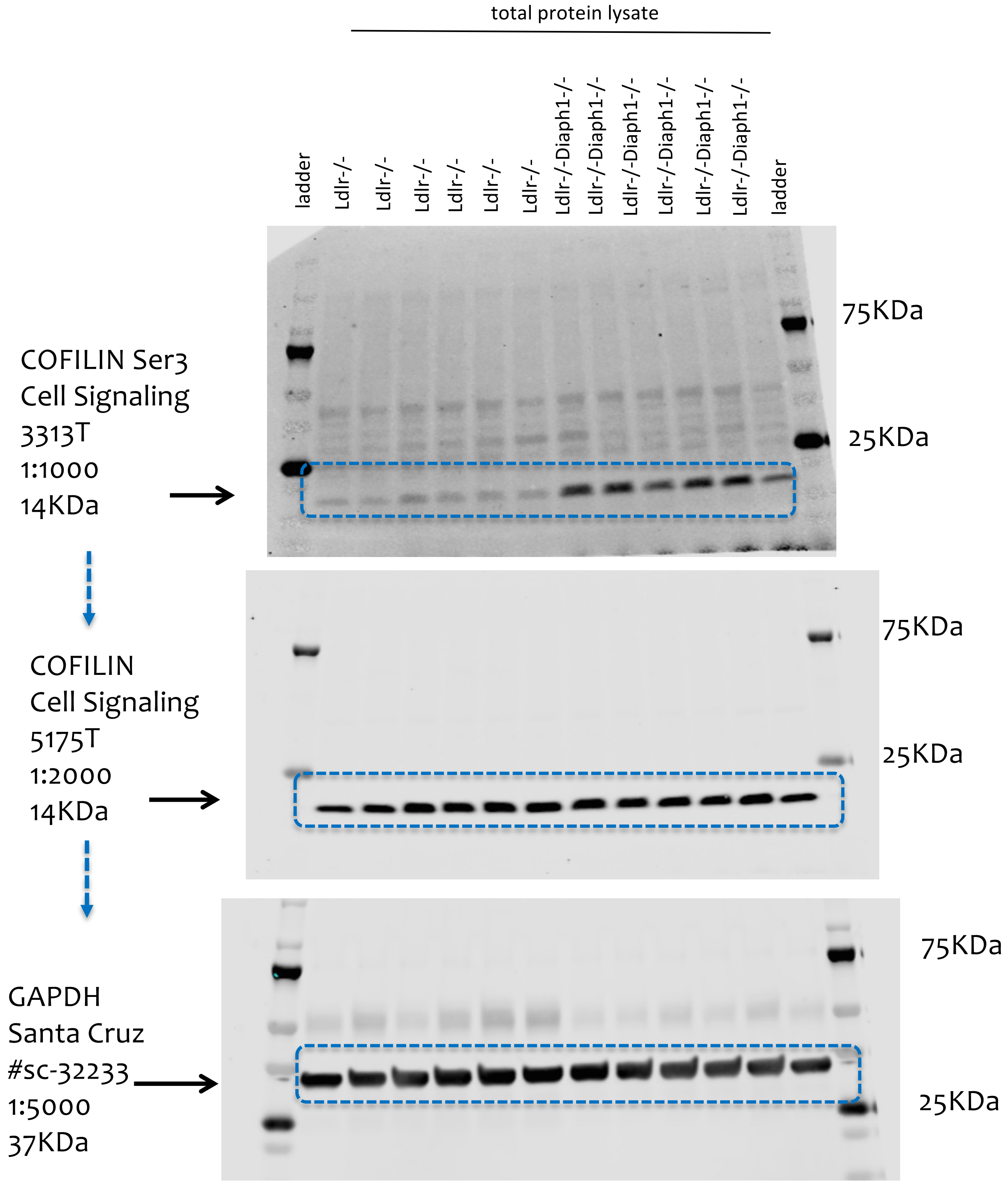

stripped

cropped for figure 6A
quantified in figure 6C

4-20% Acrylamide
Ldlr-/- n=6
Ldlr-/-Diaph1-/- n=6

Exp. Code: 065-10-TOTAL LYSATE in livers

total protein lysate

ladder
Ldlr-/-
Ldlr-/-
Ldlr-/-
Ldlr-/-
Ldlr-/-
Ldlr-/-
Ldlr-/-Diaph1-/-
Ldlr-/-Diaph1-/-
Ldlr-/-Diaph1-/-
Ldlr-/-Diaph1-/-
Ldlr-/-Diaph1-/-
Ldlr-/-Diaph1-/-
ladder
ladder

ROCK1
Cell Signaling
4035T
1:2000
160KDa

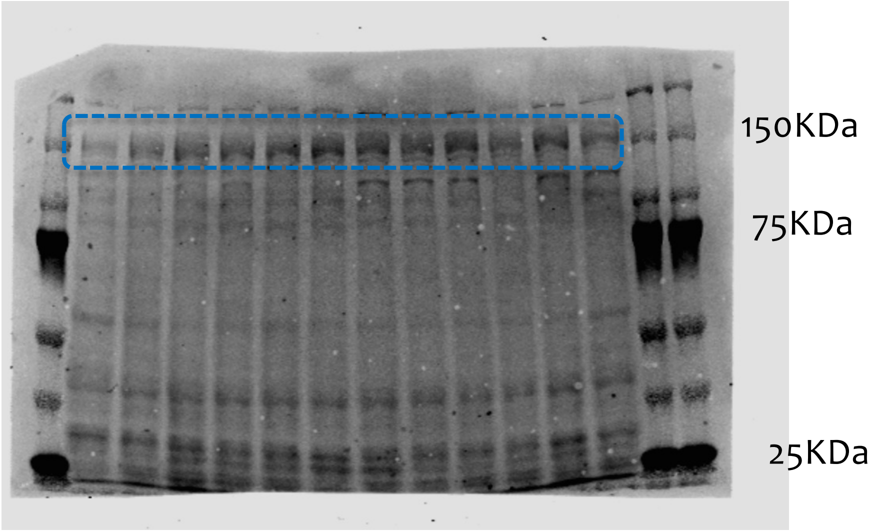

150KDa
75KDa
25KDa

GAPDH
Santa Cruz
#sc-32233
1:5000
37KDa

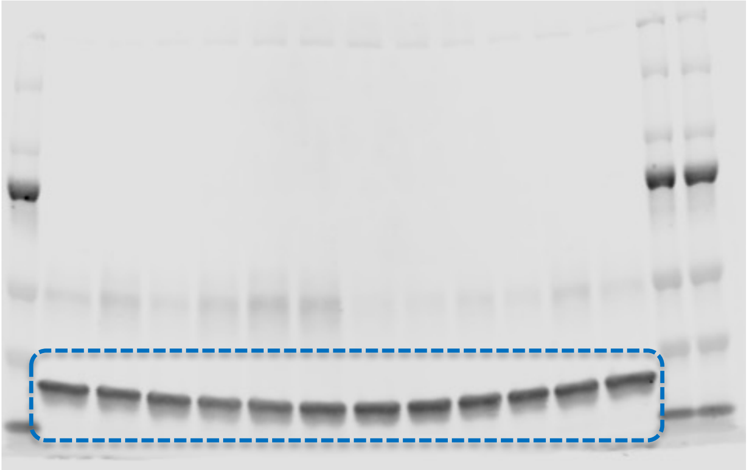

75KDa
25KDa

stripped

cropped for figure 6A
quantified in figure 6D

7.5% Acrylamide
Ldlr-/- n=6
Ldlr-/-Diaph1-/- n=6

Exp. Code: 065-18-TOTAL LYSATE in livers

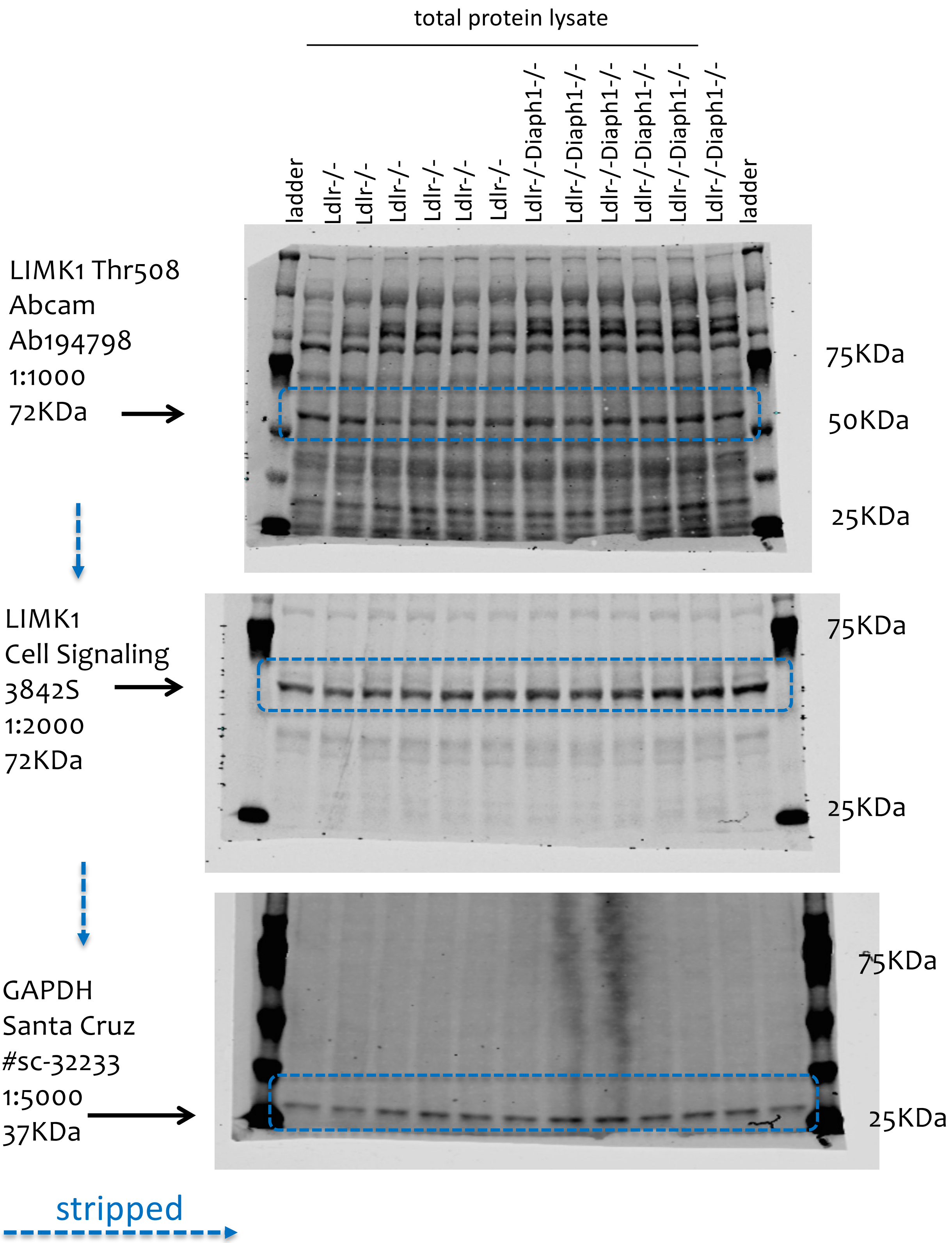

cropped for figure 6A
quantified in figure 6E

7.5% Acrylamide
Ldlr-/- n=6
Ldlr-/-Diaph1-/- n=6

Exp. Code: 065-6 (079)-TOTAL LYSATE in livers

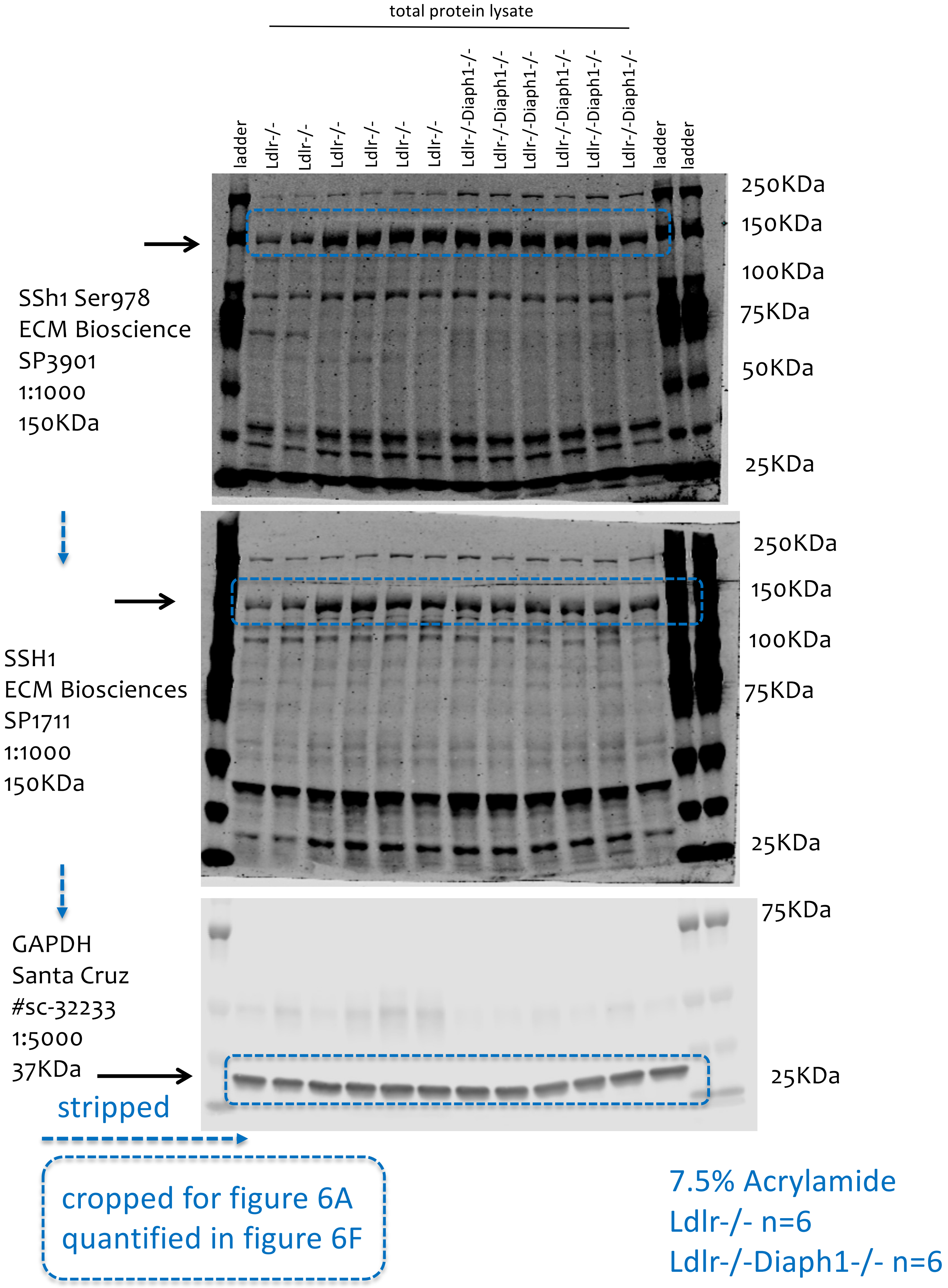

Figure 7A-B-C-D-E-F

Exp. Code: 068-1 *Diaph1* KD in Hepa 1-6 cells

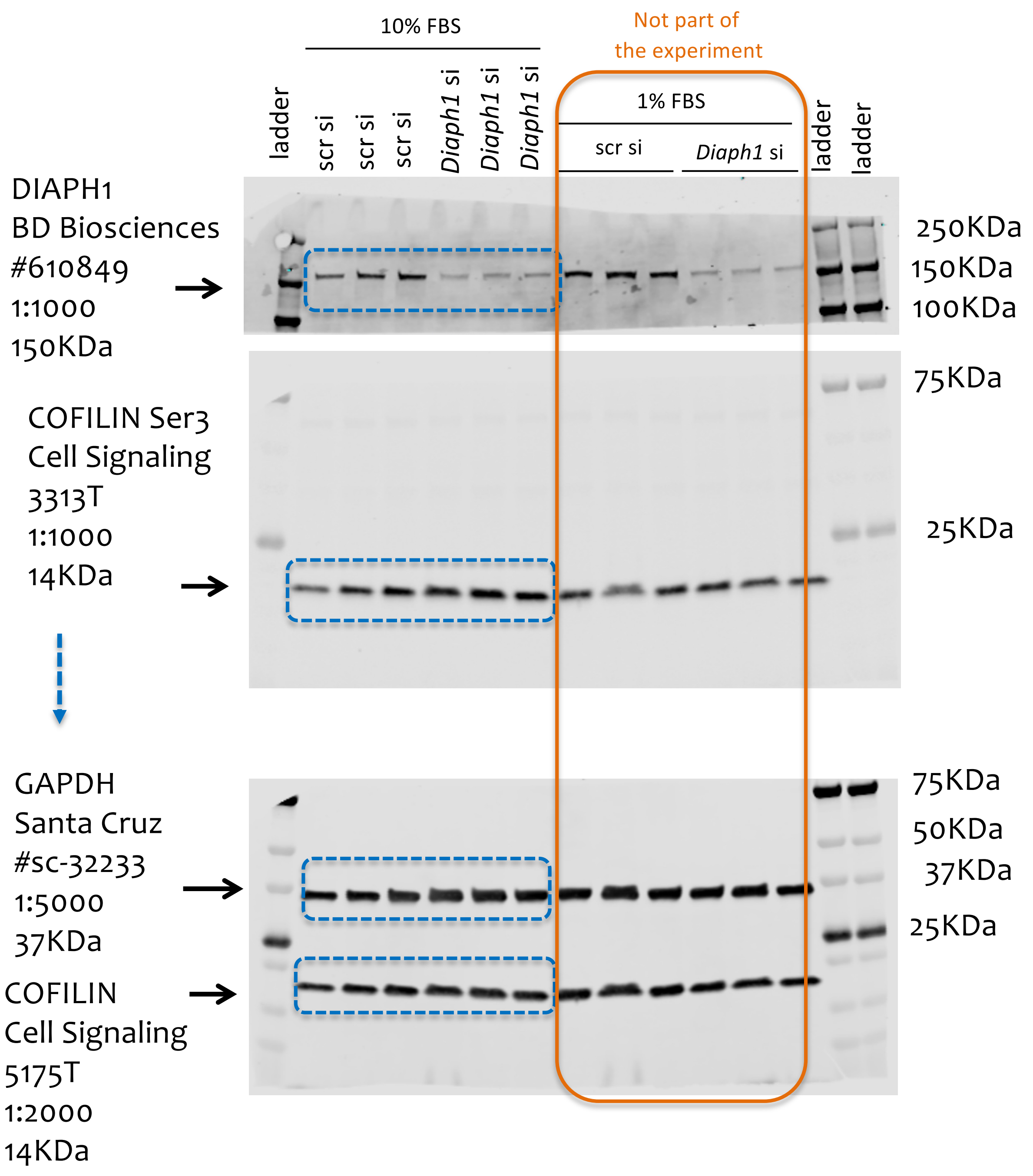

stripped

cropped for figure 7A
quantified in figure 7B, 7C

4-20% Acrylamide
Scr si n=3
Diaph1 si n=3

Exp. Code: 071-1 *Diaph1* KD in Hepa 1-6 cells

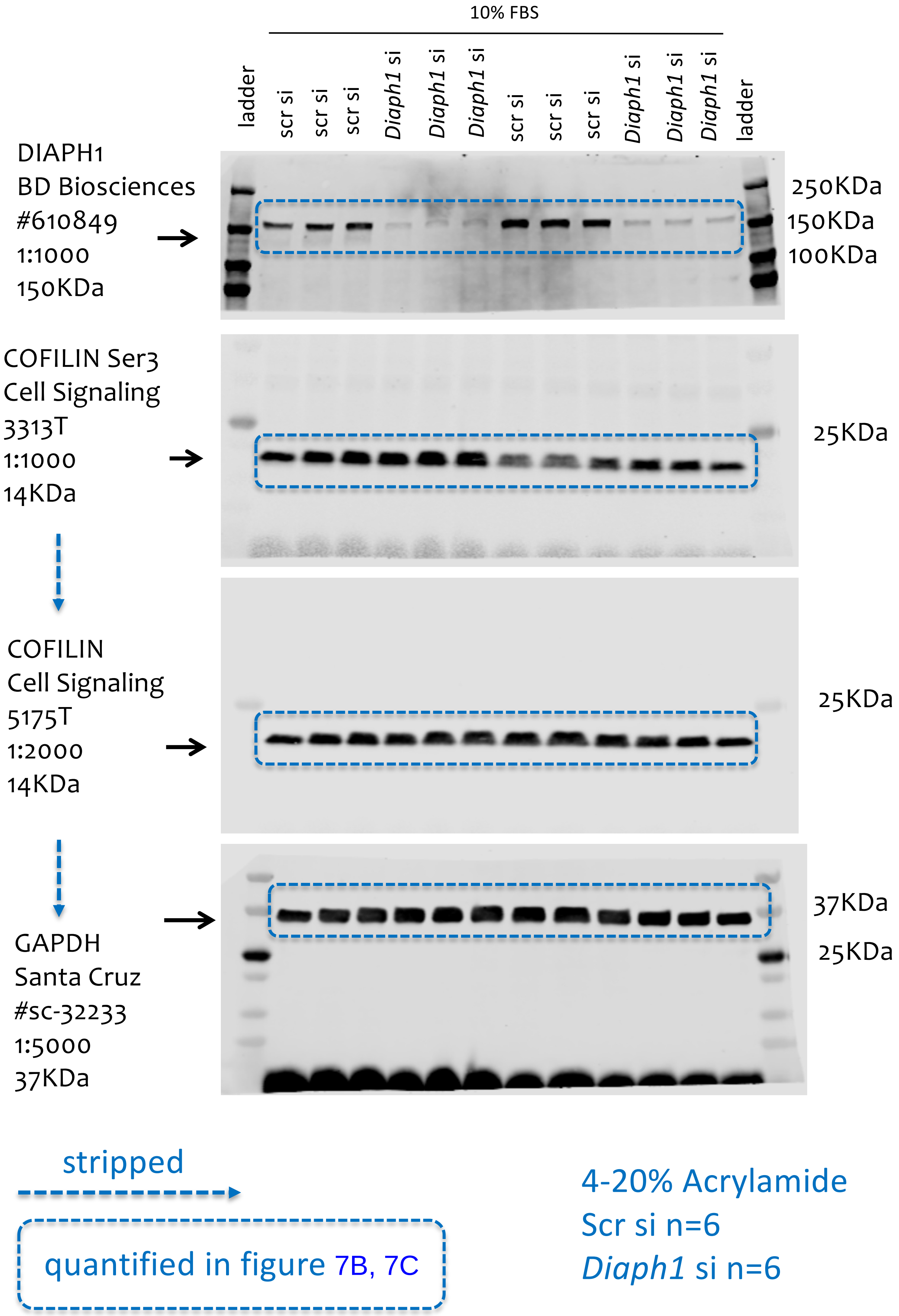

4-20% Acrylamide
Scr si n=6
Diaph1 si n=6

Exp. Code: 079 (071-1) Diaph1 KD in Hepa 1-6 cells

10% FBS

ladder | scr si | scr si | scr si | *Diaph1* si | *Diaph1* si | *Diaph1* si | scr si | scr si | scr si | *Diaph1* si | *Diaph1* si | *Diaph1* si | ladder

SSh1 Ser978
ECM Bioscience
SP3901
1:1000
150KDa

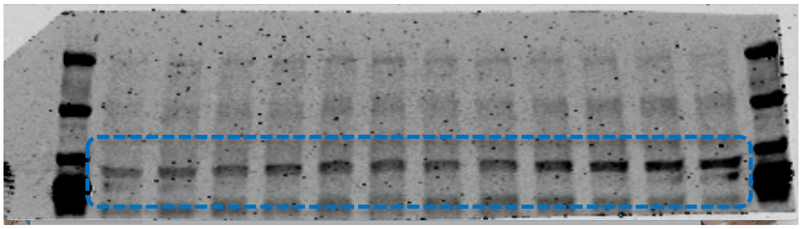

250KDa
150KDa
100KDa

SSH1
ECM Biosciences
SP1711
1:1000
150KDa

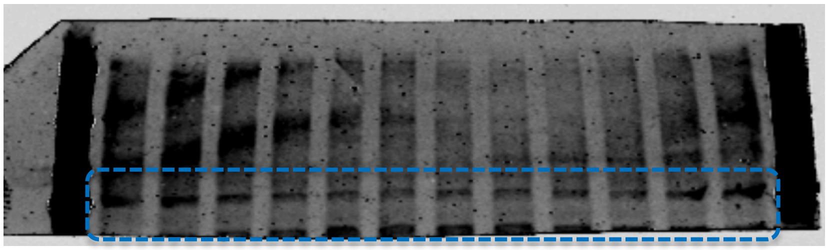

250KDa
150KDa
100KDa

stripped

cropped for figure 7A
quantified in figure 7D

4-20% Acrylamide
Scr si n=6
Diaph1 si n=6

Exp. Code: 068-2 Diaph1 KD in Hepa 1-6 cells

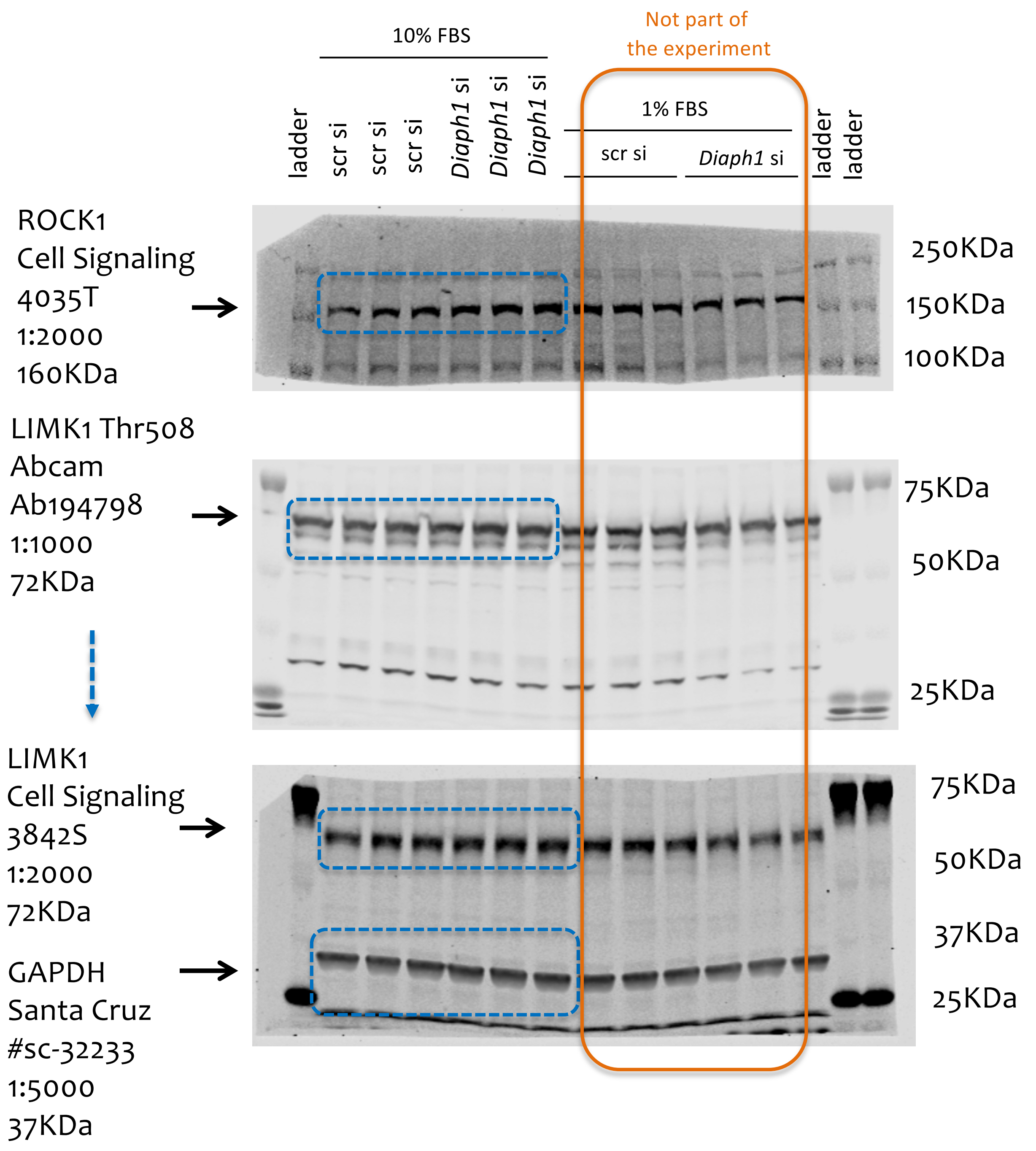

stripped

cropped for figure 7A
quantified in figure 7E, 7F

7.5% Acrylamide
Scr si n=3
Diaph1 si n=3

Exp. Code: 067-2 Diaph1 KD in Hepa 1-6 cells

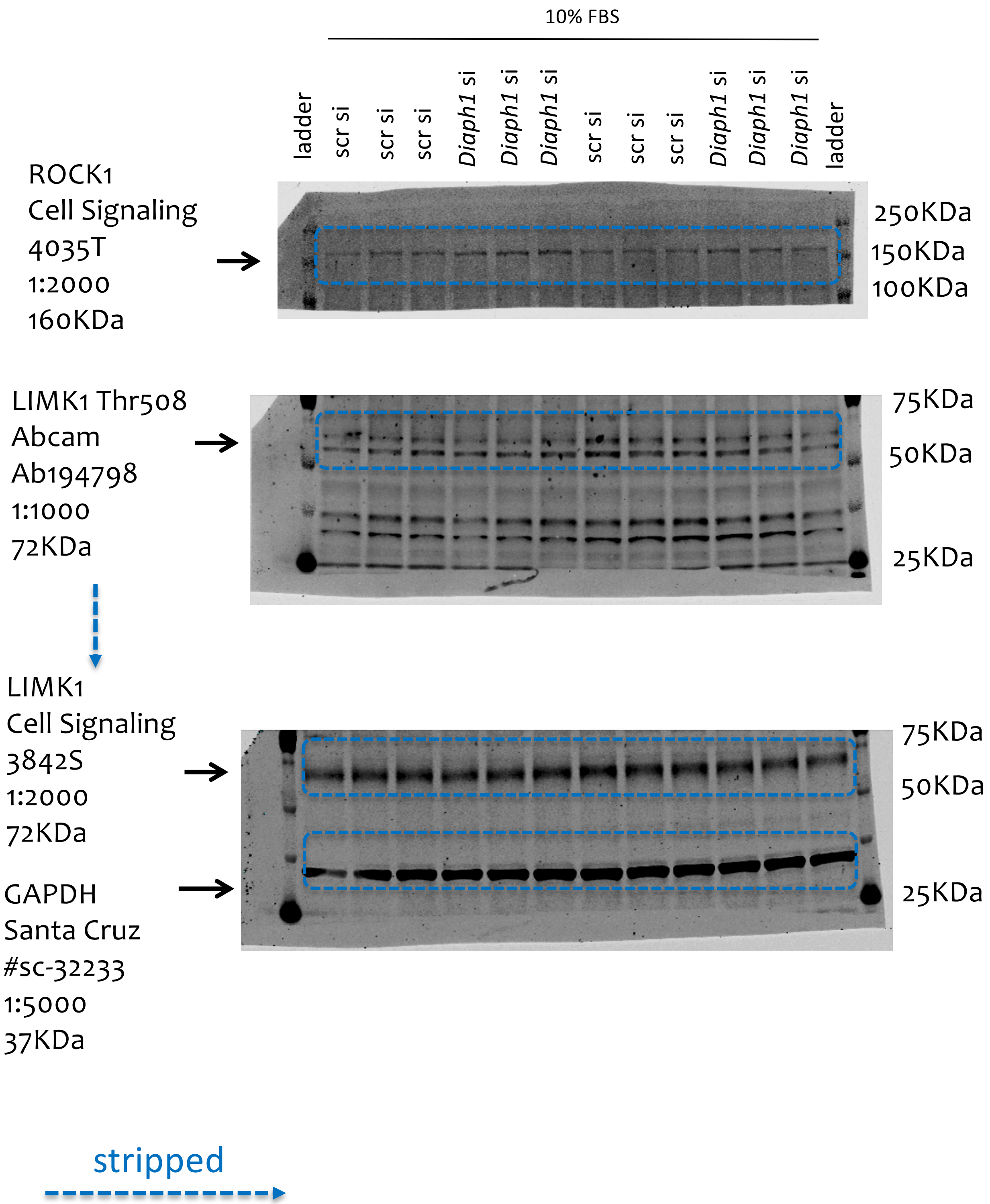

quantified in figure 7F

7.5% Acrylamide
Scr si n=6
Diaph1 si n=6

Figure 8A-D

Exp. Code: 086-1-FRACTIONS in HEPA 1-6

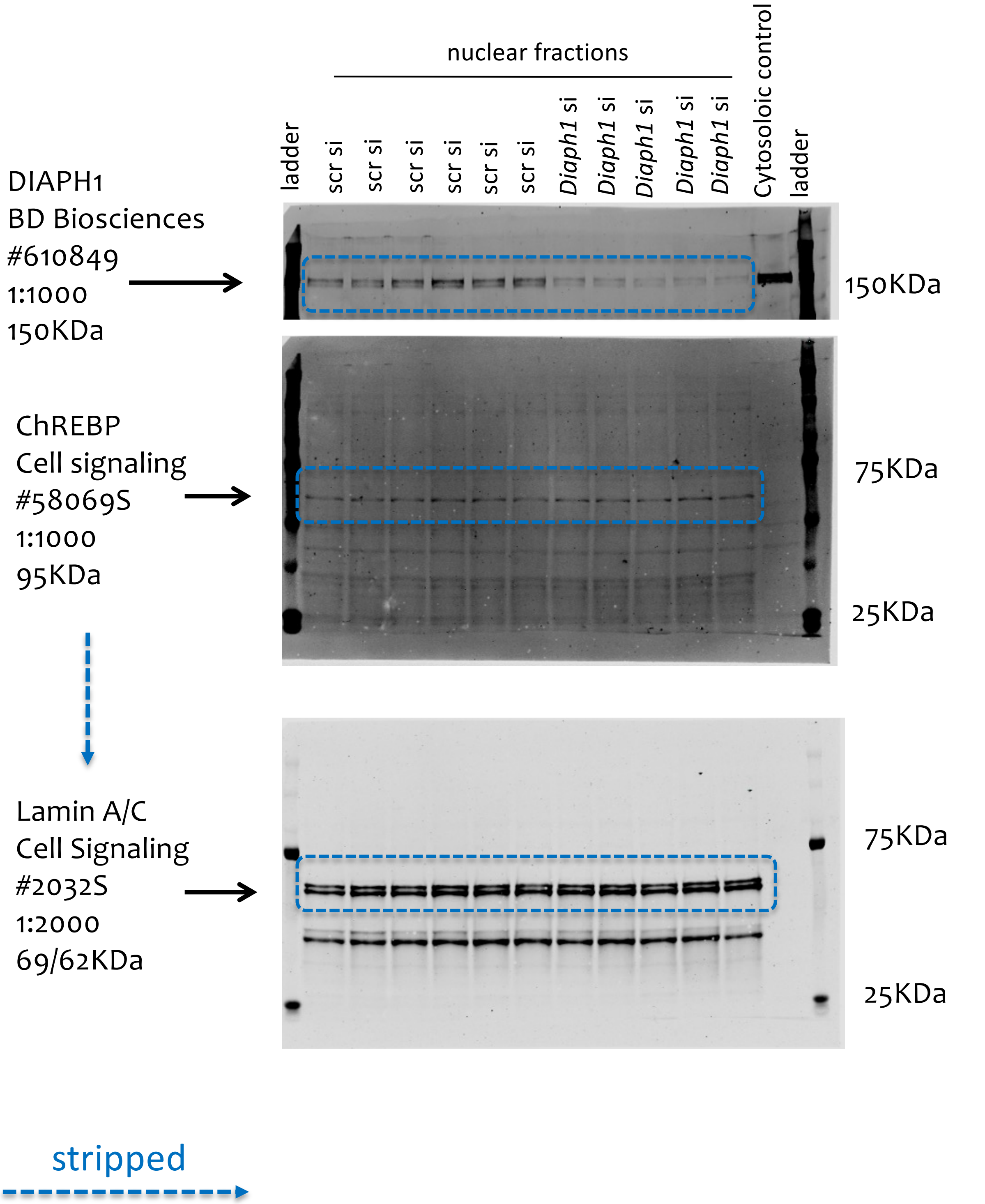

cropped for figure 8A
quantified in figure 8B

7.5% Acrylamide
Scr si nuclear n=6
Diaph1 si nuclear n=5

Exp. Code: 086-2-FRACTIONS in HEPA 1-6

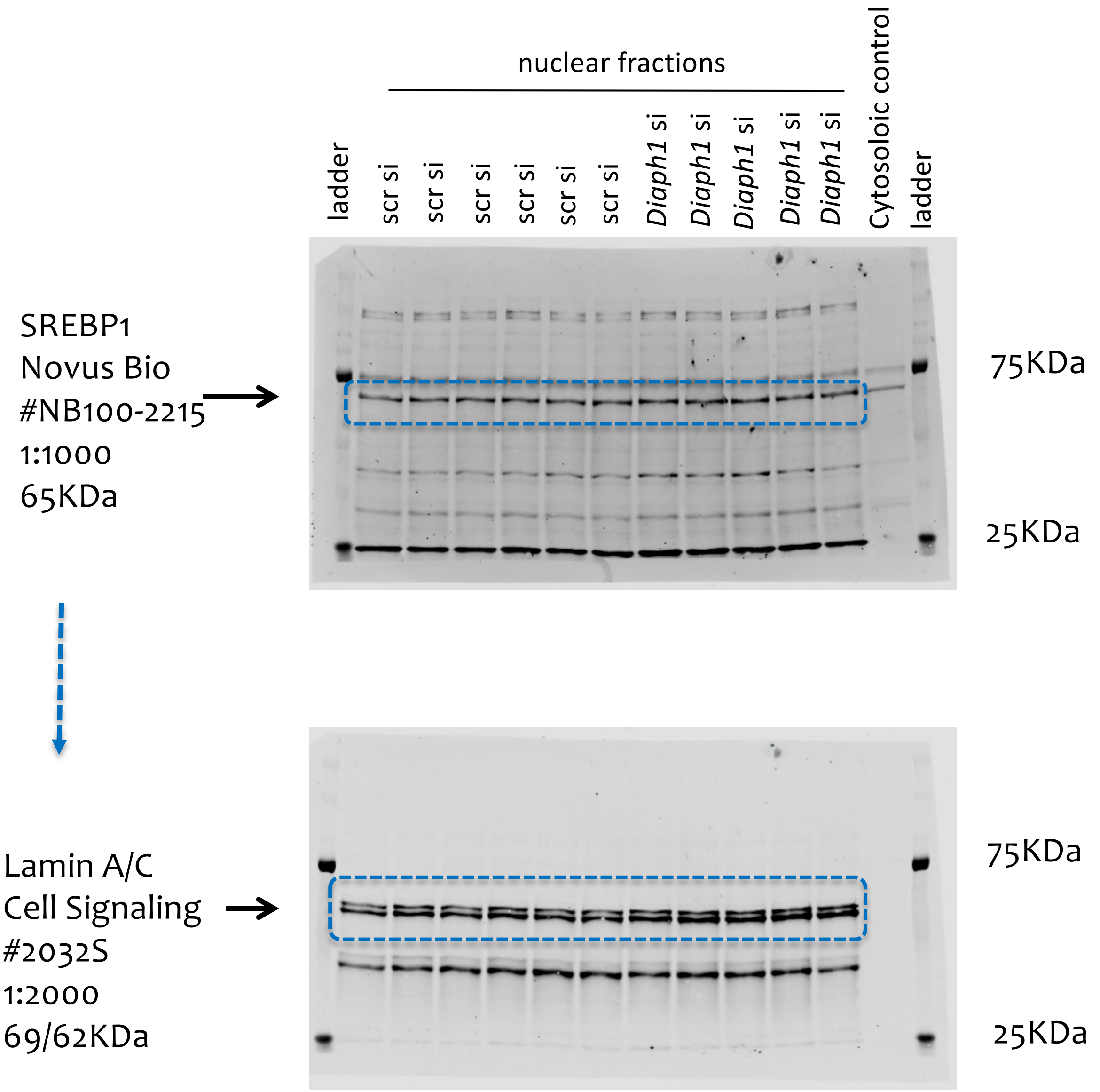

stripped

cropped for figure 8A
quantified in figure 8B

7.5% Acrylamide
Scr si nuclear n=6
Diaph1 si nuclear n=5

Supplemental Figure 7A-B-C

Exp. Code: 069-1-FRACTIONATION in livers

cytosolic fractions

ladder | Ldlr-/- | Ldlr-/- | Ldlr-/- | Ldlr-/- | Ldlr-/- | Ldlr-/- | Ldlr-/-Diaph1-/- | Ldlr-/-Diaph1-/- | Ldlr-/-Diaph1-/- | Ldlr-/-Diaph1-/- | Ldlr-/-Diaph1-/- | Ldlr-/-Diaph1-/- | Nuclear control | ladder

DIAPH1
BD Biosciences
#610849
1:1000
150KDa

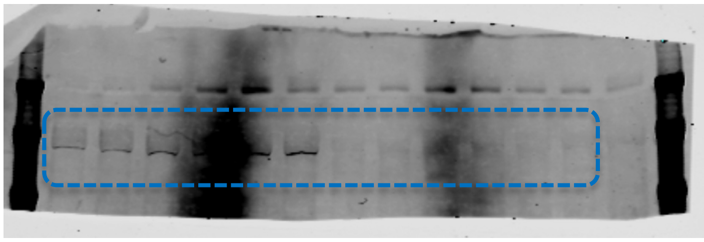

250KDa
150KDa
100KDa

Lamin A/C
Cell Signaling
#2032S
1:2000
69/62KDa

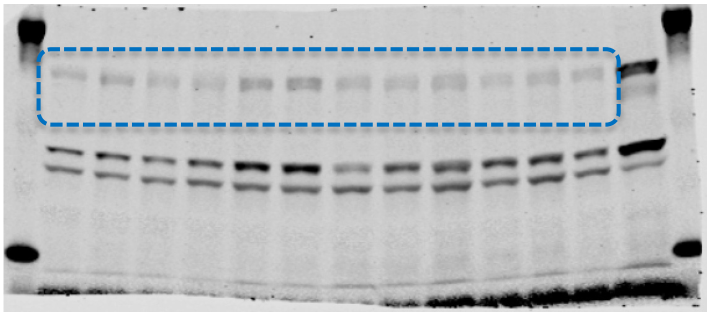

75KDa
50KDa
25KDa

GAPDH
Santa Cruz
#sc-32233
1:5000
37KDa

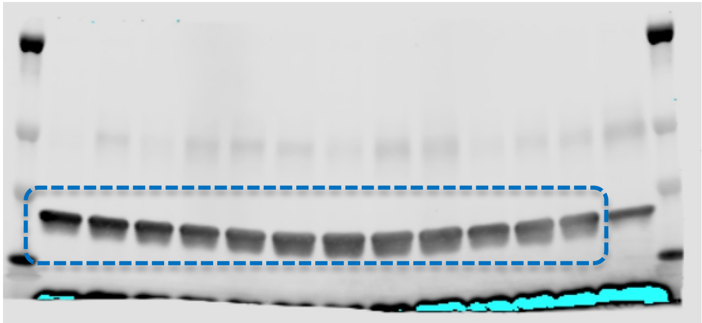

75KDa
25KDa

cropped for Supp figure 7A
quantified in Supp figure 7B

7.5 % Acrylamide
Ldlr-/- cytosolic n=6
Ldlr-/-Diaph1-/- cytosolic =6

Exp. Code: 069-5-FRACTIONATION in livers

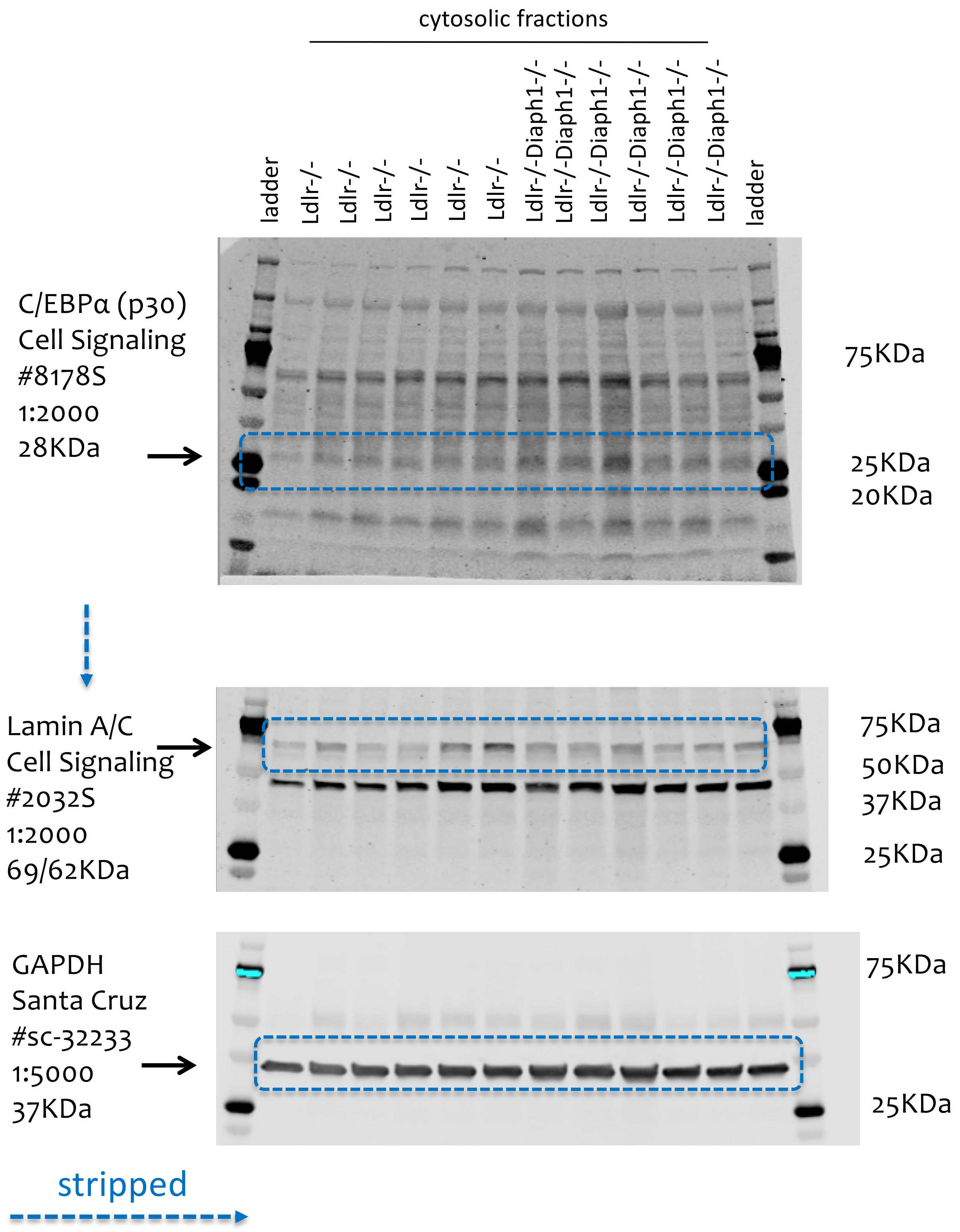

cropped for Supp figure 7A
quantified in Supp figure 7B

4-20% Acrylamide
Ldlr-/- cytosolic n=6
Ldlr-/-Diaph1-/- cytosolic =6

Exp. Code: 069-3-FRACTIONATION in livers

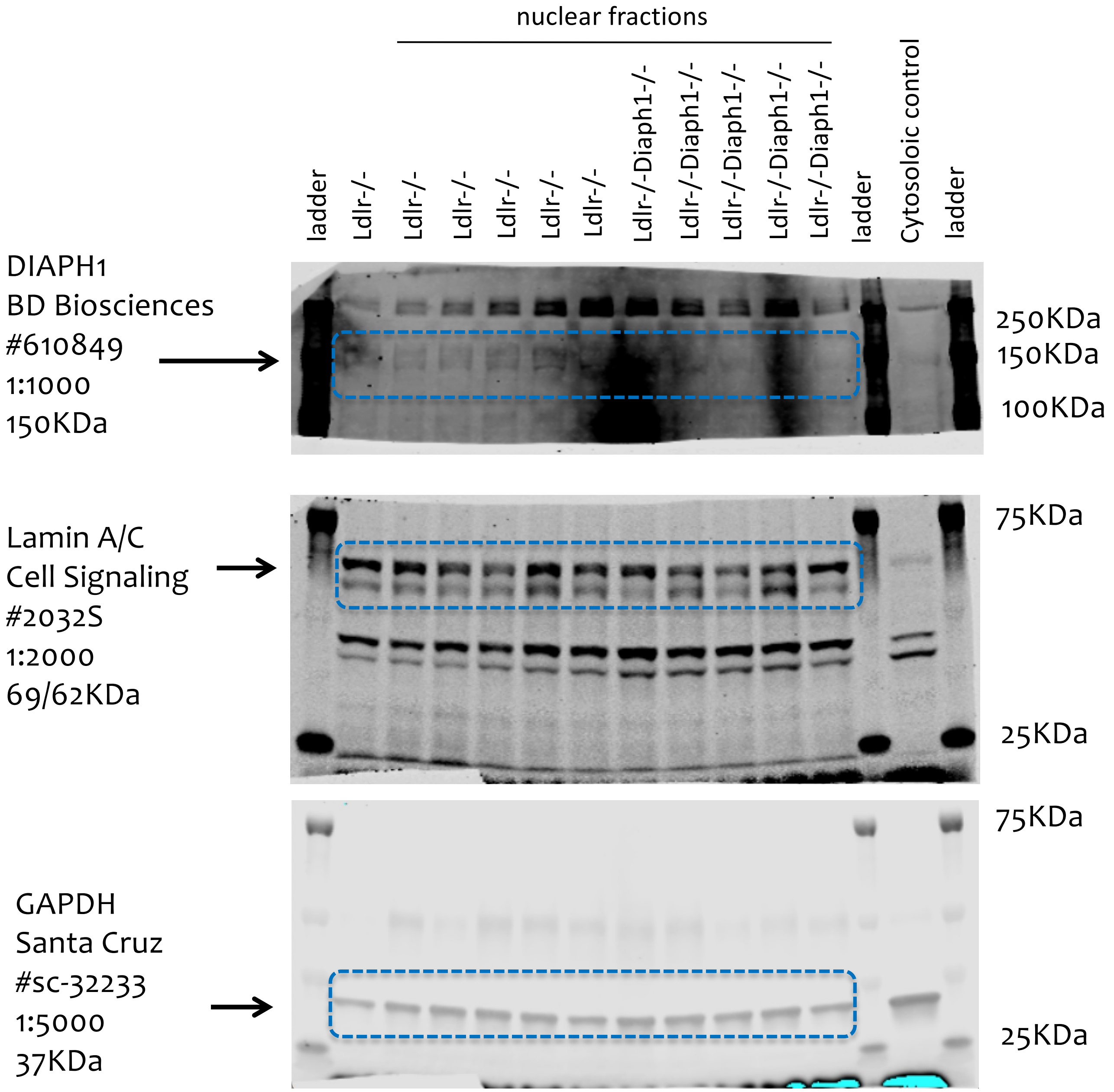

cropped for Supp figure 7A
quantified in Supp figure 7C

7.5% Acrylamide
Ldlr-/- nuclear n=6
Ldlr-/-Diaph1-/- nuclear n=5

Exp. Code: 069-6-FRACTIONATION in livers

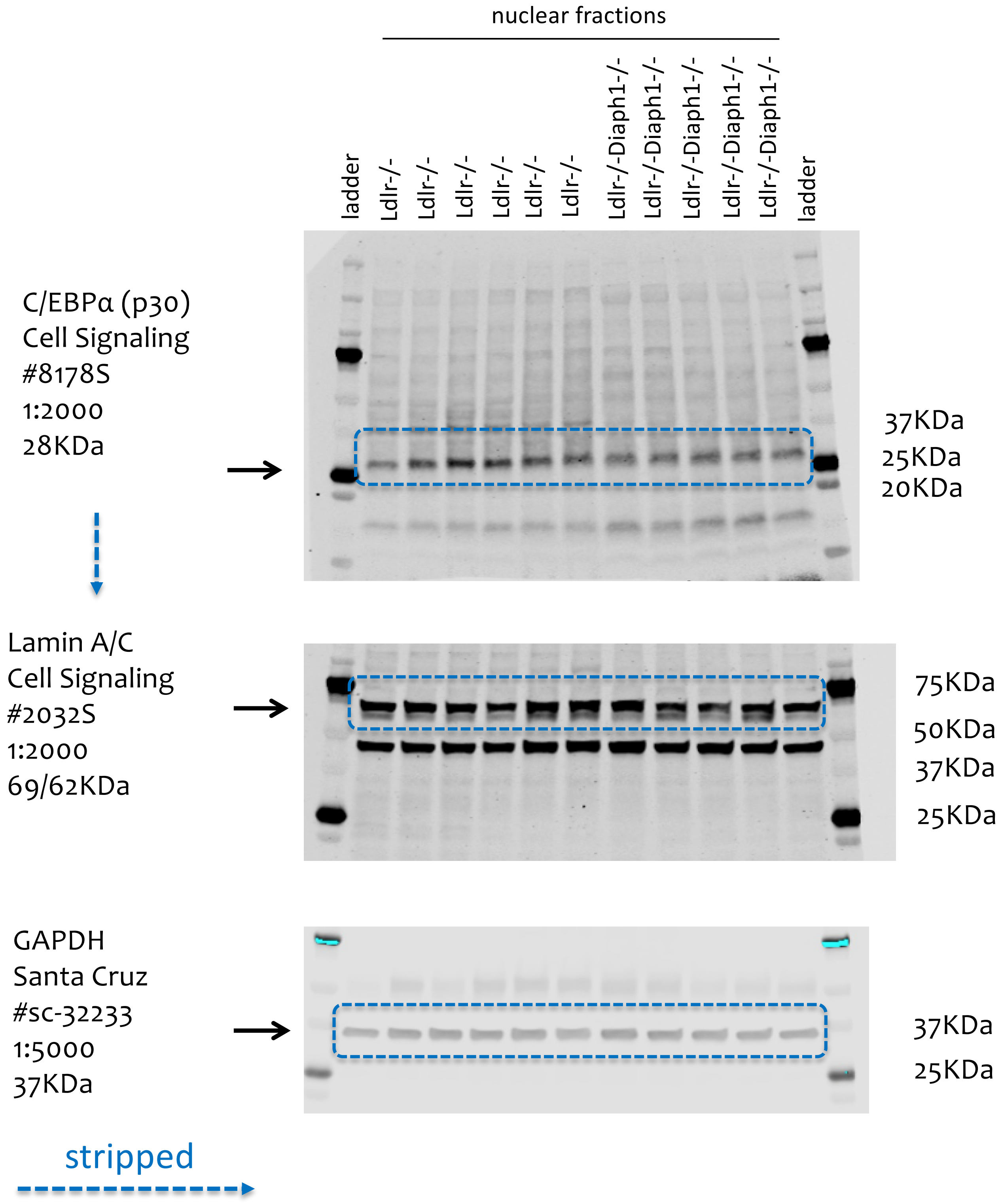

cropped for Supp figure 7A
quantified in Supp figure 7C

4-20% Acrylamide
Ldlr-/- nuclear n=6
Ldlr-/-Diaph1-/- nuclear n=5

Supplemental Figure 9A-B-C-D-E

Exp. Code: 065-9-TOTAL LYSATE in livers

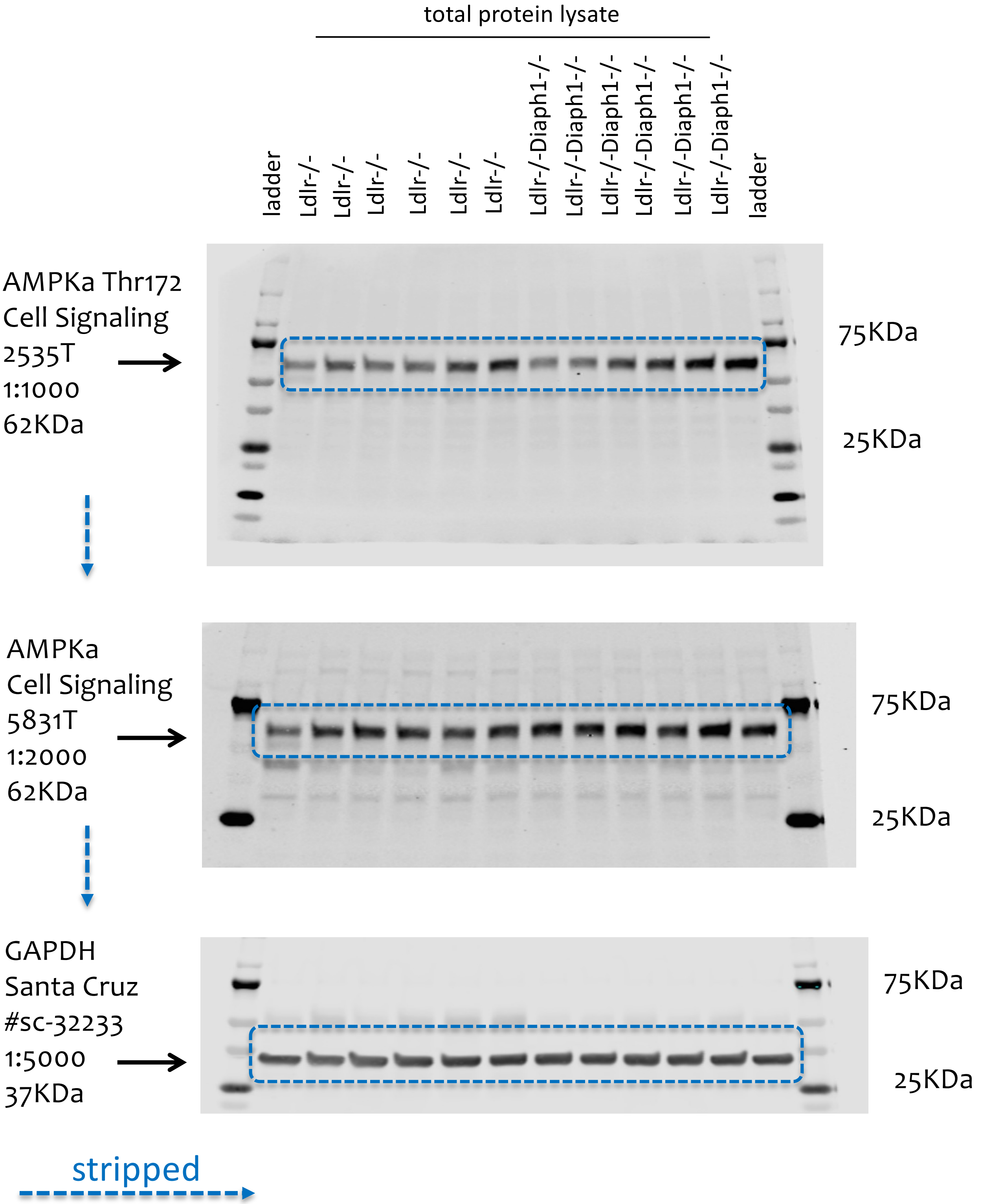

cropped for Supp figure 9A
quantified in Supp figure 9B

4-20% Acrylamide
Ldlr-/- n=6
Ldlr-/-Diaph1-/- n=6

Exp. Code: 065-5-TOTAL LYSATE in livers

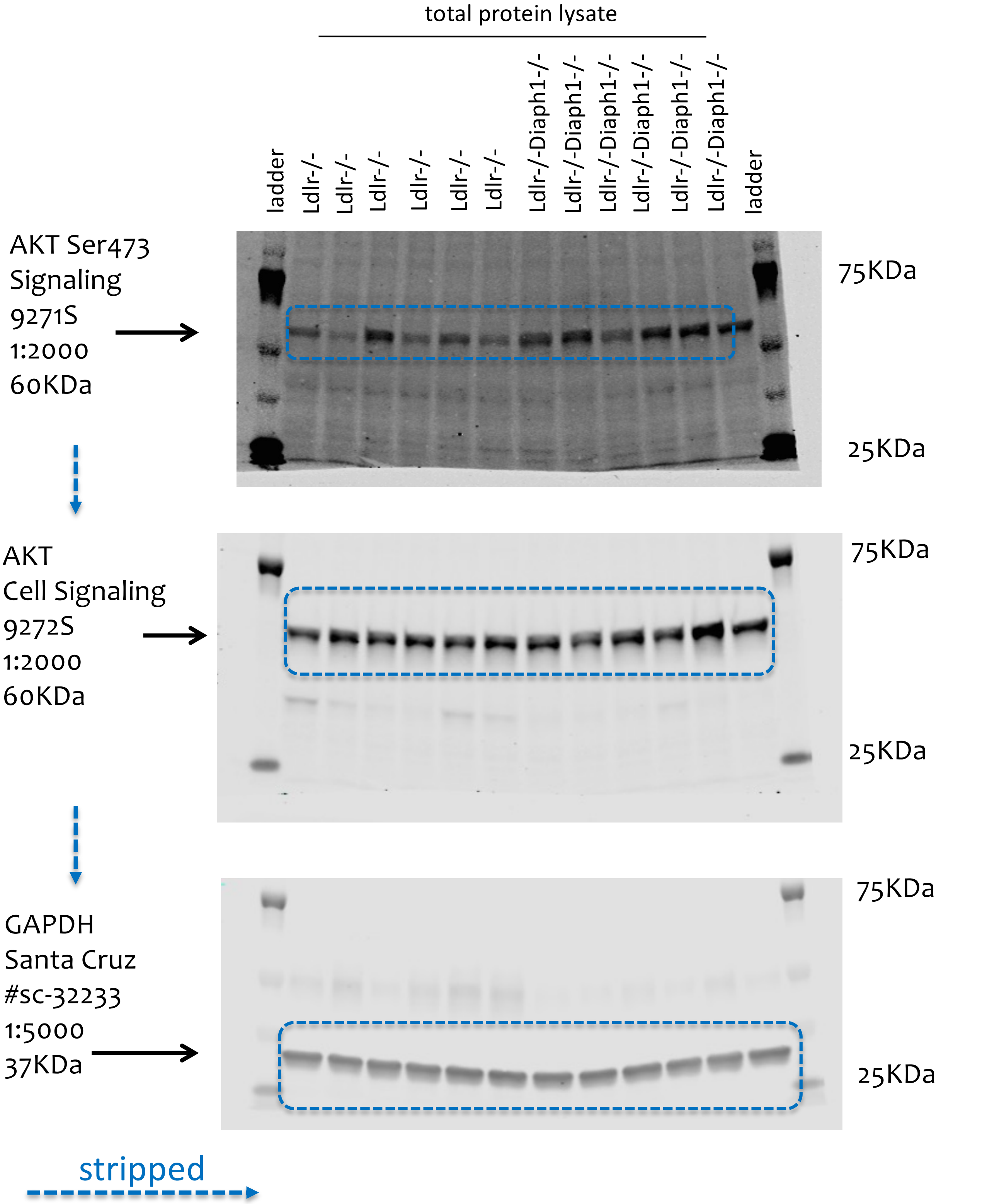

cropped for Supp figure 9A
quantified in Supp figure 9C

7.5% Acrylamide
Ldlr-/- n=6
Ldlr-/-Diaph1-/- n=6

Exp. Code: 065-7-TOTAL LYSATE in livers

total protein lysate

ladder | Ldlr-/- | Ldlr-/- | Ldlr-/- | Ldlr-/- | Ldlr-/- | Ldlr-/- | Ldlr-/-Diaph1-/- | Ldlr-/-Diaph1-/- | Ldlr-/-Diaph1-/- | Ldlr-/-Diaph1-/- | Ldlr-/-Diaph1-/- | Ldlr-/-Diaph1-/- | ladder

mTOR Ser2448
Cell Signaling
5536T
1:2000
289KDa

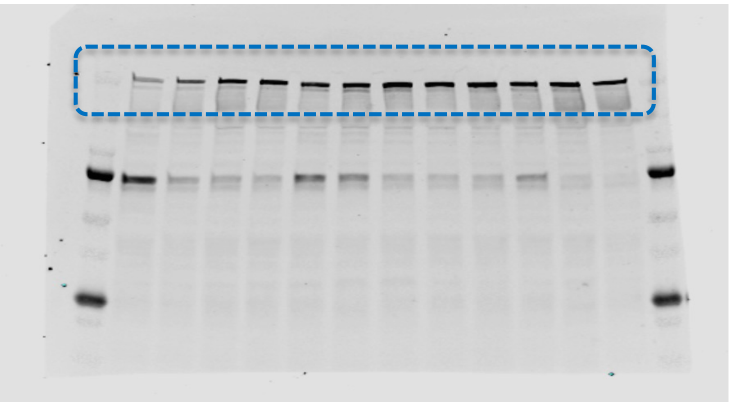

250KDa
75KDa
25KDa

mTOR
Cell Signaling
2972S
1:2000
289KDa

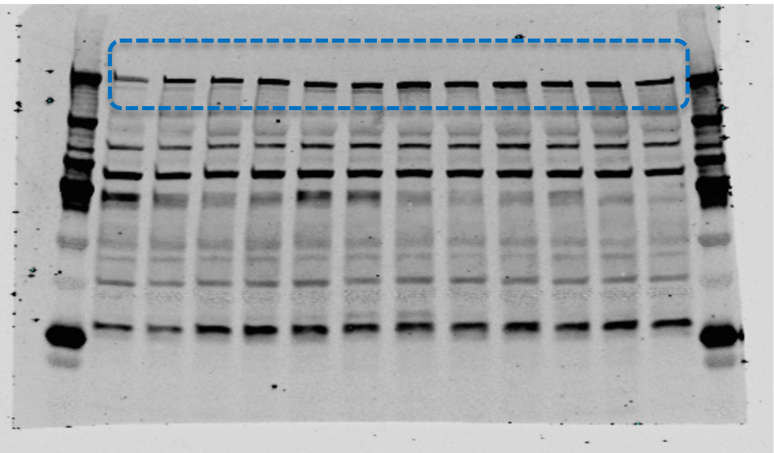

250KDa
75KDa
25KDa

GAPDH
Santa Cruz
#sc-32233
1:5000
37KDa

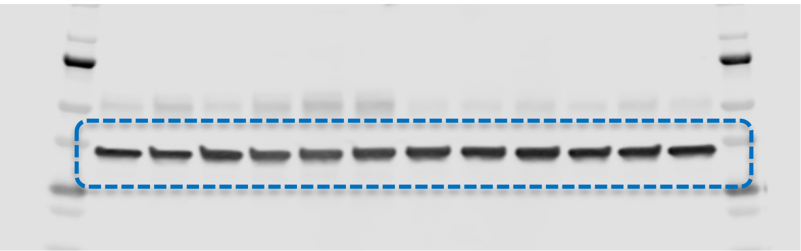

75KDa
25KDa

stripped

cropped for Supp figure 9A
quantified in Supp figure 9D

4-20% Acrylamide
Ldlr-/- n=6
Ldlr-/-Diaph1-/- n=6

Exp. Code: 065-8-TOTAL LYSATE in livers

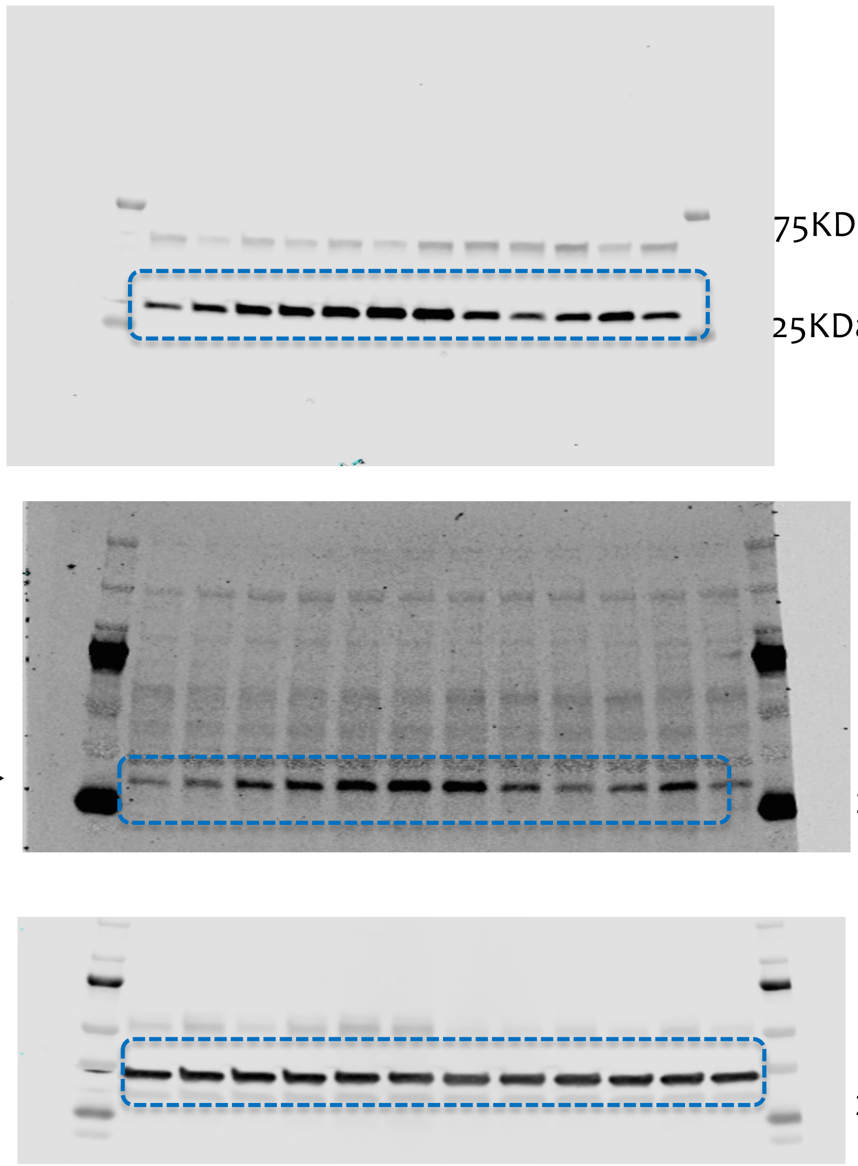

cropped for Supp figure 9A
quantified in Supp figure 9E

4-20% Acrylamide
Ldlr-/- n=6
Ldlr-/-Diaph1-/- n=6

Supplemental Figure 10A-B-C

Exp. Code: 076-1-TOTAL LYSATE in Aortas

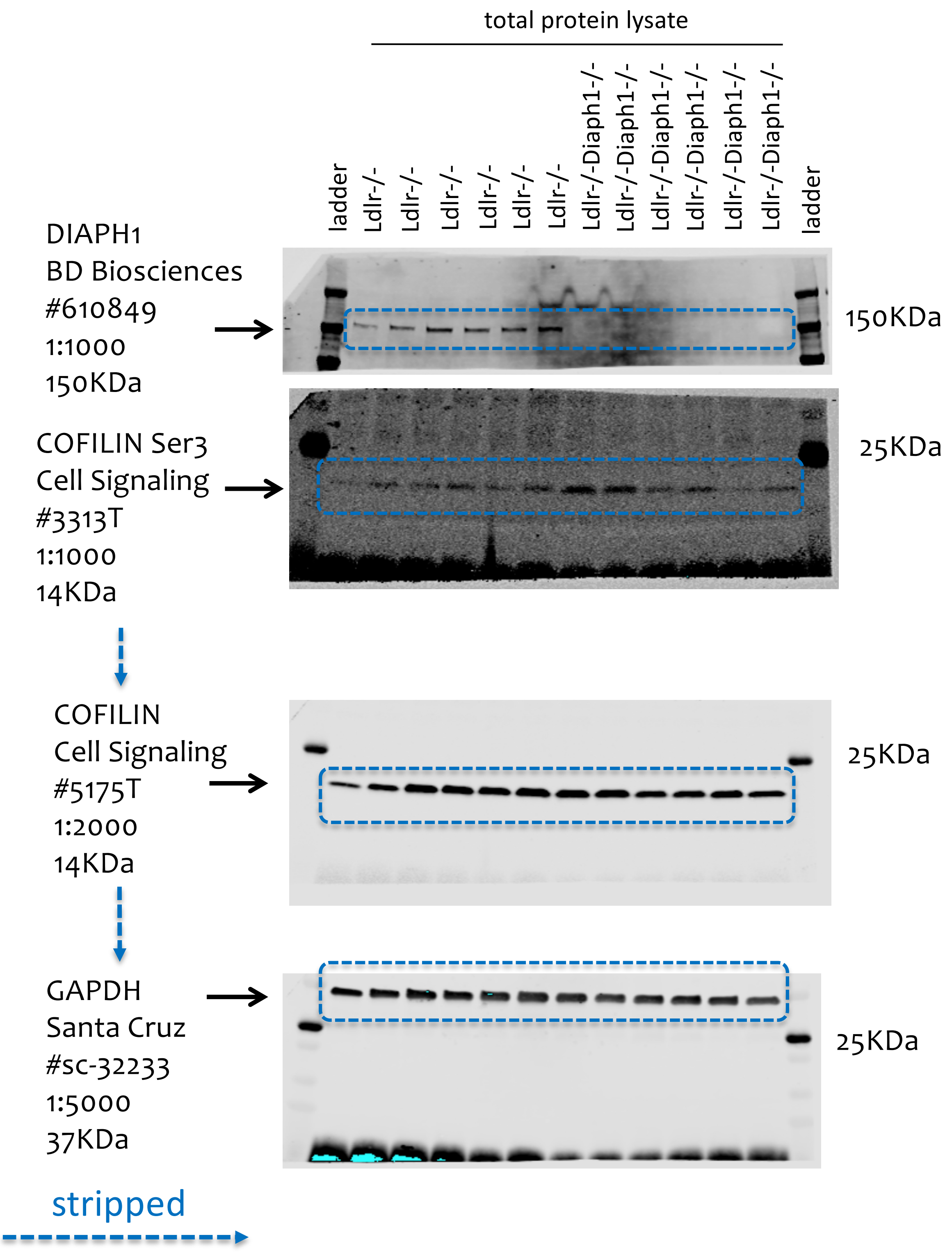

cropped for Supp figure 10A
quantified in Supp figure 10B, C

4-20% Acrylamide
Ldlr-/- n=6
Ldlr-/-Diaph1-/- n=6
