# Supplementary material for: DIAPH1 mediates progression of atherosclerosis and regulates hepatic lipid metabolism in mice
Source: Commun Biol. 2023 Mar 17;6:280. doi: 10.1038/s42003-023-04643-2 (PMC10023694; doi:10.1038/s42003-023-04643-2)

Figure 5D

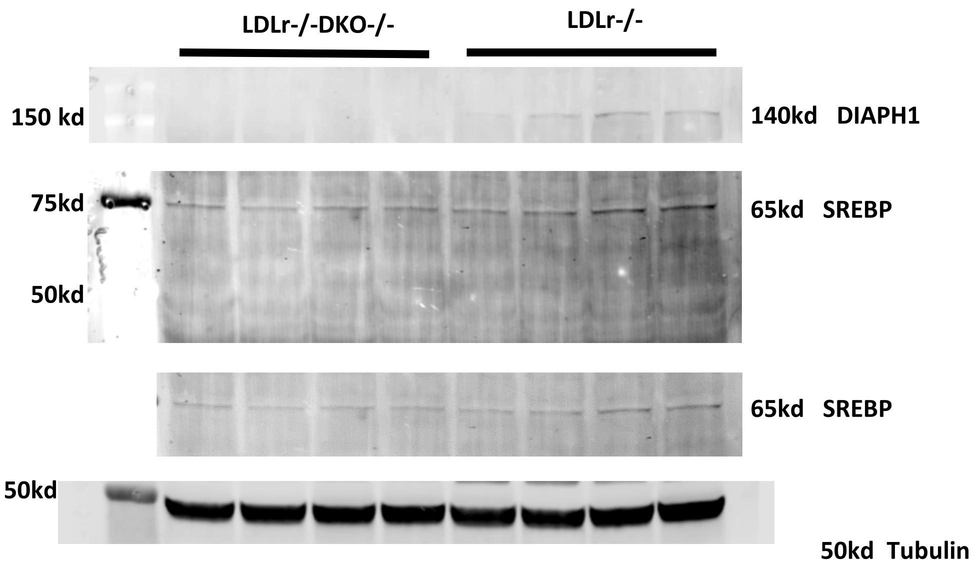

For publication use below, the image has been flipped

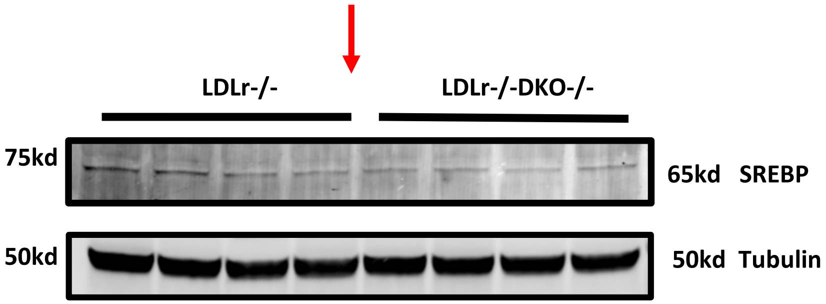

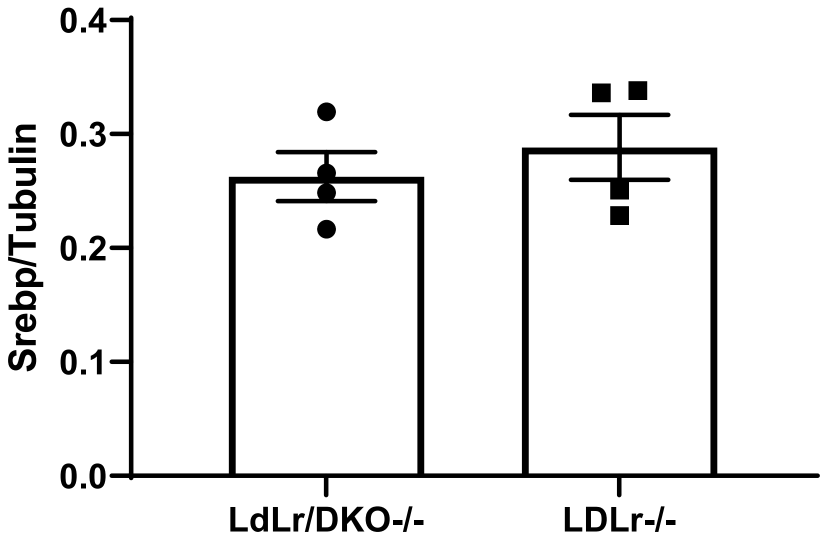

Column B	LDLr-/-
vs.	vs.
Column A	LdLr/DKO-/-
Unpaired t test	
P value	0.4979
P value summary	ns
Significantly different (P < 0.05)?	No
One- or two-tailed P value?	Two-tailed
t, df	t=0.7212, df=6
How big is the difference?	
Mean of column A	0.2626
Mean of column B	0.2883
Difference between means (B - A) ± SEM	0.02576 ± 0.03571
95% confidence interval	-0.06163 to 0.1131
R squared (eta squared)	0.07977
F test to compare variances	
F, DFn, Dfd	1.757, 3, 3
P value	0.6547
P value summary	ns
Significantly different (P < 0.05)?	No
Data analyzed	
Sample size, column A	4
Sample size, column B	4

Figure 5D

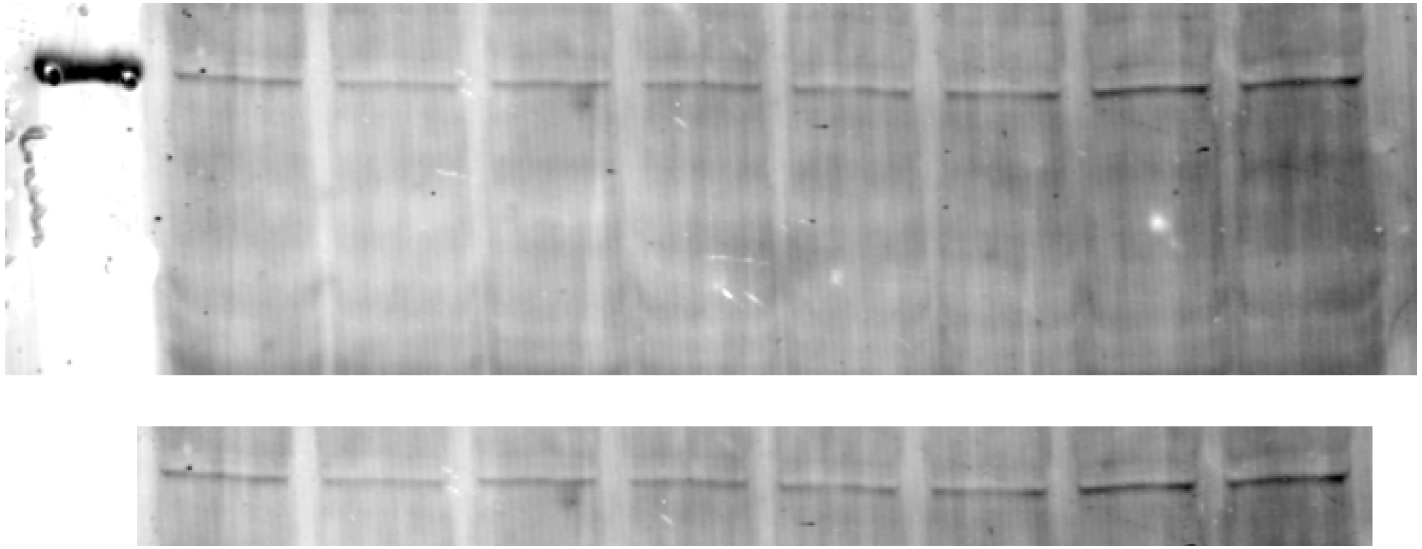

Supplement: Supplementary file 6 — Supplementary Data 4 [file 42003_2023_4643_MOESM6_ESM.pdf]
