# Supplementary figures and images for: DIAPH1 mediates progression of atherosclerosis and regulates hepatic lipid metabolism in mice
Source: Commun Biol. 2023 Mar 17;6:280. doi: 10.1038/s42003-023-04643-2 (PMC10023694; doi:10.1038/s42003-023-04643-2)

**Figure 8C**

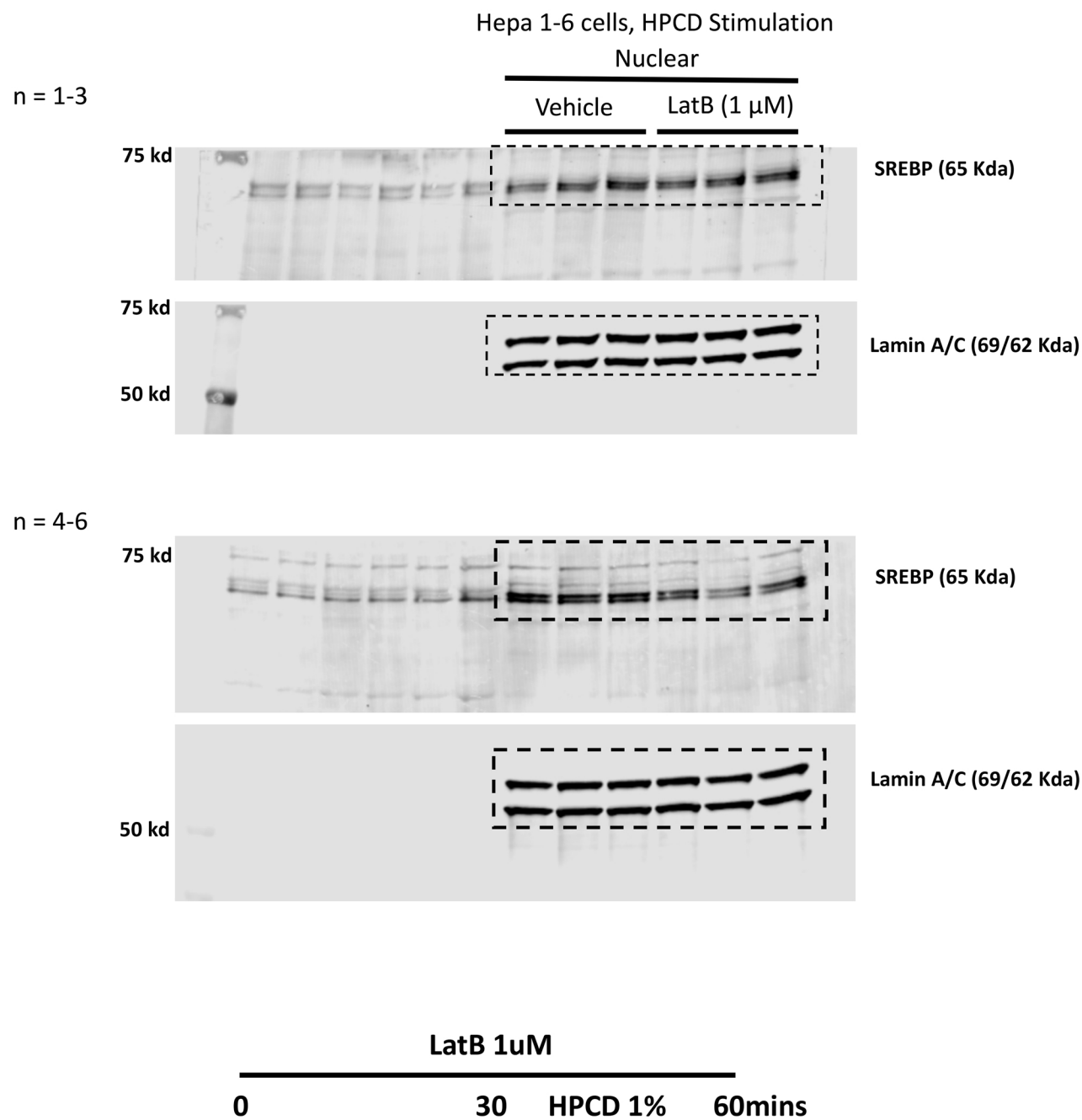

Supplement: Supplementary file 7 — Supplementary Data 5 [file 42003_2023_4643_MOESM7_ESM.pdf]
