# Supplementary material for: DIAPH1 mediates progression of atherosclerosis and regulates hepatic lipid metabolism in mice
Source: Commun Biol. 2023 Mar 17;6:280. doi: 10.1038/s42003-023-04643-2 (PMC10023694; doi:10.1038/s42003-023-04643-2)
Supplement: Supplementary file 9 — Reporting Summary [file 42003_2023_4643_MOESM9_ESM.pdf]

nature portfolio

Corresponding author(s): Ann Marie Schmidt

Last updated by author(s): Feb 8, 2023

Reporting Summary

Nature Portfolio wishes to improve the reproducibility of the work that we publish. This form provides structure for consistency and transparency in reporting. For further information on Nature Portfolio policies, see our Editorial Policies and the Editorial Policy Checklist.

Statistics

For all statistical analyses, confirm that the following items are present in the figure legend, table legend, main text, or Methods section.

n/a	Confirmed	
☐	☒	The exact sample size (n) for each experimental group/condition, given as a discrete number and unit of measurement
☐	☒	A statement on whether measurements were taken from distinct samples or whether the same sample was measured repeatedly
☐	☒	The statistical test(s) used AND whether they are one- or two-sided<br>*Only common tests should be described solely by name; describe more complex techniques in the Methods section.*
☐	☒	A description of all covariates tested
☐	☒	A description of any assumptions or corrections, such as tests of normality and adjustment for multiple comparisons
☐	☒	A full description of the statistical parameters including central tendency (e.g. means) or other basic estimates (e.g. regression coefficient) AND variation (e.g. standard deviation) or associated estimates of uncertainty (e.g. confidence intervals)
☐	☒	For null hypothesis testing, the test statistic (e.g. F, t, r) with confidence intervals, effect sizes, degrees of freedom and P value noted<br>*Give P values as exact values whenever suitable.*
☒	☐	For Bayesian analysis, information on the choice of priors and Markov chain Monte Carlo settings
☒	☐	For hierarchical and complex designs, identification of the appropriate level for tests and full reporting of outcomes
☐	☒	Estimates of effect sizes (e.g. Cohen's d, Pearson's r), indicating how they were calculated

Our web collection on statistics for biologists contains articles on many of the points above.

Software and code

Policy information about availability of computer code

Data collection	Odyssey (Li-COR) Infrared Imaging System was used to collect the western blots images<br>LSRII UV (BD Biosciences) cell analyzer and BD FACSDiva software was used to collect the flow cytometry data
Data analysis	Histological Images were analyzed using Fiji distribution of ImageJ 2.0.0. and Image Pro Plus 7.0<br>Flow cytometry data was analyzed using FlowJo 10.8.1<br>Statistical analysis and graphs were generated using Graph Pad Prism 9.<br>RNASeq Analysis was performed as follows.<br>Fastq files were aligned to the mouse genome with Rsubread.<br>Gene expression was quantified with featureCounts.<br>Differential expression analysis was performed with Limma 3.38.3<br>Pathway Analysis was performed with iPathwayguide and WebGestalt<br>Hierarchical clustering was performed using Cluster 3.0.<br>Dendrograms and heatmaps were displayed using JavaTreeview.

For manuscripts utilizing custom algorithms or software that are central to the research but not yet described in published literature, software must be made available to editors and reviewers. We strongly encourage code deposition in a community repository (e.g. GitHub). See the Nature Portfolio guidelines for submitting code & software for further information.

Data

Policy information about availability of data

All manuscripts must include a data availability statement. This statement should provide the following information, where applicable:

- Accession codes, unique identifiers, or web links for publicly available datasets
- A description of any restrictions on data availability
- For clinical datasets or third party data, please ensure that the statement adheres to our policy

For RNA Seq murine raw (FASTQ) and aligned (BAM) sequencing data, resulting raw and normalized count data. RNA seq data is publicly available through NCBI GEO accession GSE156403

Human research participants

Policy information about studies involving human research participants and Sex and Gender in Research.

Reporting on sex and gender	1). Deidentified human coronary artery atherosclerotic specimens with advanced lesions were obtained from CVPath Institute Sudden Death Registry. The study was approved by the CVPath Institutional Review Board (IRB) as an exempt study (#RP0063).<br>2). Deidentified normal human liver specimen (52 year-old male subject) was obtained from the Center for Biospecimen and Research Development at the NYU Grossman School of Medicine. The study was approved by the NYU Grossman School of Medicine Institutional Review Board (IRB), study number s16-00122.
Population characteristics	*Describe the covariate-relevant population characteristics of the human research participants (e.g. age, genotypic information, past and current diagnosis and treatment categories). If you filled out the behavioural & social sciences study design questions and have nothing to add here, write "See above."*
Recruitment	*Describe how participants were recruited. Outline any potential self-selection bias or other biases that may be present and how these are likely to impact results.*
Ethics oversight	1). Coronary artery atherosclerotic specimens: CVPath Institutional Review Board (IRB) as an exempt study (#RP0063)<br>2). Normal human liver: NYU Grossman School of Medicine Institutional Review Board (IRB), study number s16-00122

Note that full information on the approval of the study protocol must also be provided in the manuscript.

Field-specific reporting

Please select the one below that is the best fit for your research. If you are not sure, read the appropriate sections before making your selection.

☒ Life sciences ☐ Behavioural & social sciences ☐ Ecological, evolutionary & environmental sciences

For a reference copy of the document with all sections, see nature.com/documents/nr-reporting-summary-flat.pdf

Life sciences study design

All studies must disclose on these points even when the disclosure is negative.

Sample size	Sample size was determined based on previously published works from both our lab and others
Data exclusions	No data were excluded from this manuscript.
Replication	All analyses used biological replication and several key findings were verified using a distinct approach. Biological replicates are noted in each figure legend.
Randomization	Mice were randomly assigned to each group. Littermates from the same 10 breeding pairs per genotype were selected for use in this study.
Blinding	For immunohistochemistry experiments, the experimenter was naive to the experimental code or mouse tag number during quantification only. Blinding of the RNA seq data and western blot data was not possible as knowledge of group identity is required for the loading procedure and analysis. Blinding is not necessary since the methods used are objective.

Reporting for specific materials, systems and methods

We require information from authors about some types of materials, experimental systems and methods used in many studies. Here, indicate whether each material, system or method listed is relevant to your study. If you are not sure if a list item applies to your research, read the appropriate section before selecting a response.

Materials & experimental systems

n/a	Involved in the study
☐	☒ Antibodies
☐	☒ Eukaryotic cell lines
☒	☐ Palaeontology and archaeology
☐	☒ Animals and other organisms
☒	☐ Clinical data
☒	☐ Dual use research of concern

Methods

n/a	Involved in the study
☒	☐ ChIP-seq
☐	☒ Flow cytometry
☒	☐ MRI-based neuroimaging

Antibodies

Antibodies used

WB: Primary Antibodies:
Mouse monoclonal mDia1 (DIAPH1) (610849, BD Biosciences) (1:1000 for WB)
Mouse monoclonal SREBP-1 (sc-13551, Santa Cruz) (1:2000 for WB)
Rabbit polyclonal SREBP-1 (NB100-2215, Novus Biologicals) (1:1000 for WB)
Mouse monoclonal SREBP-2 (sc-13552, Santa Cruz) (1:2000 for WB)
Rabbit polyclonal ChREBP (NB400-135, Novus Biologicals) (1:2000 for WB)
Rabbit polyclonal ChREBP (58069S, Cell Signaling) (1:1000 for WB)
Rabbit polyclonal Lamin A/C (2032S, Cell Signaling) (1:2000 for WB)
Rabbit polyclonal AKT Ser473 (9271S, Cell Signaling) (1:2000 for WB)
Rabbit polyclonal AKT (9272S, Cell Signaling) (1:2000 for WB)
Rabbit polyclonal AMPKα Thr172 (2535T, Cell Signaling) (1:2000 for WB)
Rabbit polyclonal AMPKα (5831T, Cell Signaling) (1:2000 for WB)
Rabbit polyclonal mTOR Ser2448 (5536T, Cell Signaling) (1:2000 for WB)
Rabbit polyclonal mTOR (2972S, Cell Signaling) (1:2000 for WB)
Rabbit polyclonal S6 Ser240/244 (2215S, Cell Signaling) (1:2000 for WB)
Rabbit polyclonal S6 (2317S, Cell Signaling) (1:2000 for WB)
Rabbit polyclonal ROCK1 (4035T, Cell Signaling) (1:2000 for WB)
Rabbit polyclonal LIMK1 Thr508 (Ab194798, Abcam) (1:1000 for WB)
Rabbit polyclonal LIMK1 (3842S, Cell Signaling) (1:2000 for WB)
Rabbit polyclonal SSH1L Ser978 (SP3901, ECM Biosciences) (1:1000 for WB)
Rabbit polyclonal SSH1L (SP1711, ECM Biosciences) (1:1000 for WB)
Rabbit polyclonal COFILIN Ser3 (3313T, Cell Signaling) (1:1000 for WB)
Rabbit polyclonal COFILIN (5175T, Cell Signaling) (1:2000 for WB)
Rabbit polyclonal C/EBPα (8178S, Cell Signaling) (1:2000 for WB)
Mouse monoclonal GAPDH (sc-32233, Santa Cruz) (1:5000 for WB)

Secondary Antibodies:
Goat polyclonal IRDye 680RD anti-mouse (925-68070, C90910-20, Li-Cor) (1:5000 for WB)
Goat polyclonal IRDye 800RD anti-rabbit (925-32211, C90910-ll, Li-Cor) (1:5000 for WB)

IHC
Primary antibodies (for murine tissues):
Rabbit polyclonal DIAPH1 (ab11173, Abcam) (1:200 dilution for IHC)
Rat monoclonal CD68 (MCA1957, Biorad) (1:200 dilution for IHC)
Goat polyclonal α-Smooth Muscle Actin (SMA) (PA5-18292, ThermoFisher Scientific) (1:200 dilution for IHC)
Goat polyclonal RAGE (GTX27764, Genetex) (1:150 dilution for IHC)
Rabbit polyclonal AGE (ab23722, Abcam) (1:200 dilution for IHC)
Rabbit monoclonal DIAPH1 (ab129167, clone EPR7948, Abcam) (1:200 dilution for IHC)

Secondary antibodies (for murine tissues):
Goat polyclonal Alexa Fluor 488 anti-rabbit (A11008, ThermoFisher Scientific) (1:200 dilution for IHC)
Donkey polyclonal Alexa Fluor 555 anti-rat (A48270, ThermoFisher Scientific) (1:200 dilution for IHC)
Donkey polyclonal Alexa Fluor 555 anti-goat (A32816, ThermoFisher Scientific) (1:200 dilution for IHC)
Donkey polyclonal Alexa Fluor 594 anti-goat (A11058, Thermo Fisher Scientific) (1:200 dilution for IHC)
Goat polyclonal biotinylated anti-rabbit (B-2770, ThermoFisher Scientific) (1:100 dilution for IHC)

Primary antibodies (for human tissues):
Rabbit polyclonal DIAPH1 (ab11173, Abcam) (1:200 dilution for IHC)
Mouse monoclonal CD68 (M0814, Dako) (1:200 dilution for IHC)
Mouse monoclonal αSMA (A2547, Millipore Sigma) (1:500 dilution for IHC)
Rabbit monoclonal DIAPH1 (ab129167, clone EPR7948, Abcam) (1:200 dilution for IHC)

Secondary antibodies (for human tissues):
Goat polyclonal Alexa Fluor 488 anti-rabbit (A-11034, ThermoFisher Scientific) (1:200 dilution for IHC)
Donkey polyclonal Alexa Fluor 594 anti-mouse (A-21203, ThermoFisher Scientific) (1:200 dilution for IHC)

Flow cytometry
Primary antibodies:
Rat monoclonal CD16/32 (101302, Biolegend) (1:25 dilution for FC)
Rat monoclonal CD45 - Alexa Fluor 700 (103128, Biolegend) (1:100 dilution for FC)
Rat monoclonal CD11b - APC-Cy7 (557657, BD Biosciences) (1:100 dilution for FC)
Rat monoclonal CD45R/B220 - FITC (103206, Biolegend) (1:100 dilution for FC)
Armenian Hamster monoclonal CD3ε - FITC (100306, Biolegend) (1:100 dilution for FC)
Rat monoclonal CD170 (Siglec-F) - FITC (155504, Biolegend) (1:100 dilution for FC)
Rat monoclonal LY-6G - FITC (127606, Biolegend) (1:100 dilution for FC)
Rat monoclonal CD206 - Brilliant Violet 650 (141723, Biolegend) (1:100 dilution for FC)
Rat monoclonal LY-6C - Brilliant Violet 510 (128033, Biolegend) (1:100 dilution for FC)
Rat monoclonal CD14 - Brilliant Violet 421 (123329, Biolegend) (1:100 dilution for FC)
Rat monoclonal CD163 - PE (12-1631-82, Invitrogen) (1:100 dilution for FC)

Validation

All the primary antibodies used in this study are commercially available and were used for the applications validated by the manufacturers. All IHC experiments included negative controls by omission of primary antibody. All flow cytometry experiments included negative and positive controls, single staining and fluorescence minus one (FMO) controls. Validation procedures are described on the websites of the manufacturers:
WB:
Anti-mDia1 (610849, BD Biosciences): Applications: WB, IHC, IP. Reactivity: mouse, human, rat.
Anti-SREBP-1 (sc-13551, Santa Cruz): Applications: WB, IP, IF, IHC. Reactivity: mouse, rat, human, hamster.
Anti-SREBP-1 (NB100-2215, Novus Biologicals): Applications: WB, IF, IHC. Reactivity: mouse, human, rat, porcine, bovine, hamster, chinese hamster, plant.
Anti-SREBP-2 (sc-13552, Santa Cruz): Applications: WB, IP, IF. Reactivity: mouse, human, rat.
Anti-ChREBP (NB400-135, Novus Biologicals): Applications: WB, ChIP, IF, IHC, IP. Reactivity: mouse, human, rat.
Anti-ChREBP (58069S, Cell Signaling): Applications: WB. Reactivity: mouse, human, rat.
Anti-Lamin A/C (2032S, Cell Signaling): Applications: WB, IHC. Reactivity: mouse, human, rat.
Anti-AKT Ser473 (9271S, Cell Signaling): Applications: WB, IP, IF, IHC, F. Reactivity: mouse, human, rat, hamster, monkey, drosophila, bovine, dog.
Anti-AKT (9272S, Cell Signaling): Applications: WB, IP, IF, IHC, F. Reactivity: mouse, human, rat, hamster, monkey, chicken, drosophila, bovine, dog, pig.
Anti-AMPKα Thr172 (2535T, Cell Signaling): Applications: WB, IP, IHC. Reactivity: mouse, human, rat, hamster, monkey, drosophila, saccharomyces.
Anti-AMPKα (5831T, Cell Signaling): Applications: WB, IP. Reactivity: mouse, human, rat, monkey, bovine.
Anti-mTOR Ser2448 (5536T, Cell Signaling): Applications: WB, IP, IF, IHC. Reactivity: mouse, human, rat, monkey.
Anti-mTOR (2972S, Cell Signaling): Applications: WB, IP. Reactivity: mouse, human, rat, monkey.
Anti-S6 Ser240/244 (2215S, Cell Signaling): Applications: WB, IP. Reactivity: mouse, human, rat, monkey, zebrafish.
Anti-S6 (2317S, Cell Signaling): Applications: WB, IF, IHC, F. Reactivity: mouse, human, rat, monkey, drosophila.
Anti-ROCK1 (4035T, Cell Signaling): Applications: WB, IP. Reactivity: mouse, human, rat, monkey.
Anti-LIMK1 Thr508 (Ab194798, Abcam): Applications: WB, IF, IHC. Reactivity: Mouse, Human.
Anti-LIMK1 (3842S, Cell Signaling): Applications: WB, IP. Reactivity: mouse, human, rat, monkey.
Anti-SSH1L Ser978 (SP3901, ECM Biosciences): Applications: WB, IHC, ELISA. Reactivity: mouse, human, rat.
Anti-SSH1L (SP1711, ECM Biosciences): Applications: WB, ELISA, IHC. Reactivity: mouse, human, rat.
Anti-COFILIN Ser3 (3313T, Cell Signaling): Applications: WB, IF, IHC. Reactivity: mouse, human, rat, monkey, bovine.
Anti-COFILIN (5175T, Cell Signaling): Applications: WB, IF, IHC. Reactivity: mouse, human, rat, monkey, dog.
Anti-C/EBPα (8178S, Cell Signaling): Applications: WB, IP, IHC, IF, F. Reactivity: mouse, human.
Anti-GAPDH (sc-32233, Santa Cruz): Applications: WB, IP, IF. Reactivity: mouse, rat, human, rabbit, xenopus.

IHC
Primary antibodies (for murine tissues):
Anti-DIAPH1 (ab11173, Abcam): Applications: IHC, WB. Reactivity: human, mouse.
Anti-CD68 (MCA1957, Biorad): Applications: IHC, IP, WB, IF, ELISA. Reactivity: mouse.
Anti-α-Smooth Muscle Actin (SMA) (PA5-18292, ThermoFisher Scientific): Applications: IHC, WB. Reactivity: mouse, human.
Anti-RAGE (GTX27764, Genetex): Applications: WB, IHC. Reactivity: human, mouse, rat.
Anti-AGE (ab23722, Abcam): Applications: IHC, WB, ELISA. Reactivity: human, mouse, rat.
Anti-DIAPH1 (ab129167, clone EPR7948, Abcam) Applications: Western blot, IHC. Reactivity: mouse, rat, human.

Primary antibodies (for human tissues):
Anti-DIAPH1 (ab11173, Abcam): Applications: IHC, WB. Reactivity: human, mouse.
Anti-CD68 (M0814, Dako): Applications: IHC. Reactivity: human.
Anti-αSMA (A2547, Millipore Sigma): Applications: IHC, WB. Reactivity: human, frog, sheep, chicken, goat, bovine, rat, guinea pig, mouse, canine, rabbit, snake.
Anti-DIAPH1 (ab129167, clone EPR7948, Abcam) Applications: Western blot, IHC. Reactivity: mouse, rat, human.

FC
Primary antibodies:
Anti-CD16/32 (101302, Biolegend): Applications: FC, IP, Block. Reactivity: mouse.
Anti-CD45 - Alexa Fluor 700 (103128, Biolegend): Applications: FC, SB. Reactivity: mouse.
Anti-CD11b - APC-Cy7 (557657, BD Biosciences): Applications: FC. Reactivity: mouse, human.
Anti-CD45R/B220 - FITC (103206, Biolegend): Applications: FC. Reactivity: mouse, human, cat.
Anti-CD3ε - FITC (100306, Biolegend): Applications: FC. Reactivity: mouse.
Anti-CD170 (Siglec-F) - FITC (155504, Biolegend): Applications: FC. Reactivity: mouse.
Anti-LY-6G - FITC (127606, Biolegend): Applications: FC. Reactivity: mouse.
Anti-CD206 - Brilliant Violet 650 (141723, Biolegend): Applications: FC, ICFC. Reactivity: mouse.
Anti-LY-6C - Brilliant Violet 510 (128033, Biolegend): Applications: FC. Reactivity: mouse.

Anti-CD14 - Brilliant Violet 421 (123329, Biolegend): Applications: FC. Reactivity: mouse.
Anti-CD163 - PE (12-1631-82, Invitrogen): Applications: FC. Reactivity: mouse.

Eukaryotic cell lines

Policy information about cell lines and Sex and Gender in Research

Cell line source(s)	The murine hepatocellular carcinoma cell line Hepa 1-6 was purchased from American Type Culture Collection (ATCC® CRL-1830™).
Authentication	Cells were authenticated as per the supplier's product sheet, safety data sheet, and certificate of analysis.
Mycoplasma contamination	Cells were not tested for mycoplasma contamination. Every 7-8 passages cells were discarded and experimental replicates were performed with a new batch of cells.
Commonly misidentified lines (See ICLAC register)	No commonly misidentified cells were used.

Animals and other research organisms

Policy information about studies involving animals; ARRIVE guidelines recommended for reporting animal research, and Sex and Gender in Research

Laboratory animals	All animals used in this study were male and female mice. Mice (C57BL/6J background) were deficient for the low-density lipoprotein receptor (Ldlr—/—) (The Jackson Laboratories, Stock No 002207, Bar Harbor ME) or for Diaphanous 1 (DIAPH1) (Diaph1—/—) 42 backcrossed >20 generations into Ldlr—/—(Ldlr—/—Diaph1—/—).Mice were fed a Western diet (Research Diets, Inc., D01061401Ci; 0.15% cholesterol) for 16 weeks starting at 6 weeks of age, unless otherwise stated.
Wild animals	N/A
Reporting on sex	In this study, we employed both male and female Ldlr—/—Diaph1—/— vs. Ldlr—/— mice.
Field-collected samples	N/A
Ethics oversight	All experiments were performed under protocols approved by the New York University School of Medicine Institute Animal Care Committee (IACUC) in accordance with international and NIH guidelines.

Note that full information on the approval of the study protocol must also be provided in the manuscript.

Flow Cytometry

Plots

Confirm that:

☒ The axis labels state the marker and fluorochrome used (e.g. CD4-FITC).

☒ The axis scales are clearly visible. Include numbers along axes only for bottom left plot of group (a 'group' is an analysis of identical markers).

☒ All plots are contour plots with outliers or pseudocolor plots.

☒ A numerical value for number of cells or percentage (with statistics) is provided.

Methodology

Sample preparation	Aortas were perfused with PBS and aortic arches were collected from experimental mice and whole aortas from controls. Aortas were digested using a cocktail of liberase TH (0.77 mg/ml, Roche, 5401151001), deoxyribonuclease (DNase) I (58 ug/ml, Sigma, DN25), and hyaluranidase (99 ug/ml, Sigma, H3506) in HBSS with 0.5% BSA and 1 mM of calcium solution. Digestion was performed for 15 min at 37ºC using the program m_37SDK1 in the GentleMacs dissociator (Miltenyi). Single cell suspensions were filtered through 100 µM filters (Fisher Scientific, #22363549) and pelleted by centrifugation (400 x g for 5 min at 4°C). Aortic single cell suspensions were live/dead stained with Fixable Blue Dead Cell Stain kit (Invitrogen, L34961) and blocked with CD16/32 for 30 min at 4ºC in the dark. To identify aortic macrophages and monocytes, cells were further incubated with a lineage of antibodies recognizing CD45R, CD3ε, CD170/Siglec-F and Ly-6G (FITC), as well as CD45 (AF700), CD11b (APC-Cy7), CD206 (BV650), CD163 (PE), CD14 (BV421) and Ly-6C (BV510) for 30 min at 4ºC in the dark.
Instrument	Cells were acquired on a LSRII UV (BD Biosciences)
Software	FACSDiva (BD Biosciences) was used to collect the data, and FlowJo 10.8.1 (BD Biosciences) was used to analyzed the flow cytometry data.

Cell population abundance	After removing debris, dead cells and aggregates, single cells were ~55% of total cells. CD45+ cells were ~2-5% of total cells, a total of ~20% of them being macrophages. Subsets of CD206high, CD163high, CD14high and Ly-6Chigh macrophages were analyzed with ~20-40% depending on the marker and genotype.
Gating strategy	Aortic macrophages were identified as live cells, single cells (SSC-H/SSC-W and FSC-H/FSC-W), Lin(FITC)-, CD45+ and CD11b+, and further characterized as either CD206high, CD163high, CD14high and Ly-6Chigh subsets.

☒ Tick this box to confirm that a figure exemplifying the gating strategy is provided in the Supplementary Information.
